# Structure‐Activity Studies Reveal Scope for Optimisation of Ebselen‐Type Inhibition of SARS‐CoV‐2 Main Protease

**DOI:** 10.1002/cmdc.202100582

**Published:** 2021-12-27

**Authors:** Siegfried T. D. Thun‐Hohenstein, Timothy F. Suits, Tika R. Malla, Anthony Tumber, Lennart Brewitz, Hani Choudhry, Eidarus Salah, Christopher J. Schofield

**Affiliations:** ^1^ Department of Chemistry University of Oxford Chemistry Research Laboratory and the Ineos Oxford Institute for Antimicrobial Research 12 Mansfield Road Oxford OX1 3TA UK; ^2^ Department of Biochemistry Center for Artificial Intelligence in Precision Medicines King Abdulaziz University Jeddah Saudi Arabia

**Keywords:** M^pro^ inhibition, SARS-CoV-2, COVID-19, ebselen, ebsulfur, nucleophilic cysteine protease.

## Abstract

The reactive organoselenium compound ebselen is being investigated for treatment of coronavirus disease 2019 (COVID‐19) and other diseases. We report structure‐activity studies on sulfur analogues of ebselen with the Severe Acute Respiratory Syndrome coronavirus 2 (SARS‐CoV‐2) main protease (M^pro^), employing turnover and protein‐observed mass spectrometry‐based assays. The results reveal scope for optimisation of ebselen/ebselen derivative‐ mediated inhibition of M^pro^, particularly with respect to improved selectivity.

## Graphical abstract

**Ebselen reacts covalently** with M^pro^ multiple times, raising questions about selectivity. “Ebsulfur” compounds, in which the selenium of ebselen is replaced with sulfur, have comparable potency to ebselen but covalently react with M^pro^ fewer times, revealing potential for optimisation of the selectivity of ebselen

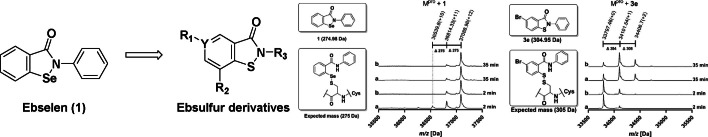

Ebselen is a synthetic organoselenium compound which has been under investigation for the treatment of diseases including stroke, hearing loss, and neurodegenerative disorders.[^1^, ^2^, ^3^] Ebselen has also been proposed as a treatment for bacterial, viral, and fungal infections.[^4^, ^5^, ^6^] Recently, ebselen has attracted attention because of its potential to treat COVID‐19, as shown in cell‐based assays and by its ability to inhibit the SARS‐CoV‐2 main protease (M^pro^), the action of which is essential for the virus life cycle.^[7]^ Ebselen is a potent inhibitor of M^pro^, with an IC_50_ of <100 nM.^[7]^ It is proposed to inhibit M^pro^ via covalent reaction of its N−Se bond, in particular with the M^pro^ nucleophilic active site cysteine (Cys145).

Crystallographic studies on the inhibition of catalytic cysteine enzymes, such as Ldt_Mt2_, by ebselen reveal that an active site nucleophilic cysteine can react with the inhibitor N−Se group to form a catalytically inactive thioselenide, without other fragmentation of the inhibitor.^[8]^ As M^pro^ has a catalytic cysteine, it is possible that a similar process is responsible for the inhibition of M^pro^ (Figure 1). However, a crystal structure with intact ebselen bound to M^pro^ has not been reported and there is crystallographic evidence that ebselen can react with M^pro^ to transfer a selenium atom to the active site cysteine.^[9]^ Mass spectrometric (MS) studies indicate that ebselen can react with multiple nucleophilic residues on M^pro^.^[10]^ The reactivity of the N−Se ebselen bond is likely important in all its reported biological activities, raising questions about its selectivity and potential toxicity.[^11^, ^12^, ^13^]

**Figure 1:**
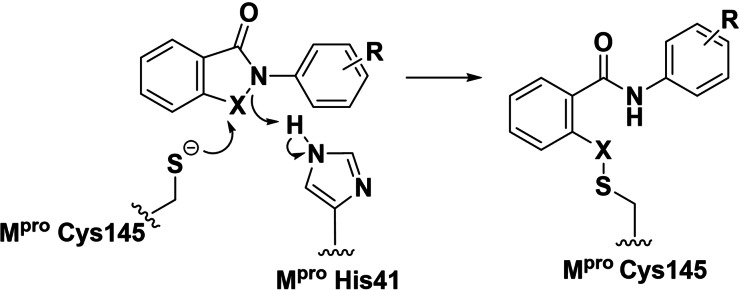
Initial reaction of ebselen/ebsulfur derivatives with the cysteine protease M^pro^. X=S or Se. Ebselen, X=Se, R=H. Note reaction may also occur with non‐catalytic cysteines ‐ M^pro^ has a total of 12 cysteine residues.^[7]^

Here we report initial structure‐activity relationship (SAR) studies on the inhibition of M^pro^ by derivatives of ebselen with a sulfur substituted for the selenium (ebsulfur compounds).^[14]^ The MS analyses reveal ebsulfur reacts more than once with M^pro^, but that some ebsulfur derivatives retain potency against M^pro^ while showing reduced rates of reaction with non‐active site nucleophilic residues. Together with other studies reported during the course of our work,[^14^, ^15^] the results reveal the potential for improvement of ebselen‐type inhibition of M^pro^, perhaps most importantly in terms of selectivity.

The ebsulfur derivatives were synthesised with the aim of investigating their potency and selectivity for the active site Cys145 of M^pro^. The changes made to ebselen included replacing the selenium for a sulfur atom to decrease reactivity, varying the *N*‐linked sidechain, and adding substituents at the C6 and C8 positions (see Table 1) to alter the fit at the M^pro^ active site. The ebsulfur analogues were synthesised (Figure 2) via amide formation between the appropriate acyl chloride and primary amine to give amides **13**–**29**. Subsequent copper‐mediated oxidative sulfur insertion and ring closure as described by Bhakuni et al.^[16]^ gave the cyclised products **3 a**–**c** and **3 e**–**11**. The bromide substituent of **3 c** enabled functionalization by Suzuki‐Miyaura cross‐coupling to give **3 d**. In one case cyclisation of **29** gave the expected product **12 i** as well as the oxidised product **12**, after purification.

**Table 1:**
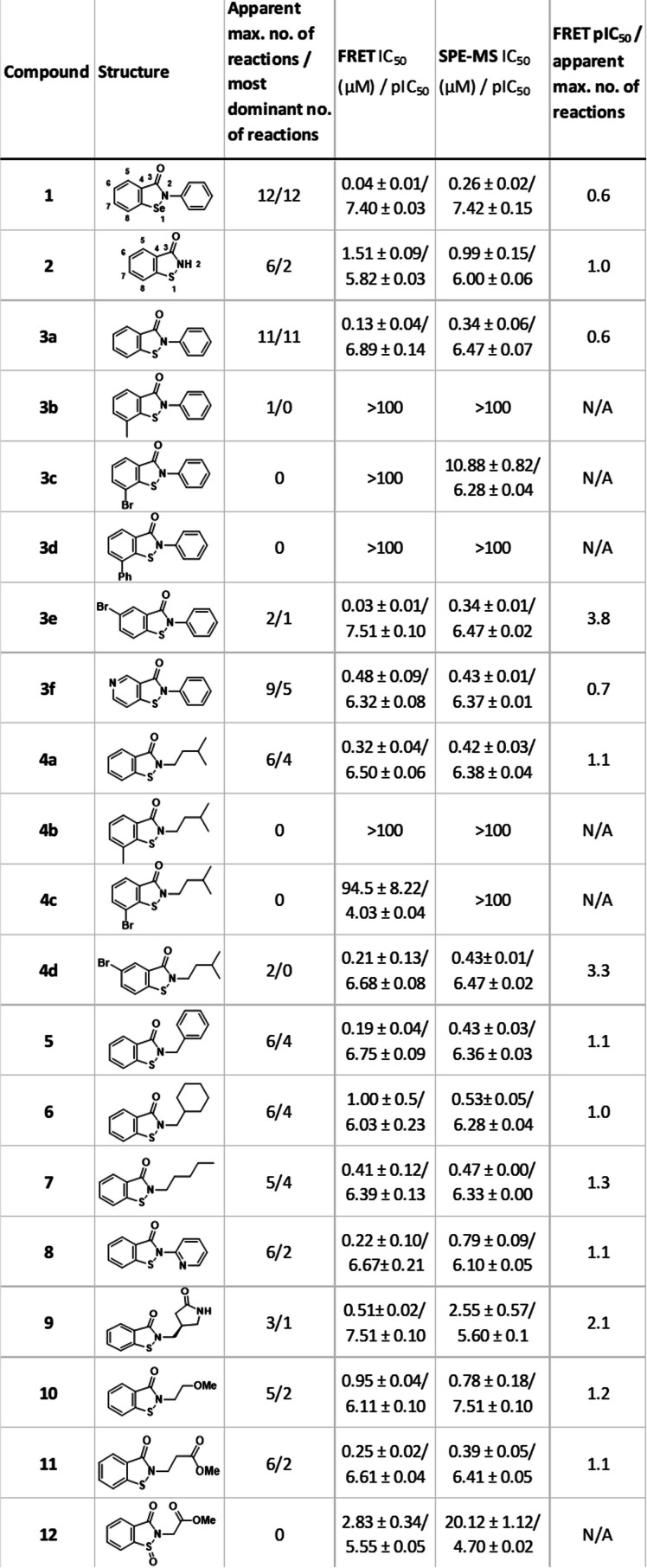
Ebsulfur derivative inhibition and reactivity with M^pro^ via FRET and SPE‐MS assay. See Supporting Information for assay conditions. Note the extent and the number of covalent reactions is likely condition/concentration dependent. See figures S1, S2 and S3 for dose response curves and corresponding covalent modification studies.

**Figure 2:**
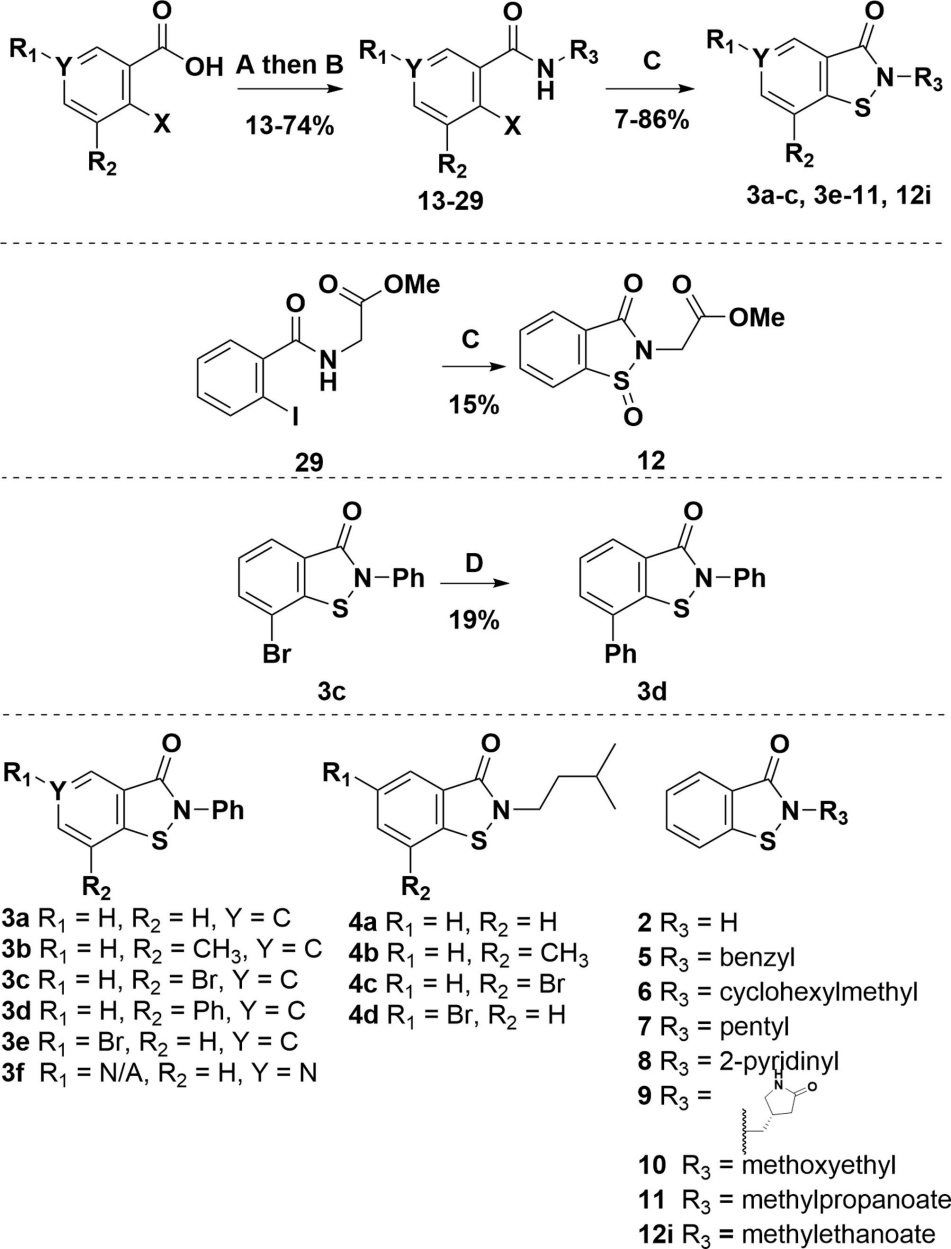
Synthesis of ebsulfur derivatives **3 a**–**f**, **4 a**–**d**, **5**–**12**. Reagents and conditions: **A**: oxalyl chloride, CH_2_Cl_2_, DMF, 0 °C→rt. **B**: Et_3_N, R_3_NH_2_, CH_2_Cl_2_, rt. **C**: CuI, 1,10‐phenanthroline, S_8_, K_2_CO_3_, DMF, 110 °C. **D**: phenylboronic acid, K_2_CO_3_, Pd(dppf)Cl_2_, 1,4‐dioxane, 80 °C.

The ebsulfur compounds were initially screened for M^pro^ inhibition activity using a fluorescence‐based turnover (FRET) assay and a solid‐phase extraction coupled to mass spectrometry (SPE‐MS) assay, and for selectivity in terms of reaction with the catalytic Cys145 of M^pro^ by protein‐observed mass spectrometry (POMS) under denaturing conditions.^[10]^ M^pro^ contains a total of twelve cysteines,^[7]^ hence the number of covalent reactions observed with M^pro^ gives potential insight into the degree of active site selectivity of the ebsulfur compounds.

The turnover assay results (Table 1) show that the direct ebsulfur analogue (**3 a**) of ebselen (**1**) has a comparable IC_50_ to ebselen (**1**), confirming that an S−N bond is sufficiently reactive to inhibit M^pro^ at nanomolar concentrations. The unsubstituted ebsulfur benzoisothiazolinone (BIT) core (**2**) was much less active (IC_50_∼1.6 μM). Ebsulfur derivatives **3 a**, **4 a**, **5**–**7**, varying solely in the *N*‐linked hydrophobic sidechain, were synthesised since it was envisaged that these sidechains might occupy the same binding pocket as the sidechain of the P1′ residue of M^pro^ substrates, as anticipated based on the structures of substrate‐like inhibitors.^[7]^ These compounds were the most potent M^pro^ inhibitors described here, i. e. **3 a** (*N*‐phenyl) ∼0.13 μM; **4 a** (isopentyl) ∼0.32 μM; **5** (*N*‐benzyl) ∼0.19 μM; **6** (*N*‐cyclohexylmethyl) ∼1.00 μM; and **7** (n‐pentyl) ∼0.41 μM. The variations in IC_50_s for the compounds, together with the results of Sun *et al*.^[14]^ show clear scope for further optimisation of the *N*‐substituent of the ebsulfur derivatives (see supplementary Figures S1 and S2).

Several compounds with more polar *N*‐substituents (**8**–**11**) were synthesised to increase solubility and/or selectivity, including **9**, the γ‐lactam of which was envisaged may bind in an analogous manner to the P1 glutamine sidechain in M^pro^ substrates.^[7]^ None of these compounds were as potent as **1** or **3 a**, perhaps reflecting the hydrophobic nature of regions of the M^pro^ active site, including part of the P1 residue binding sub‐site.^[7]^

Increasing the oxidation state of the sulfur substantially increased the IC_50_ (**12**, ∼2.9 μM cf. **11** 0.25 μM), suggesting steric hindrance may be one factor in determining reactivity (Figure 3). We thus made **3 b**–**d**, **4 b, c** with methyl‐, phenyl‐, or bromo‐derivatives at the C8 position.

**Figure 3:**
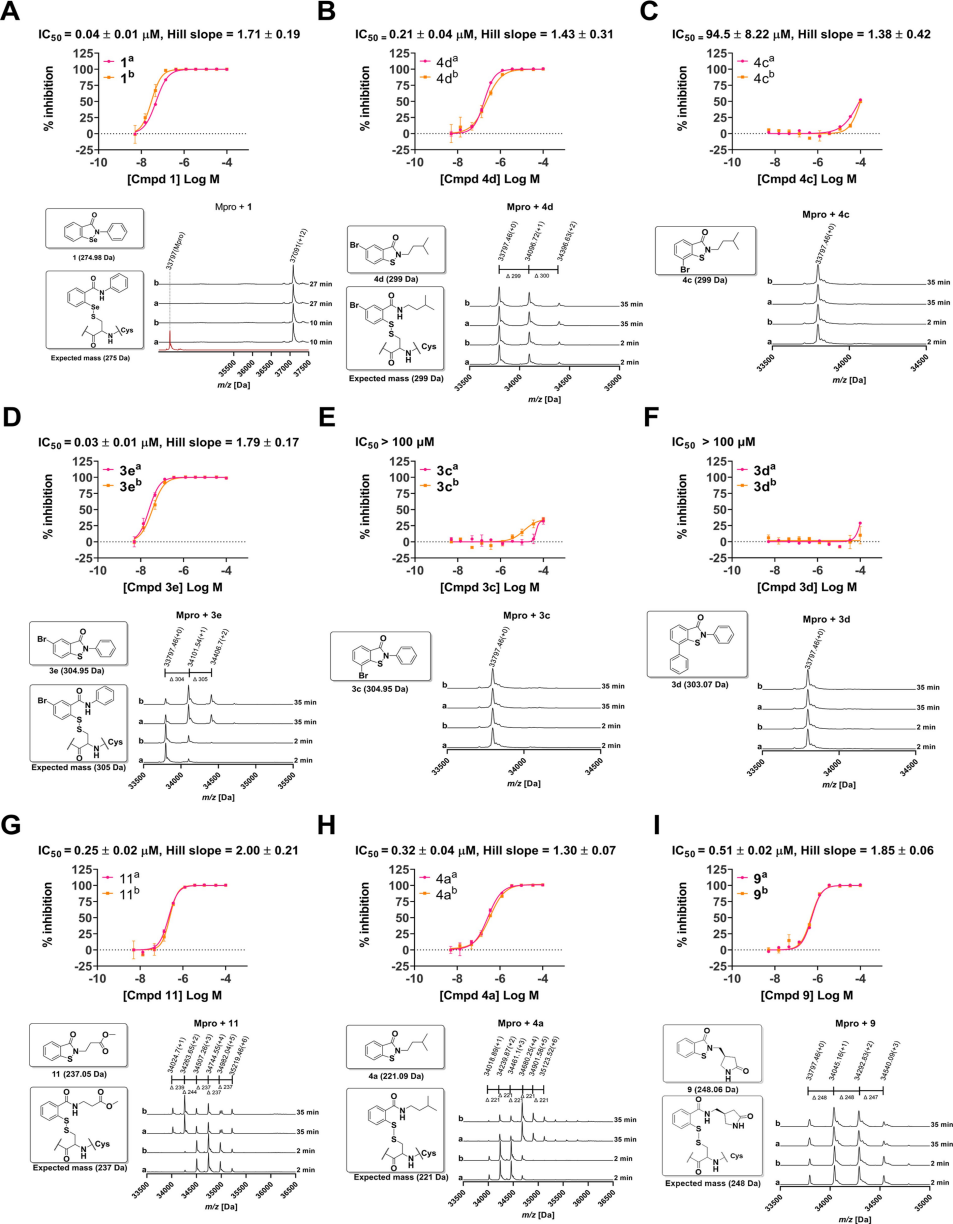
Dose response curves obtained using FRET assay and protein observed mass spectrometry. **A**) 1 (Ebselen) inhibits M^pro^ and reacts at multiple sites. **B**) 4d inhibits M^pro^, but reacts covalently at less sites than ebselen. **C**) 4c (an isomer of 4d) apparently does not inhibit or react with M^pro^. **D**) 3e manifests good inhibition and apparently reacts twice, but predominantly once. **E**) 3c apparently does not inhibit or react with M^pro^. **F**) 3d shows no inhibition and does not react with M^pro^, potentially due to steric hindrance by its C‐8 phenyl group. **G**) 11 inhibits and reacts. **H**) 4a inhibits and reacts, predominantly 4 times. **I**) 9 inhibits M^pro^ and apparently reacts with up to 3 times, but predominantly once. Conditions: 40 μM compound, 2 μM M^pro^, 20 mM HEPES, pH 7.5, 50 mM NaCl. (^a^) and (^b^) represent technical duplicates for POMS studies. See Supporting Information for assay conditions.

Such derivatisation substantially reduced potency: **4 c** has an IC_50_ of ∼94.5 μM, and **3 b**–**d**, and **4 b** have IC_50_s>100 μM. Conversely, C6‐substitution had the opposite effect; addition of a bromine at the C6 position of **3 a** and **4 a**, to give **3 e** and **4 d**, improved potency relative to values for the unsubstituted compounds (**3 e**, ∼0.03 μM cf. **3 a** ∼0.13 μM, and **4 d**, ∼0.21 μM cf. **4 a**, ∼0.32 μM). Introduction of a nitrogen at the C6 position to give the pyridine (**3 f**) showed a slight decrease in potency (IC_50_∼0.5 μM).

It is important to note that it cannot be assumed that inhibition is mediated via reaction with Cys145 alone and derivatisation may alter the patterns of reactivity with the 12 M^pro^ cysteines. We thus carried out protein‐observed mass spectrometry (MS) under denaturing conditions to compare the number of (irreversible) covalent reactions by the ebsulfur derivatives. Although we did not carry out time‐course studies (except for ebselen and ebsulfur^[10]^), and the nature and the extent of the reactions will likely vary depending on the conditions, the results give initial insight into the relative reactivity of the compounds.

The results show that there is a general, but clearly imperfect, correlation between the apparent extent of covalent reaction and potency, consistent with the proposed mode of reaction of the ebsulfur derivatives with cysteines to form disulfide bonds (Figure 1). However, it should be noted that alternative modes of covalent reaction are possible, e. g. via reaction of the ebsulfur derivative carbonyl group. Secondly, introduction of substituents at the C6 and C8 positions of ebsulfur decreases the number of reactions. Thus the presence of bromo‐ or phenyl‐ substituents at C8 apparently completely prevents irreversible covalent reaction (**3 c**, **3 d**, **4 c**). C8 methyl substitution shows a reduction in the apparent maximum number of observed reactions from 6 (**4 a**) to 0 (**4 b**) for the *N*‐isopentyl sidechain, and from 11 (**3 a**) to 1 (**3 b**) for the *N*‐phenyl sidechain; increasing the oxidation state of the sulfur also apparently eliminates observed covalent reaction (**12**). Importantly, the presence of a more polar *N*‐substituent (compare **9**, **11**/1 and 2 reactions, with **6**, **7**/both 4 reactions) or C6 bromine (compare **3 e**/1 reaction with **3 a**/11 reactions, and **4 d**/no reaction with **4 a**/4 reactions) reduces the dominant number of observed reactions. The combined IC_50_ and MS results for **9**, **4 d**, and, particularly, for **3 e**, are notable as they suggest that optimised substitution of the ebsulfur core to achieve enhanced selectivity in terms of its reaction with M^pro^ should be possible. We suggest that optimising the pIC_50_/number of reactions observed by MS as a useful, readily obtainable parameter for initial optimisation of reaction selectivity (Table 1).

The reactivity of ebselen or ebselen/ebsulfur derivatives with cysteines, and maybe other biomolecules, means that there are likely substantial challenges in developing these compounds as medicines. While the activities of the ebsulfur compounds reported here do not improve on the potency of ebselen, they clearly demonstrate the potential for achieving compounds that are more selective in their reaction with M^pro^ and, by implication, other biomolecules including both proteins and small‐molecule thiols such as glutathione/cysteine. This is clearly exemplified by comparing the MS results for ebselen, which, under our standard conditions, covalently reacts up to 12 times with M^pro^, with those for **3 e**, which was observed to only react twice under our conditions, yet retains substantial potency (∼0.03 μM).

Under our conditions, we saw no evidence for the fragmentation of the ebsulfur derivatives or ebselen, as has been provided on the basis of crystallographic and other evidence for ebselen itself,^[9]^ potentially in part reflecting differences in reaction conditions/methods of analysis. We suggest that if such improvements in selectivity can be achieved by simple sulfur for selenium substitution, then there is considerable scope for more sophisticated ‘caging’ of the reactive N−Se/S bonds of ebselen/ebsulfur type compounds. Induced‐fit binding to M^pro^ or another target could result in exposure of a reactive N−S/Se bond, which is otherwise protected from nucleophilic attack by other endogenous cysteines. It is also likely the potency and selectivity of the compounds can be improved by taking advantage of interactions observed with other types of M^pro^ inhibitor, e. g. incorporation of a benzisothiazolinone core into a larger structure that mimics the binding of the natural substrates.

## Conflict of interest

The authors declare no conflict of interest.

## Supporting information

As a service to our authors and readers, this journal provides supporting information supplied by the authors. Such materials are peer reviewed and may be re‐organized for online delivery, but are not copy‐edited or typeset. Technical support issues arising from supporting information (other than missing files) should be addressed to the authors.

Supporting Information

## References

[1] G. K. Azad , R. S. Tomar , Mol. Biol. Rep. 2014, 41(8), 4865–4879. 24867080 10.1007/s11033-014-3417-x

[2] M. J. Parnham , H. Sies , Biochem. Pharmacol. 2013, 86(9), 1248–1253. 24012716 10.1016/j.bcp.2013.08.028

[3] J. Kil , E. Lobarinas , C. Spankovich , S. K. Griffiths , P. J. Antonelli , E. D. Lynch , C. G. Le Prell , Lancet 2017, 390(10098), 969–979. 28716314 10.1016/S0140-6736(17)31791-9

[4] H. Sies , M. J. Parnham , Free Radical Biol. Med. 2020, 156, 107–112. 32598985 10.1016/j.freeradbiomed.2020.06.032 PMC7319625

[5] S. Thangamani , W. Younis , M. N. Seleem , Sci. Rep. 2015, 5, 11596. 26111644 10.1038/srep11596 PMC4481386

[6] S. Thangamani , H. E. Eldesouky , H. Mohammad , P. E. Pascuzzi , L. Avramova , T. R. Hazbun , M. N. Seleem , Biochim. Biophys. Acta Gen. Subj. 2017, 1861(1), 3002–3010. 27712973 10.1016/j.bbagen.2016.09.029 PMC5148707

[7] Z. Jin , X. Du , Y. Xu , Y. Deng , M. Liu , Y. Zhao , B. Zhang , X. Li , L. Zhang , C. Peng , Y. Duan , J. Yu , L. Wang , K. Yang , F. Liu , R. Jiang , X. Yang , T. You , X. Liu , X. Yang , F. Bai , H. Liu , X. Liu , L. W. Guddat , W. Xu , G. Xiao , C. Qin , Z. Shi , H. Jiang , Z. Rao , H. Yang , Natur 2020, 582(7811), 289–293. 10.1038/s41586-020-2223-y 32272481

[8] M. de Munnik , C. T. Lohans , P. A. Lang , G. W. Langley , T. R. Malla , A. Tumber , C. J. Schofield , J. Brem , Chem. Commun. 2019, 55(69), 10214–10217. 10.1039/c9cc04145a PMC6984337 31380528

[9] K. Amporndanai , X. Meng , W. Shang , Z. Jin , M. Rogers , Y. Zhao , Z. Rao , Z.-J. Liu , H. Yang , L. Zhang , P. M. O'Neill , S. Samar Hasnain , Nat. Commun. 2021, 12(1), 3061. 34031399 10.1038/s41467-021-23313-7 PMC8144557

[10] T. R. Malla , A. Tumber , T. John , L. Brewitz , C. Strain-Damerell , C. D. Owen , P. Lukacik , H. T. H. Chan , P. Maheswaran , E. Salah , F. Duarte , H. Yang , Z. Rao , M. A. Walsh , C. J. Schofield , Chem. Commun. 2021, 57(12), 1430–1433. 10.1039/d0cc06870e PMC8006714 33462575

[11] F. C. Meotti , V. C. Borges , G. Zeni , J. B. T. Rocha , C. W. Nogueira , Toxicol. Lett. 2003, 143(1), 9–16. 12697375 10.1016/s0378-4274(03)00090-0

[12] C. Jacob , W. Maret , B. L. Vallee , Biochem. Biophys. Res. Commun. 1998, 248(3), 569–573. 9703967 10.1006/bbrc.1998.9026

[13] R. Sekirnik , N. R. Rose , A. Thalhammer , P. T. Seden , J. Mecinović , C. J. Schofield , Chem. Commun. 2009, 42, 6376–6378. 10.1039/b916357c 19841782

[14] L.-Y. Sun , C. Chen , J. Su , J.-Q. Li , Z. Jiang , H. Gao , J.-Z. Chigan , H.-H. Ding , L. Zhai , K.-W. Yang , Bioorg. Chem. 2021, 112, 104889. 33915460 10.1016/j.bioorg.2021.104889 PMC8026246

[15] M. Zmudzinski , W. Rut , K. Olech , J. Granda , M. Giurg , M. Burda-Grabowska , L. Zhang , X. Sun , Z. Lv , D. Nayak , M. Kesik-Brodacka , S. K. Olsen , R. Hilgenfeld , M. Drag , bioRxiv 2020. doi: https://doi.org/10.1101/2020.08. 30. 273979.

[16] B. S. Bhakuni , S. J. Balkrishna , A. Kumar , S. J. T. L. Kumar , Tetrahedron Lett. 2012, 53(11), 1354–1357.

