# Supplementary material for: Structure‐Activity Studies Reveal Scope for Optimisation of Ebselen‐Type Inhibition of SARS‐CoV‐2 Main Protease
Source: ChemMedChem. 2021 Dec 27;17(4):e202100582. doi: 10.1002/cmdc.202100582 (PMC9015279; doi:10.1002/cmdc.202100582)
Supplement: Supplementary file 1 — Supporting Information [file CMDC-17-0-s001.pdf]

ChemMedChem

Supporting Information

Structure-Activity Studies Reveal Scope for Optimisation of Ebselen-Type Inhibition of SARS-CoV-2 Main Protease

Siegfried T. D. Thun-Hohenstein[+], Timothy F. Suits[+], Tika R. Malla, Anthony Tumber, Lennart Brewitz, Hani Choudhry, Eidarus Salah, and Christopher J. Schofield*

Supplementary information for

Structure-Activity Studies Reveal Scope for Optimisation of Ebselen-Type Inhibition of SARS-CoV-2 Main Protease

Siegfried T. D. Thun-Hohenstein,[1[a]] Timothy F. Suits,[1[a]] Tika R. Malla,[a] Anthony Tumber,[a] Lennart Brewitz,[a] Hani Choudhry,[b] Eidarus Salah,[a] and Christopher J. Schofield.[a]*

Email: christopher.schofield@chem.ox.ac.uk

[a] *Department of Chemistry, Chemistry Research Laboratory and the Ineos Oxford Institute for Antimicrobial Research, University of Oxford, 12 Mansfield Road, Oxford, OX1 3TA, United Kingdom.*

[b] *Department of Biochemistry, Centre for Artificial Intelligence in Precision Medicines, King Abdulaziz University, Jeddah, Saudi Arabia*.

[1]The contributions of these authors should be considered equal.

Table of Contents

1. IC_{50} Data **S2**

1.1. FRET assay IC_{50} curves **S2**

1.2. RFMS assay IC_{50} curves **S3**

2. Protein-Observed MS data **S4**

3. Compound Synthesis **S5**

3.1. General information **S5**

3.2.Synthesis and characterisation of compounds **S6**

4. Biochemical assays **S25**

5. References **S26**

6. NMR Spectra **S28**

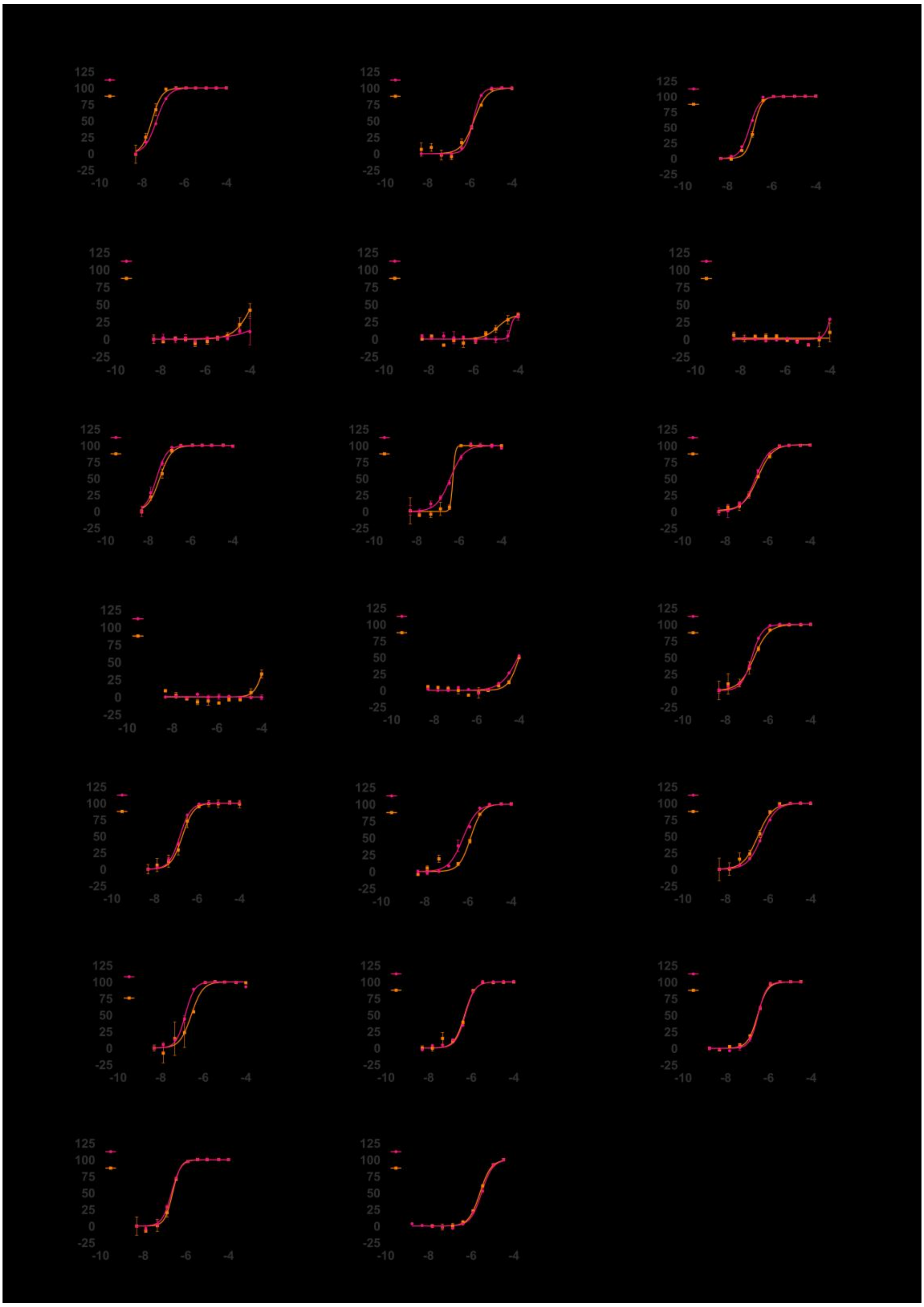

Figure S 1 – Dose response curves obtained using the FRET assay for compounds 1-12. **A) 1 (**ebselen). **B) 2**. **C) 3a**. **D) 3b**. **E) 3c**. **F) 3d**. **G) 3e**. **H) 3f**. **I) 4a**. **J) 4b**. **K) 4C**. **L) 4d**. **M) 5**. **N) 6**. **O) 7**. **P) 8**. **Q) 9**. **R) 10**. **S) 11**. **T) 12**. ([a]) and ([b]) represent independent repeats each composed of technical duplicates for dose response curves. See page S26 for assay conditions.

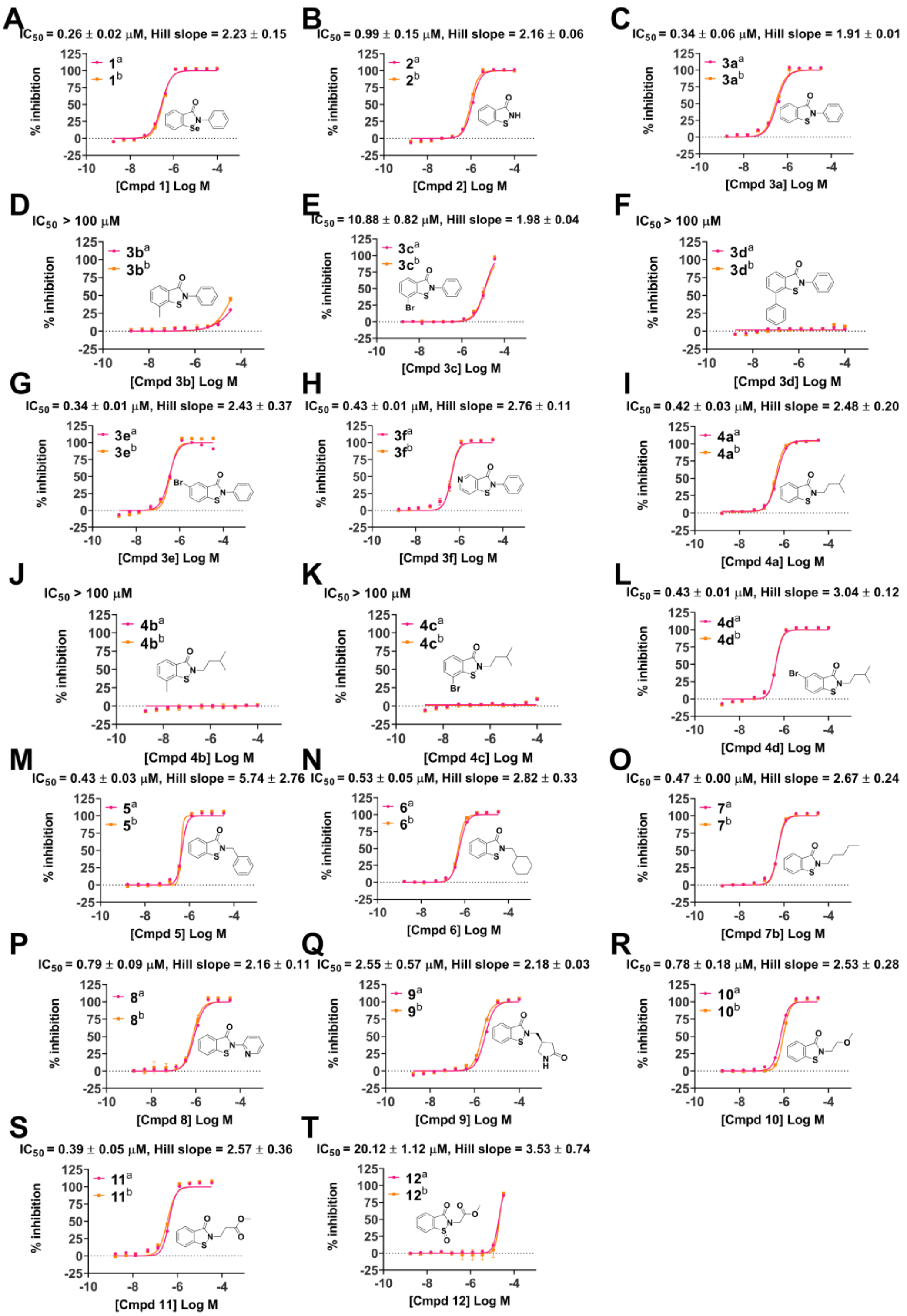

Figure S 2 – ***Dose response curves obtained using the SPE-MS assay for compounds 1-12*****. A) 1 (**ebselen). **B) 2. C) 3a. D) 3b. E) 3c. F) 3d. G) 3e. H) 3f. I) 4a. J) 4b. K) 4C. L) 4d. M) 5. N) 6. O) 7. P) 8. Q) 9. R) 10. S) 11. T) 12**. ([a]) and ([b]) represent independent repeats each composed of technical duplicates for dose response curves. See Supplementary page S26 for assay conditions.

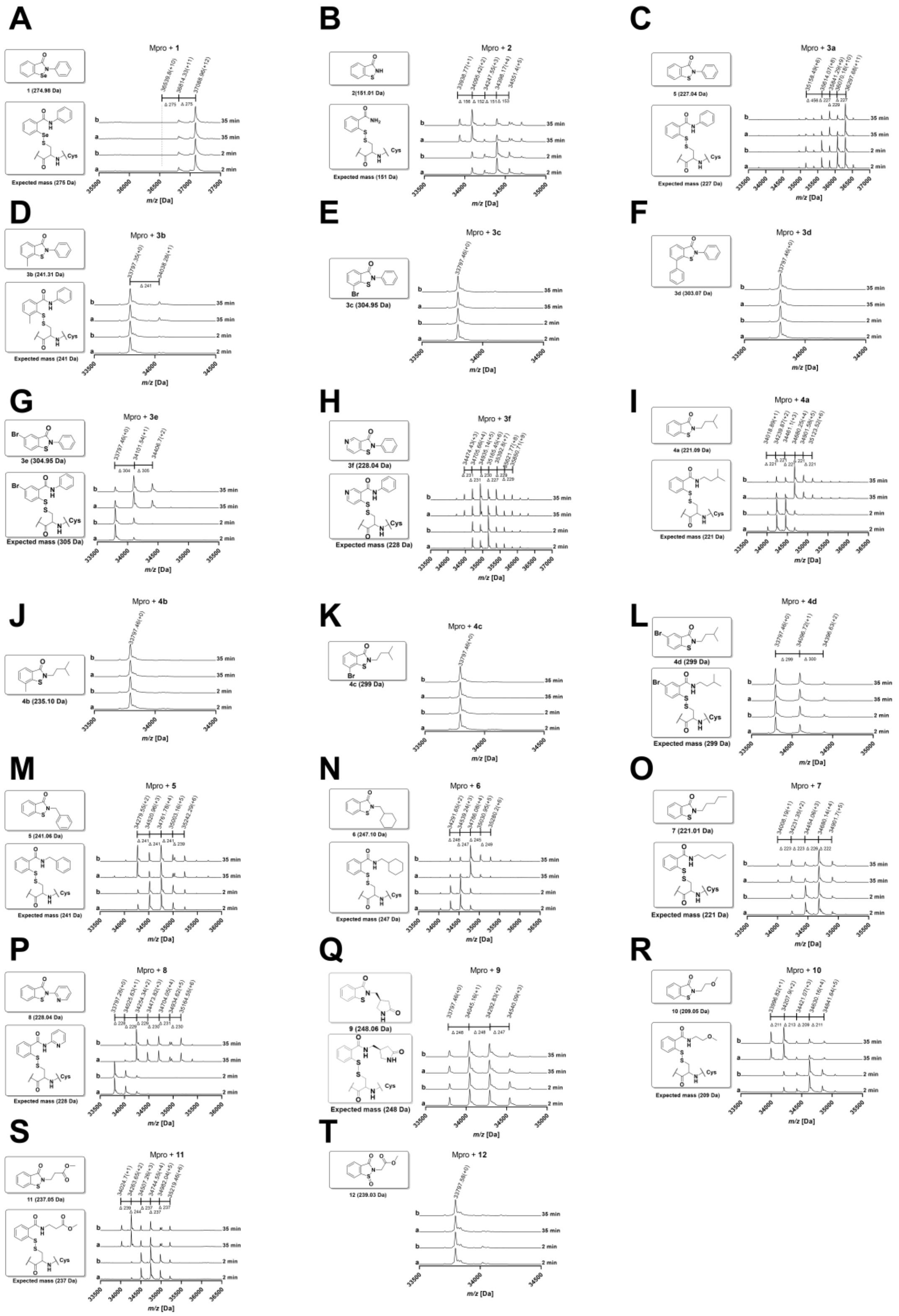

Figure S 3 - ***Protein-Observed mass spectrometry for compounds 1-12*****. A) 1 (**ebselen). **B) 2**. **C) 3a**. **D) 3b**. **E) 3c**. **F) 3d**. **G) 3e**. **H) 3f**. **I) 4a**. **J) 4b**. **K) 4c**. **L) 4d**. **M) 5**. **N) 6**. **O) 7**. **P) 8**. **Q) 9**. **R) 10**. **S) 11**. **T) 12**. Protein mass spectrometry conditions: 40 µM compound, 2 µM M^{pro}, 20 mM HEPES, pH 7.5, 50 mM NaCl. (^{a}) and (^{b}) represent independent repeats each composed of technical duplicates for protein observed mass spectrometry data. See page S26 for assay conditions.

Compound Synthesis

General information

Commercially available reagents and solvents were from Merck, Fluorochem, or Activate Scientific, and were used as received. All manipulations with air- and moisture-sensitive compounds were carried out under a positive pressure of argon in flame-dried glassware.

Chromatographic separation/purifications were performed using a Biotage Isolera system. Unless otherwise specified, the solvent system employed a 0 to 40% (v/v, EtOAc/Cyclohexane) gradient using Biotage Sfär® HC Duo cartridges. Reactions were monitored by TLC on Merck aluminium backed sheets coated with Merck Kieselgel 60 F254 (230-400 mesh) fluorescent treated silica, and visualised under ultra-violet light (254 nm or 365 nm) and/or stained with ninhydrin.

Melting points were taken on a Stuart® SMP40 automatic melting point instrument. NMR-spectra were acquired using a 2-channel Bruker AVIIIHD 400 machine equipped with a 5 mm BBFO probe. Chemical shifts were referenced to residual protio- and perdeuterio-solvent resonances (δ_H 7.26 and δ_C 77.16 for $CDCl_3$; δ_H 2.50 and δ_C 39.52 for DMSO-d_6; δ_H 3.31 and δ_C 49.00 for CD_3OD) as internal standards for ^{1}H NMR and ^{13}C NMR spectra, respectively. All NMR spectra were processed using MestReNova software v. 14.1.

LC-MS (liquid chromatography-mass spectrometry) data were obtained using an Agilent 1200 series LC machine connected to an Agilent 6120 Quadrupole MS using electrospray ionization (ESI) machine. LC was performed on a C18 reversed-phase column (Acquity UPLC BEH C18, 2.1 mm × 50 mm, 1.7 µm) operated at 30°C, using a linear gradient of the binary solvent system of buffer A (H_2O:formic acid, 100:0.1 v/v) to buffer B (MeCN:formic acid, 100:0.1 v/v) from 0 to 100% B in 8.5 min, then 1 min at 100% B, maintaining a flow rate of 1.5 mL/min. HR-MS (high resolution mass spectrometry) were obtained using a Bruker µTOF (ESI) spectrometer.

Specific rotations were measured using a Schmidt-Haensch UniPol polarimeter. Values are expressed in 10^{-1} deg cm^2 g^{-1}; concentrations (c) are quoted in g/100 mL; D refers to the D-line of sodium (589 nm).

Synthesis and characterization of compounds

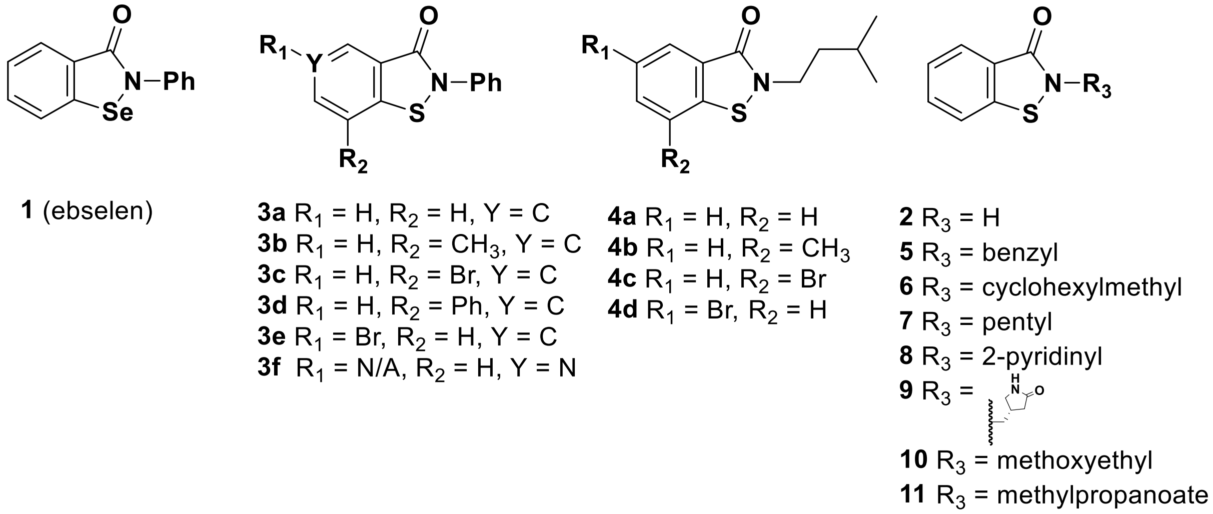

Figure S4. Compounds tested as M^{pro} inhibitors. Ebselen **1**, benzoisothiazolinone (BIT) **2**, Ebsulfur derivatives **3a-f, 4a-d, 5-12**.

General Procedure A – Formation of acyl chlorides (14-17, 19-21)

Oxalyl chloride (1.1 equiv) was added slowly to a solution (or suspension) of the requisite aryl carboxylic acid (1 equiv) in anhydrous CH_2Cl_2 (0.3 M) at 0 °C, followed by addition of 1 drop of anhydrous DMF. The mixture was stirred for 1 h at 0 °C. The cooling bath was then removed and the mixture was stirred for a further 2 h at room temperature (rt) (consumption of the starting aryl carboxylic acid was monitored by TLC and LCMS). The solution was carried forward to the next step without purification.

General Procedure B – Amide coupling (13-29)

Triethylamine (2 equiv) was either added to the requisite acyl chloride (1 equiv) in CH_2Cl_2 (0.3 M) (**13**, **18**, **22-29**), or acyl chloride solution using General Procedure A (**14-17**, **19-21**), followed by addition of the requisite primary amine (1.1 equiv). The mixture was stirred at room temperature overnight (consumption of the starting acyl chloride was monitored by TLC and/or LCMS). The reaction was then diluted with EtOAc; the resultant solution was washed with 1 M HCl, then brine. The organic phase was dried over Na_2SO_4, then concentrated *in vacuo*. Purification was achieved using flash chromatography to yield the desired compound.

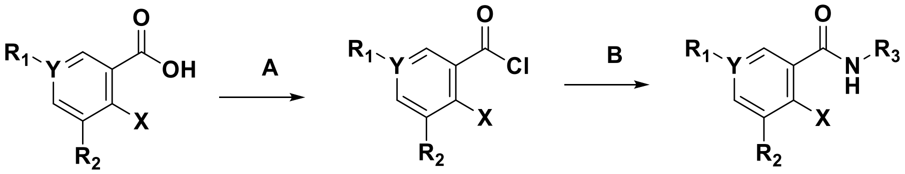

Figure S5. Synthesis of the secondary amide intermediates 13-29. Conditions; A: $(COCl)_2$, DMF, CH_2Cl_2, 0 °C; B: R_3-NH_2, Et_3N, CH_2Cl_2, rt.

2-Iodo-*N*-phenylbenzamide (13)[1]

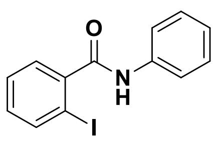

Application of General Procedure B using 2-iodobenzoyl chloride (267 mg, 1 mmol) and aniline (100 µL, 1.1 mmol) yielded 250 mg (74%) of **13** as a white solid.

IR (neat): ν (cm^{-1}) 3058, 2921, 1655, 1619, 1540, 1440, 1325; **^{1}H NMR** (400 MHz, $CDCl_3$) δ 7.91 (dd, *J* = 8.0, 1.0 Hz, 1H), 7.67 – 7.60 (m, 2H), 7.53 (dd, *J* = 7.5, 1.5 Hz, 1H), 7.46-7.36 (m, 4H), 7.23 – 7.11 ppm (m, 2H); **^{13}C NMR** (101 MHz, $CDCl_3$) δ 167.18, 142.15, 140.08, 137.51, 131.54, 129.17, 128.58, 128.39, 124.95, 120.10, 92.35 ppm; **ESI HR-MS**: calcd. for $[C_{13}H_{10}INO+Na]^+$: 345.9699, found: 345.9699. The spectroscopic data match those reported in the literature.[1]

2-Iodo-3-methyl-*N*-phenylbenzamide (14)[2]

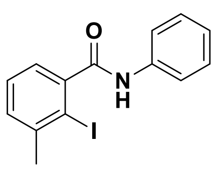

Application of General Procedure A using 2-iodo-3-methylbenzoic acid (262 mg, 1 mmol), gave the crude acyl chloride, which was used in the next step without purification.

Application of General Procedure B using triethylamine (167 µL, 1.2 mmol) and aniline (104 µL, 1.1 mmol) yielded 193 mg (57%) of **14** as a beige solid.

Mp 149-150 °C; **IR (neat):** ν (cm^{-1}) 3196, 3076, 3017, 2919, 2850, 1647, 1596, 1490, 1335, 1013; **^{1}H NMR** (400 MHz, $CDCl_3$) δ 7.65 – 7.61 (m, 2H), 7.43 – 7.35 (m, 3H), 7.33 – 7.28 (m, 2H), 7.27 – 7.22 (m, 1H), 7.21 – 7.14 (m, 1H), 2.51 ppm (s, 3H); **^{13}C NMR** (101 MHz, $CDCl_3$) δ 168.36, 143.96, 143.23, 137.78, 130.94, 129.29, 128.44, 125.39, 125.00, 120.23, 99.36, 29.33

ppm; **ESI HR-MS**: calcd. for $[C_{14}H_{12}INO+H]^+$: 338.0036, found: 338.0038. The spectroscopic data match those reported in the literature.[2]

2,3-Dibromo-*N*-phenylbenzamide (15)

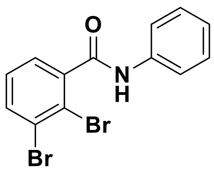

Application of General Procedure A using 2,3-dibromobenzoic acid (280 mg, 1 mmol), gave the crude acyl chloride solution, which was used in the next step without purification.

Application of General Procedure B using triethylamine (167 µL, 1.2 mmol) and aniline (104 µL, 1.1 mmol) yielded 180 mg (51%) of **15** as a beige solid.

Mp 152-153 °C; **IR (neat):** ν (cm^{-1}) 3201, 3138, 3065, 2962, 2850, 1605, 1578, 1439, 1334; **^{1}H NMR** (400 MHz, $CDCl_3$) δ 7.72 (dd, *J* = 8.0, 1.6 Hz, 1H), 7.64 – 7.58 (m, 2H), 7.55 (s, 1H), 7.47 (dd, *J* = 7.6, 1.6 Hz, 1H), 7.42 – 7.35 (m, 2H), 7.29 – 7.23 (m, 1H), 7.22 – 7.15 ppm (m, 1H); **^{13}C NMR** (101 MHz, $CDCl_3$) δ 165.46, 140.94, 137.45, 135.29, 129.35, 128.92, 127.74, 126.90, 125.26, 122.08, 120.26 ppm; **ESI HR-MS**: calcd. for $[C_{13}H_9Br_2NO+H]^+$: 355.9103, found: 355.9105.

2,5-Dibromo-*N*-phenylbenzamide (16)[3]

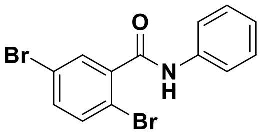

Application of General Procedure A using 2,5-dibromobenzoic acid (625 mg, 2.23 mmol), gave the crude acyl chloride solution, which was used in the next step without purification.

General Procedure B was followed with triethylamine (370 µL, 2.68 mmol, 1.2 equiv) and aniline (232 µL, 2.46 mmol). Purification by reverse-phase chromatography using a Biotage Sfär® C18 Duo (30 g) cartridge with a solvent system of buffer A (H_2O:formic acid, 100:0.1 v/v) to buffer B (MeCN:formic acid, 100:0.1 v/v) from 0 to 100% B over 29 column volumes, then 7 column volumes at 100% B. Concentration *in vacuo* yielded 553 mg (70%) of **16** as a beige solid.

Mp 149-150 °C; **IR (neat):** ν (cm^{-1}) 3088, 2960, 2921, 1655, 1548, 1444, 1327, 1029; **^{1}H NMR** (400 MHz, $CDCl_3$) δ 7.77 (d, *J* = 2.3 Hz, 1H), 7.69 (s, 1H), 7.64 – 7.60 (m, 2H), 7.49 (d, *J* = 8.5 Hz, 1H), 7.43 (dd, *J* = 8.5, 2.4 Hz, 1H), 7.39 (t, *J* = 7.9 Hz, 2H), 7.22 – 7.16 ppm (m, 1H); **^{13}C NMR** (101 MHz, $CDCl_3$) δ 164.11, 139.46, 137.32, 135.07, 134.77, 132.82, 129.35, 125.33, 121.91, 120.32, 118.06 ppm; **ESI HR-MS**: calcd. for $[C_{13}H_9Br_2NO+H]^+$: 353.9123, found 353.9125. The spectroscopic data match those reported in the literature.[3]

4-Chloro-*N*-phenylpyridine-3-carboxamide (17)

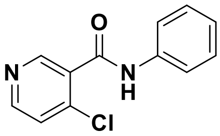

Application of General Procedure A using 4-chloro-nicotinic acid (201 mg, 1 mmol), gave the crude acyl chloride solution, which was used in the next step without purification.

Application of General Procedure B using triethylamine (167 µL, 1.2 mmol, 1.2 equiv) and aniline (104 µL, 1.1 mmol) yielded 80 mg (34%) of **17** as an orange wax.

IR (neat): ν (cm^{-1}) 3017, 2981, 2900, 1635, 1558, 1419, 1305, 1083; **^{1}H NMR** (400 MHz, CDCl3) δ 8.90 (s, 1H), 8.56 (d, *J* = 5.4 Hz, 1H), 8.09 (s, 1H), 7.64 (dd, 2H), 7.42 – 7.36 (m, 3H), 7.20 ppm (m, 1H) **^{13}C NMR** (101 MHz, $CDCl_3$) δ 162.27, 152.03, 150.94, 141.07, 137.31, 131.32, 129.37, 125.45, 125.20, 120.42 ppm; **ESI HR-MS**: calcd. for $[C_{12}H_9ClN_2O+H]^+$: 233.0476, found: 233.0477.

2-Iodo-*N*-(3-methylbutyl)benzamide (18)[4]

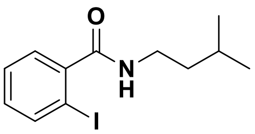

Application of General Procedure B using triethylamine (91 µL, 0.65 mmol, 0.65 equiv), 2-iodobenzoyl chloride (266 mg, 1 mmol) and 3-methylbutylamine (126 µL, 1.1 mmol) yielded 206 mg (65%) of **18** as a beige solid.

Mp 74-75 °C; **IR (neat):** ν (cm^{-1}) 3031, 2920, 2850, 1647, 1586, 1300, 1016; **^{1}H NMR** (400 MHz, $CDCl_3$) δ 7.91 – 7.79 (m, 1H), 7.42 – 7.31 (m, 2H), 7.08 (m, 1H), 5.75 (s, 1H), 3.46 (ddd, *J* = 8.5, 7.4, 5.8 Hz, 2H), 1.73 (m, 1H), 1.57 – 1.47 (m, 1H), 0.95 ppm (d, J = 6.6 Hz, 6H); **^{13}C NMR** (101 MHz, $CDCl_3$) δ 169.45, 142.68, 139.93, 131.12, 128.39, 128.29, 92.55, 38.56,

38.34, 26.04, 22.58 ppm; **ESI HR-MS**: calcd. for $[C_{12}H_{16}INO+H]^+$: 318.0349, found: 318.0350. The spectroscopic data match those reported in the literature.[4]

2-Iodo-3-methyl-*N*-(3-methylbutyl)benzamide (19)

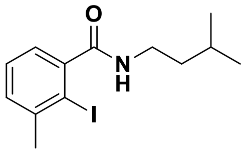

Application of General Procedure A using 2-iodo-3-methylbenzoic acid (262 mg, 1 mmol), gave the crude acyl chloride solution, which was used in the next step without purification.

Application of General Procedure B using triethylamine (167 µL, 1.2 mmol) and 3-methylbutylamine (126 µL, 1.1 mmol) yielded 230 mg (69%) of **19** as a white solid.

Mp 103-104 °C; **IR (neat):** ν (cm^{-1}) 3267, 3085, 2951, 1633, 1555, 1442, 1338, 1012; **^{1}H NMR** (400 MHz, $CDCl_3$) δ 7.29 – 7.19 (m, 2H), 7.14 – 7.05 (m, 1H), 5.65 (s, 1H), 3.52 – 3.42 (m, 2H), 2.48 (d, *J* = 0.7 Hz, 3H), 1.79 – 1.65 (m, 1H), 1.57 – 1.47 (m, 1H), 0.96 ppm (d, *J* = 6.6 Hz, 6H); **^{13}C NMR** (101 MHz, $CDCl_3$) δ 170.50, 144.39, 142.97, 130.45, 128.24, 125.12, 99.47, 38.52, 38.32, 29.30, 26.05, 22.59 ppm; **ESI HR-MS**: calcd. for $[C_{13}H_{18}INO+H]^+$: 332.0505, found 332.0504.

2,3-Dibromo-*N*-(3-methylbutyl)benzamide (20)

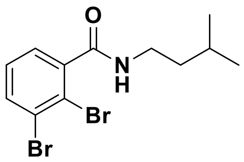

Application of General Procedure A using 2,3-dibromobenzoic acid (280 mg, 1 mmol), gave the crude acyl chloride solution, which was used in the next step without purification.

Application of General Procedure B was followed using triethylamine (167 µL, 1.2 mmol, 1.2 equiv) and 3-methylbutylamine (126 µL, 1.1 mmol) yielded 149 mg (43%) of **20** as a white solid.

Mp 104-106 °C; **IR (neat):** ν (cm^{-1}) 3262, 3052, 2954, 2868, 1640, 1444, 1309, 1230, 1055; **^{1}H NMR** (400 MHz, $CDCl_3$) δ 7.67 (dd, *J* = 8.0, 1.6 Hz, 1H), 7.35 (dd, *J* = 7.6, 1.6 Hz, 1H), 7.23 – 7.18 (m, 1H), 5.78 (s, 1H), 3.52 – 3.42 (m, 2H), 1.70 (m, 1H), 1.57 – 1.47 (m, 2H), 0.96 ppm (d, J = 6.6 Hz, 6H); **^{13}C NMR** (101 MHz, $CDCl_3$) δ 167.64, 141.24, 134.84, 128.74,

127.55, 126.66, 122.01, 38.66, 38.29, 26.05, 22.57 ppm; **ESI HR-MS**: calcd. for [$C_{12}H_{15}Br_2NO+H]^+$: 347.9593, found 347.9595. The spectroscopic data matches those reported in the literature.[4]

2,5-Dibromo-*N*-(3-methylbutyl)benzamide (21)

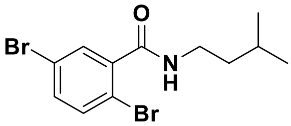

Application of General Procedure A using 2,5-dibromobenzoic acid (280 mg, 1 mmol), gave the crude acyl chloride solution, which was used in the next step without purification.

Application of General Procedure B using triethylamine (167 µL, 1.2 mmol) and 3-methylbutylamine (126 µL, 1.1 mmol) yielded 166 mg (48%) of **21** as a beige solid.

Mp 117-118 °C; **IR (neat):** ν (cm^{-1}) 3264, 3077, 2957, 2851, 1649, 1548, 1369, 1155, 1085; **^{1}H NMR** (400 MHz, $CDCl_3$) δ 7.64 (d, *J* = 2.4 Hz, 1H), 7.43 (d, *J* = 8.5 Hz, 1H), 7.37 (dd, *J* = 8.5, 2.4 Hz, 1H), 5.94 (s, 1H), 3.51 – 3.42 (m, 2H), 1.70 (m, 1H), 1.56 – 1.46 (m, 2H), 0.96 ppm (d, J = 6.6 Hz, 6H); **^{13}C NMR** (101 MHz, $CDCl_3$) δ 166.12, 139.78, 134.88, 134.25, 132.63, 121.69, 118.01, 38.69, 38.34, 26.05, 22.57 ppm; **ESI HR-MS**: calcd. for [$C_{12}H_{15}Br_2NO+H]^+$: 347.9593, found 347.9596.

***N*-Benzyl-2-bromobenzamide (22)[5]**

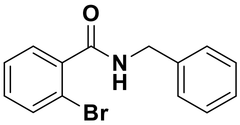

Application of General Procedure B with 2-bromobenzoyl chloride (219 mg, 1 mmol), triethylamine (167 µL, 1.2 mmol), and benzylamine (120 µl, 1.1 mmol) yielded 184 mg (63%) of **22** as a beige solid.

Mp 115-116 °C; **IR (neat):** ν (cm^{-1}) 3065, 2923, 1639, 1539, 1360. **^{1}H NMR** (400 MHz, $CDCl_3$) δ 7.49 (m, 2H), 7.41 – 7.09 (m, 8H), 6.20 (s, 1H), 4.58 ppm (d, *J* = 5.5 Hz, 2H); **^{13}C NMR** (101 MHz, $CDCl_3$) δ 167.59, 137.77, 137.61, 133.54, 131.45, 129.79, 128.91, 128.15, 127.84, 127.70, 119.44, 44.39 ppm; **ESI HR-MS**: calcd. for [$C_{14}H_{12}BrNO+H]^+$: 290.0715, found 290.0717. The spectroscopic data match those reported in the literature.[5]

***N*-(Cyclohexylmethyl)-2-bromobenzamide (23)**

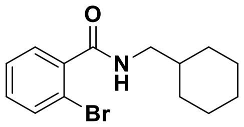

Application of General Procedure B with 2-bromobenzoyl chloride (219 mg, 1 mmol), triethylamine (167 µL, 1.2 mmol), and cyclohexylmethylamine (143 µL, 1.1 mmol) yielded 180 mg (61%) of **23** as a yellow oil.

IR (neat): ν (cm^{-1}) 3068, 2921, 2850, 1641, 1541, 1367; **^{1}H NMR** (400 MHz, $CDCl_3$) δ 7.62 – 7.49 (m, 2H), 7.34 (td, J = 7.5, 1.0 Hz, 1H), 7.30 – 7.21 (m, 2H), 6.03 (s, 1H), 3.30 (dd, *J* = 6.5, 6.0 Hz, 2H), 2.01 – 1.52 (m, 6H), 1.42 – 1.11 (m, 4H), 1.02 ppm (m, 3H); **^{13}C NMR** (101 MHz, $CDCl_3$) δ 167.73, 138.30, 133.47, 131.25, 129.81, 127.68, 119.31, 46.49, 38.00, 31.07, 26.51, 25.96 ppm; **ESI HR-MS**: calcd. for $[C_{14}H_{18}BrNO+H]^+$: 296.0645, found 296.0646.

2-Iodo-*N*-pentylbenzamide (24)[6]

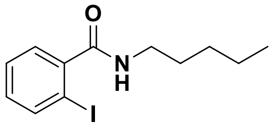

Application of General Procedure B with 2-iodobenzoyl chloride (267 mg, 1 mmol), triethylamine (167 µL, 1.2 mmol), and pentylamine (128 µL, 1.1 mmol) yielded 203 mg (64%) of **24** as a white solid.

Mp 98-100 °C; **IR (neat):** ν (cm^{-1}) 3066, 2926, 1634, 1546; **^{1}H NMR** (400 MHz, $CDCl_3$) δ 7.88 – 7.81 (m, 1H), 7.43 – 7.32 (m, 2H), 7.08 (ddd, *J* = 8.0, 6.5, 3.0 Hz, 1H), 5.78 (s, 1H), 3.44 (td, *J* = 7.0, 6.0 Hz, 2H), 1.70 – 1.56 (m, 2H), 1.46 – 1.30 (m, 5H), 1.30 – 1.17 (m, 3H), 0.99 – 0.82 (m, 4H) ppm; **^{13}C NMR** (101 MHz, $CDCl_3$) δ 169.34, 142.59, 139.83, 130.99, 128.27, 128.16, 92.42, 40.12, 29.16, 29.09, 22.36, 14.01 ppm; **ESI HR-MS**: calcd. for $[C_{12}H_{16}INO+H]^+$: 318.0349, found 318.0349. The spectroscopic data match those reported in the literature.[6]

2-Iodo-*N*-(pyridin-2-yl)benzamide (25)[7]

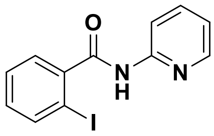

A modified General Procedure B was followed using triethylamine (502 µL, 3.6 mmol), 2-iodobenzoyl chloride (800 mg, 3 mmol) and 2-aminopyridine (310 mg, 3.3 mmol). The reaction mixture was stirred at room temperature for 5 minutes, then diluted in EtOAc and washed with HCl (1 M). The aqueous layer was neutralised using NaOH

(12 M), extracted with EtOAc, and dried over Na_2SO_4. Purification yielded 130 mg (13%) of **25** as a white solid.

Mp 176-177 °C; **IR (neat):** ν (cm^{-1}) 3077, 2956, 1680, 1533, 1434, 1309; **^{1}H NMR** (400 MHz, $CDCl_3$) δ 9.46 (s, 1H), 8.40 (d, *J* = 8.4 Hz, 1H), 7.90 (dd, *J* = 8.0, 1.1 Hz, 1H), 7.85 (dd, *J* = 5.2, 1.9 Hz, 1H), 7.76 (ddd, *J* = 8.8, 7.4, 1.9 Hz, 1H), 7.52 (dd, *J* = 7.6, 1.7 Hz, 1H), 7.41 (td, *J* = 7.5, 1.1 Hz, 1H), 7.15 (td, *J* = 7.7, 1.7 Hz, 1H), 6.99 ppm (ddd, *J* = 7.4, 5.0, 1.0 Hz, 1H); **^{13}C NMR** (101 MHz, $CDCl_3$) δ 167.85, 151.44, 147.31, 141.78, 140.33, 139.06, 131.78, 128.51, 128.45, 120.23, 114.80, 92.65 ppm; **ESI HR-MS**: calcd. for $[C_{12}H_9IN_2O+H]^+$: 324.9843, found 324.9831. The spectroscopic data match those found in the literature.[7]

2-Iodo-*N*-([(3*S*)-5-oxopyrrolidin-3-yl]methyl)benzamide (26)

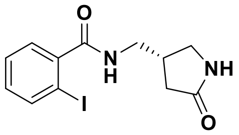

General Procedure B was followed using triethylamine (73.3 µL, 0.53 mmol, 1.3 equiv), 2-iodobenzoyl chloride (106 mg, 0.4 mmol) and (4*R*)-4-(aminomethyl)pyrrolidin-2-one (50 mg, 0.44 mmol, 1.1 equiv). The mixture was stirred for 36 h. The reaction mixture was concentrated *in vacuo,* then purified with flash chromatography. A solvent system of (0→20% CH_2Cl_2 – MeOH gradient) over 15 column volumes was run to yield 45 mg (33%) of **26** as a beige solid.

Mp 175-176 °C; **[α]$_D$^{25}** = -8.1 (c = 0.5, DMSO-*d*$_6$); **IR (neat):** ν (cm^{-1}) 3284, 3095, 2998, 2964, 1697, 1640, 1560, 1368, 1046; **^{1}H NMR** (400 MHz, DMSO-*d*$_6$) δ 8.51 (t, *J* = 5.9 Hz, 1H), 7.87 (dd, *J* = 7.9, 1.1 Hz, 1H), 7.52 (s, 1H), 7.44 (td, *J* = 7.5, 1.1 Hz, 1H), 7.31 (dd, *J* = 7.6, 1.7 Hz, 1H), 7.16 (td, *J* = 7.6, 1.7 Hz, 1H), 3.42 – 3.29 (m, 2H), 3.29 – 3.20 (m, 2H), 3.10 – 3.03 (m, 1H), 2.63 (ddd, *J* = 12.1, 9.5, 6.0 Hz, 1H), 2.29 (dd, *J* = 16.7, 8.9 Hz, 1H), 2.00 ppm (dd, *J* = 16.7, 6.4 Hz, 1H); **^{13}C NMR** (101 MHz, DMSO-*d*$_6$) δ 176.08, 169.20, 143.26, 138.96, 130.65, 127.99, 127.88, 93.40, 44.97, 42.34, 34.38, 33.88 ppm; **ESI HR-MS**: calcd. for $[C_{12}H_{13}IN_2O_2+H]^+$: 345.0094, found 345.0095.

2-Iodo-*N*-(2-methoxyethyl)benzamide (27)

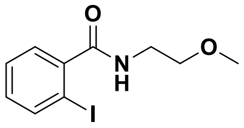

Application of General Procedure B with 2-iodobenzoyl chloride (267 mg, 1 mmol), triethylamine (167 µL, 1.2 mmol), and 2-methoxyethan-1-amine (96 µL, 1.1 mmol) yielded 105 mg (34%) of **27** as a white wax.

IR (neat): ν (cm^{-1}) 3058, 2924, 1647, 1537, 1360, 1120, 1014. **^{1}H NMR** (400 MHz, $CDCl_3$) δ 7.92 – 7.83 (m, 1H), 7.45 – 7.33 (m, 2H), 7.09 (m, 1H), 6.16 (s, 1H), 3.70 – 3.62 (m, 2H), 3.59 (m, 2H), 3.39 ppm (s, 3H); **^{13}C NMR** (101 MHz, $CDCl_3$) δ 169.54, 142.41, 140.00, 131.23, 128.39, 128.30, 92.58, 71.05, 58.94, 39.84 ppm; **ESI HR-MS**: calcd. for $[C_{10}H_{12}INO_2+Na]^+$: 327.9805, found 327.9804.

Methyl 3-[(2-iodophenyl)formamido]propanoate (28)[8]

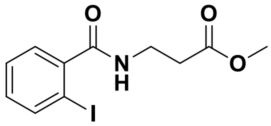

Application of General Procedure B using triethylamine (169 µL, 1.2 mmol, 1.2 equiv), 2-iodobenzoyl chloride (267 mg, 1 mmol), and methyl 3-aminopropanoate (153 mg, 1.1 mmol) yielded 197 mg (59%) of **28** as a white solid.

Mp 89-91 °C; **IR (neat):** ν (cm^{-1}) 3031, 2949, 2919, 1731, 1635, 1537, 1330, 1206, 1053; **^{1}H NMR** (400 MHz, $CDCl_3$) δ 7.84 (d, J = 8.0 Hz, 1H), 7.36 (m, 2H), 7.14 – 7.03 (m, 1H), 6.38 (s, 1H), 3.77 – 3.68 (m, 6H), 2.70 ppm (t, J = 5.9 Hz, 2H); **^{13}C NMR** (101 MHz, $CDCl_3$) δ 173.18, 169.54, 142.21, 139.95, 131.26, 128.32, 128.27, 92.51, 52.08, 35.46, 33.73 ppm; **ESI HR-MS**: calcd. for $[C_{11}H_{12}INO_3+H]^+$: 333.9935, found 333.9935. The spectroscopic data match those reported in the literature.[8]

Methyl 2-[(2-iodophenyl)formamido]acetate (29)[9]

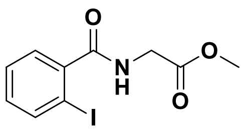

Application of General Procedure B with 2-iodobenzoyl chloride (267 mg, 1 mmol), triethylamine (167 µL, 1.2 mmol), and methyl 2-aminoacetate hydrochloride (138 mg, 1.1 mmol) yielded 231 mg (71%) of **29** as a white solid.

Mp 96-98 °C; **IR (neat):** ν (cm^{-1}) 3058, 2920, 1749, 1654, 1532, 1211, 1017; **^{1}H NMR** (400 MHz, $CDCl_3$) δ 7.88 (dd, *J* = 7.9, 1.1 Hz, 1H), 7.44 (dd, *J* = 7.6, 1.9 Hz, 1H), 7.38 (td, *J* = 7.5, 1.1 Hz, 1H), 7.11 (ddd, *J* = 8.0, 7.3, 1.9 Hz, 1H), 6.37 (s, 1H), 4.25 (d, *J* = 5.1 Hz, 2H), 3.80 ppm (s, 3H); **^{13}C NMR** (101 MHz, $CDCl_3$) δ 170.03, 169.16, 141.33, 140.08, 131.43, 128.46, 128.18, 92.38, 52.52, 41.69 ppm; **ESI HR-MS**: calcd. for $[C_{10}H_{10}INO_3+Na]^+$: 341.9598, found 341.9598. The spectroscopic data match those reported in the literature.[9]

General procedure C – Sulfur insertion and ring closure reaction to give 3a-c, 3e-f, 4a-d, 5-11

Following a modified literature procedure,[10] a reaction vial was charged with CuI (0.3 equiv.) and 1,10-phenanthroline (0.3 equiv). The atmosphere was purged with argon and anhydrous DMF (0.4 M) was added. The suspension was stirred at rt for 15 min, then the appropriate benzamide (1 equiv), S_8 (1.3 equiv), and K_2CO_3 (1.3 equiv) were added. The suspension was stirred for a further 15 min at rt, then heated to 110°C until TLC showed full conversion of the starting material (TLC, MS). The reaction was cooled to rt and diluted in brine. The resulting mixture was extracted with EtOAc. The combined organic phases were dried over Na_2SO_4, then concentrated *in vacuo*. Purification by flash chromatography yielded the desired compound.

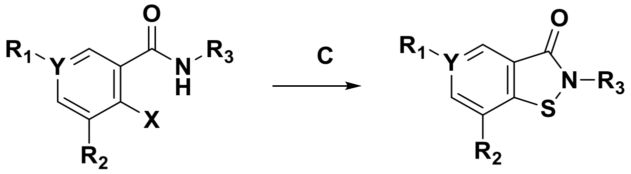

Figure S6. Sulfur insertion and ring closure (3a-c, 3e-f, 4a-d, 5-11). Conditions; C: 1,10-phenanthroline/CuI, S_8, K_2CO_3, DMF, 110 °C.

2-Phenyl-2,3-dihydro-1,2-benzothiazol-3-one (3a)[10]

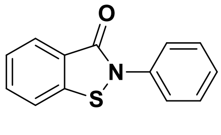

Application of General Procedure C using CuI (29 mg, 0.15 mmol), 1,10-phenanthroline (27 mg, 0.15 mmol), 2-iodo-*N*-phenylbenzamide (**13**) (239 mg, 0.74 mmol), S_8 (22 mg, 0.65 mmol), and K_2CO_3 (89 mg, 0.65 mmol) yielded 117 mg (70%) of **3a** as a white solid.

Mp 140-142 °C; **IR (neat):** ν (cm^{-1}) 3064, 2921, 1663, 1593, 1488, 1448, 1299; **^{1}H NMR** (400 MHz, $CDCl_3$) δ 8.11 (dd, *J* = 7.9, 1.2, 1H), 7.76 – 7.69 (m, 2H), 7.67 (ddd, *J* = 8.2, 7.1, 1.2 Hz, 1H), 7.59 (dt, *J* = 8.1, 0.9 Hz, 1H), 7.52 – 7.41 (m, 3H), 7.37 – 7.28 ppm (m, 1H); **^{13}C NMR** (101 MHz, $CDCl_3$) δ 164.26, 140.02, 137.40, 132.48, 129.50, 127.35, 127.19, 125.94, 125.01, 124.73, 120.22 ppm; **ESI HR-MS**: calcd. for $[C_{13}H_9NOS+H]^+$: 228.0480, found: 228.0478. The spectroscopic data correspond to those reported in the literature.[10]

2-Phenyl-2,3-dihydro-1,2-benzothiazol-3-one (3b)

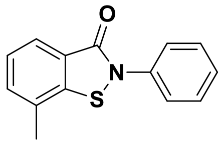

Application of General Procedure C using CuI (23 mg, 0.12 mmol) and 1,10-phenanthroline (22 mg, 0.12 mmol), 2-iodo-3-methyl-*N*-phenylbenzamide (**14**) (136 mg, 0.4 mmol), S_8 (17 mg, 0.52 mmol), and K_2CO_3 (72 mg, 0.52 mmol) yielded 7 mg (7%) of **3b** as a beige solid.

Mp 113-115 °C; **IR (neat):** ν (cm^{-1}) 2999, 2956, 2917, 1665, 1541, 1377, 1133, 1024; **^{1}H NMR** (400 MHz, $CDCl_3$) δ 7.95 (ddd, *J* = 7.6, 1.3, 0.7 Hz, 1H), 7.78 – 7.69 (m, 2H), 7.51 – 7.42 (m, 3H), 7.39 (t, *J* = 7.5 Hz, 1H), 7.35 – 7.29 (m, 1H), 2.39 ppm (d, *J* = 0.8 Hz, 3H); **^{13}C NMR** (101 MHz, $CDCl_3$) δ 164.91, 140.11, 137.61, 132.65, 129.83, 129.49, 127.14, 126.49, 124.91, 124.75, 18.75 ppm; **ESI HR-MS**: calcd. for $[C_{14}H_{11}NOS+H]^+$: 242.0634, found: 242.0636.

7-Bromo-2-phenyl-2,3-dihydro-1,2-benzothiazol-3-one (3c)

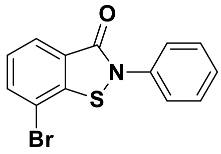

Application of General Procedure C using CuI (27 mg, 0.14 mmol) and 1,10-phenanthroline (26 mg, 0.14 mmol), 2,3-dibromo-*N*-phenylbenzamide (**15**) (169 mg, 0.48 mmol), S_8 (21 mg, 0.62 mmol), and K_2CO_3 (85 mg, 0.62 mmol) yielded 76 mg (52%) of **3c** as an orange solid.

Mp 149-150 °C; **IR (neat):** ν (cm^{-1}) 2961, 2918, 2849, 1669, 1488, 1403, 1298, 1072; **^{1}H NMR** (400 MHz, $CDCl_3$) δ 8.09 (dd, *J* = 7.8, 1.0 Hz, 1H), 7.78 (dd, *J* = 7.8, 1.0 Hz, 1H), 7.74 – 7.68 (m, 2H), 7.53 – 7.46 (m, 2H), 7.41 – 7.31 ppm (m, 2H); **^{13}C NMR** (101 MHz, $CDCl_3$) δ 164.06,

142.42, 137.07, 134.75, 129.62, 127.55, 127.51, 127.21, 126.09, 124.87, 113.97 ppm; **ESI HR-MS**: calcd. for $[C_{13}H_8BrNOS+H]^+$: 305.9583, found: 305.9583.

5-Bromo-2-phenyl-2,3-dihydro-1,2-benzothiazol-3-one (3e)

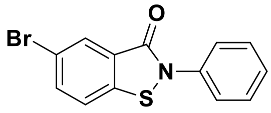

Application of General Procedure C using CuI (89 mg, 0.47 mmol), 1,10-phenanthroline (84 mg, 0.47 mmol), 2,5-dibromo-*N*-phenylbenzamide (**16**) (553 mg, 1.56 mmol), S_8 (67 mg, 2.03 mmol), and K_2CO_3 (280 mg, 2.03 mmol) yielded 412 mg (86%) of **3e** as a beige solid.

Mp 188-189 °C; **IR (neat):** ν (cm^{-1}) 3092, 2981, 2884, 1642, 1493, 1444, 1337; **^{1}H NMR** (400 MHz, $CDCl_3$) δ 8.24 (d, J = 2.0 Hz, 1H), 7.76 (dd, J = 8.5, 2.0 Hz, 1H), 7.68 (dd, *J* = 7.7, 1.6 Hz, 2H), 7.48 (t, *J* = 7.5 Hz, 3H), 7.34 ppm (t, *J* = 7.4 Hz, 1H); **^{13}C NMR** (101 MHz, $CDCl_3$) δ 162.93, 138.69, 137.02, 135.57, 130.13, 129.61, 127.56, 124.78, 121.71, 119.79 ppm; **ESI HR-MS**: calcd. for $[C_{13}H_8BrNOS+H]^+$: 305.9582, found 305.9584.

2-Phenyl-2*H*,3*H*-[1,2]thiazolo[4,5-*c*]pyridin-3-one (3f)

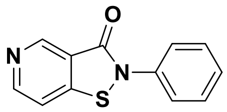

General Procedure C was followed using CuI (5.7 mg, 0.03 mmol), 1,10-phenanthroline (5.7 mg, 0.03 mmol), 4-chloro-N-phenylpyridine-3-carboxamide (**25**) (38.8 mg, 0.11 mmol), S8 (4.8 mg, 0.14 mmol), and K_2CO_3 (19.7 mg, 0.14 mmol). Purification by flash chromatography (0 to 45%, v/v EtOAc/cyclohexane) yielded 15 mg (58%) of **3f** as a yellow solid.

Mp 145-147 °C; **IR (neat):** ν (cm^{-1}) 2922, 2846, 1670, 1584, 1440, 1287, 1031; **^{1}H NMR** (400 MHz, $CDCl_3$) δ 9.29 (s, 1H), 8.75 (d, *J* = 5.4 Hz, 1H), 7.71 – 7.63 (m, 2H), 7.60 (d, *J* = 5.4 Hz, 1H), 7.56 – 7.45 (m, 2H), 7.44 – 7.33 ppm (m, 1H). **^{13}C NMR** (151 MHz, $CDCl_3$) δ 162.84, 149.87, 149.27, 149.06, 136.40, 129.77, 127.96, 125.00, 120.97, 115.24 ppm. **ESI HR-MS**: calcd. for $[C_{12}H_8N_2OS+H]^+$: 229.0430, found: 229.0432.

2-(3-Methylbutyl)-2,3-dihydro-1,2-benzothiazol-3-one (4a)[11]

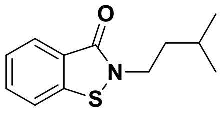

Application of General Procedure C using CuI (28.9 mg, 0.15 mmol), 1,10-phenanthroline (27.4 mg, 0.15 mmol), 2-iodo-*N*-(3-methylbutyl)benzamide (**18**) (160 mg, 0.51 mmol), S_8 (21.9 mg, 0.66 mmol), and K_2CO_3 (90.7 mg, 0.66 mmol) yielded 32 mg (28%) of **4a** as an orange solid.

Mp 128-129 °C; **IR (neat):** ν (cm^{-1}) 3075, 2956, 2583, 1647, 1597, 1442, 1347, 1291, 1072; **^{1}H NMR** (400 MHz, $CDCl_3$) δ 8.03 (dt, *J* = 7.9, 1.0 Hz, 1H), 7.65 – 7.51 (m, 2H), 7.39 (ddd, *J* = 8.0, 6.9, 1.2 Hz, 1H), 4.00 – 3.83 (m, 2H), 1.69 – 1.62 (m, 4H), 0.98 ppm (d, *J* = 6.3 Hz, 6H).; **^{13}C NMR** (101 MHz, $CDCl_3$) δ 165.40, 140.28, 131.74, 126.79, 125.55, 125.05, 120.43, 42.47, 38.46, 25.79, 22.55 ppm; **ESI HR-MS**: calcd. for $[C_{12}H_{15}NOS+H]^+$: 222.0947, found: 222.0950. The spectroscopic date match those reported in the literature.[11]

7-Methyl-2-(3-methylbutyl)-2,3-dihydro-1,2-benzothiazol-3-one (4b)

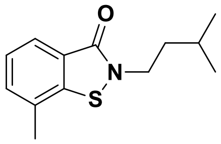

General Procedure C was followed using CuI (37 mg, 0.2 mmol), 1,10-phenanthroline (35 mg, 0.2 mmol), 2-iodo-3-methyl-*N*-(3-methylbutyl)benzamide (**19**) (215 mg, 0.65 mmol), S_8 (28.2 mg, 0.85 mmol), and K_2CO_3 (117 mg, 0.85 mmol). Purification by flash chromatography (0 to 40%, v/v EtOAc/cyclohexane) yielded 94 mg (61%) of **4b** as a colourless oil.

IR (neat): ν (cm^{-1}) 3066, 2957, 1662, 1478, 1385, 1211; **^{1}H NMR** (400 MHz, $CDCl_3$) δ 7.91 – 7.84 (m, 1H), 7.41 – 7.30 (m, 2H), 3.96 – 3.90 (m, 2H), 2.36 (s, 3H), 1.67 (m, 3H), 1.03 – 0.93 ppm (m, 6H); **^{13}C NMR** (101 MHz, $CDCl_3$) δ 166.05, 140.38, 131.88, 129.95, 126.07, 124.89, 124.18, 42.54, 38.51, 25.77, 22.55, 18.80 ppm; **ESI HR-MS**: calcd. for $[C_{13}H_{17}NOS+H]^+$: 236.1104, found: 236.1105.

5-Bromo-2-(3-methylbutyl)-2,3-dihydro-1,2-benzothiazol-3-one (4c)

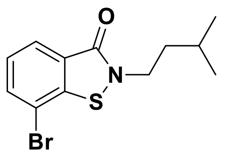

General Procedure C was followed using CuI (21.8 mg, 0.12 mmol), 1,10-phenanthroline (20.7 mg, 0.12 mmol), 2,3-dibromo-*N*-(3-methylbutyl)benzamide (**20**) (133.5 mg, 0.38 mmol), S_8 (16.6 mg, 0.5 mmol), and K_2CO_3 (68.6 mg, 0.5 mmol). Purification by reverse-phase chromatography using a Biotage Sfär® C18 Duo cartridge (buffer A (H_2O:formic acid, 100:0.1 v/v) to buffer B (MeCN:formic acid, 100:0.1 v/v) from 0 to 100% B over 16 column volumes, then 4 column volumes at 100% B) gave 33 mg (29%) of **4c** as an orange oil.

IR (neat): ν (cm^{-1}) 2962, 2917, 2849, 1669, 1488, 1328. 1089; **^{1}H NMR** (400 MHz, DMSO-*d_6*) δ 7.97 (dd, *J* = 7.7, 1.0 Hz, 1H), 7.94 (dd, *J* = 7.8, 1.0 Hz, 1H), 7.46 (t, *J* = 7.8 Hz, 1H), 3.90 (t, *J* = 7.0 Hz, 2H), 1.64 – 1.48 (m, 3H), 0.97 – 0.87 ppm (m, 6H); **^{13}C NMR** (101 MHz, DMSO-*d_6*) δ 164.12, 141.60, 134.40, 127.87, 126.35, 125.11, 113.70, 41.63, 37.68, 24.97, 22.18 ppm; **ESI HR-MS**: calcd. for $[C_{12}H_{14}BrNOS+H]^+$: 300.0052, found: 300.0054.

7-Bromo-2-(3-methylbutyl)-2,3-dihydro-1,2-benzothiazol-3-one (4d)

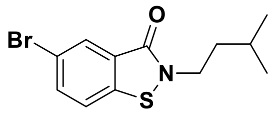

General Procedure C was followed using CuI (25.8 mg, 0.14 mmol), 1,10-phenanthroline (24.5 mg, 0.14 mmol), 2,5-dibromo-*N*-(3-methylbutyl)benzamide (**21**) (157.7 mg, 0.45 mmol), S_8 (19.6 mg, 0.59 mmol), and K_2CO_3 (81.1 mg, 0.59 mmol). Purification by flash chromatography (0 to 40%, v/v EtOAc/cyclohexane) yielded 20 mg (15%) of **4d** as a beige solid.

Mp 107-108 °C; **IR (neat):** ν (cm^{-1}) 3093, 2947, 2849, 1650, 1453, 1308, 1078; **^{1}H NMR** (400 MHz, $CDCl_3$) δ 8.16 (d, *J* = 1.9 Hz, 1H), 7.69 (dd, *J* = 8.5, 2.0 Hz, 1H), 7.42 (d, *J* = 8.4 Hz, 1H), 3.94 – 3.86 (m, 2H), 1.65 (m, 3H), 0.97 (d, *J* = 6.3 Hz, 6H); **^{13}C NMR** (101 MHz, $CDCl_3$) δ 164.07, 138.91, 134.83, 129.61, 126.75, 121.89, 119.41, 42.70, 38.39, 29.84, 25.79, 22.51 ppm; **ESI HR-MS**: calcd. for $[C_{12}H_{14}BrNOS+H]^+$: 300.0052, found 300.0053.

2-Benzyl-2,3-dihydro-1,2-benzothiazol-3-one (5)[10]

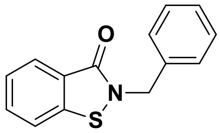

Application of General Procedure C using CuI (29 mg, 0.15 mmol), 1,10-phenanthroline (27 mg, 0.15 mmol), *N*-benzyl-2-bromobenzamide (**22**) (145 mg, 0.5 mmol), S_8 (22 mg, 0.65 mmol), and K_2CO_3 (89 mg, 0.65 mmol) yielded 59 mg (49%) of **5** as a beige solid.

Mp 140-142 °C; **IR (neat):** ν (cm^{-1}) 3063, 2920, 1654, 1447, 1333; **^{1}H NMR** (400 MHz, $CDCl_3$) δ 8.07 (dt, *J* = 7.9, 1.0 Hz, 1H), 7.59 (ddd, *J* = 8.3, 7.1, 1.3 Hz, 1H), 7.49 (dt, *J* = 8.2, 0.9 Hz, 1H), 7.40 (ddd, *J* = 8.0, 7.1, 1.0 Hz, 1H), 7.37 – 7.29 (m, 5H), 5.06 ppm (s, 2H); **^{13}C NMR** (101 MHz, $CDCl_3$) δ 165.50, 140.55, 136.33, 131.98, 128.98, 128.59, 128.43, 126.98, 125.65, 124.65, 120.53, 47.73 ppm; **ESI HR-MS**: calcd. for $[C_{14}H_{11}NOS+Na]^+$: 264.0454, found: 264.0454. The spectroscopic data correspond to those reported in the literature.[10]

2-(Cyclohexylmethyl)-2,3-dihydro-1,2-benzothiazol-3-one (6)

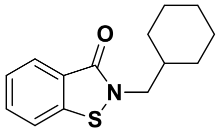

Application of General Procedure C using CuI (29 mg, 0.15 mmol), 1,10-phenanthroline (27 mg, 0.15 mmol), *N*-(Cyclohexylmethyl)-2-bromobenzamide (**23**) (129 mg, 0.44 mmol), S_8 (22 mg, 0.65 mmol), and K_2CO_3 (89 mg, 0.65 mmol) yielded 22 mg (20%) of **6** as a colourless oil.

IR (neat): ν (cm^{-1}) 3065, 2922, 1652, 1447, 1330; **^{1}H NMR** (400 MHz, $CDCl_3$) δ 8.03 (dt, *J* = 7.9, 1.0 Hz, 1H), 7.64 – 7.50 (m, 2H), 7.39 (ddd, *J* = 8.0, 7.0, 1.2 Hz, 1H), 3.73 (d, *J* = 7.3 Hz, 2H), 1.88 – 1.61 (m, 6H), 1.29 – 1.14 (m, 3H), 1.11 – 0.99 ppm (m, 2H); **^{13}C NMR** (101 MHz, $CDCl_3$) δ 165.71, 140.35, 131.75, 126.87, 125.51, 124.89, 120.33, 50.17, 38.30, 30.68, 26.41, 25.79 ppm; **ESI HR-MS**: calcd. for $[C_{14}H_{17}NOS+H]^+$: 248.1104, found: 248.1104.

2-Pentyl-2,3-dihydro-1,2-benzothiazol-3-one (7)[11]

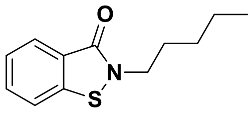

Application of General Procedure C using CuI (32 mg, 0.18 mmol), 1,10-phenanthroline (34 mg, 0.18 mmol), 2-iodo-*N*-pentylbenzamide (**24**)

(190 mg, 0.6 mmol), S_8 (26 mg, 0.78 mmol), and K_2CO_3 (108 mg, 0.78 mmol) yielded 108 mg (81%) of **7** as a colourless oil.

IR (neat): ν (cm^{-1}) 3066, 2955, 1650, 1446, 1336; **^{1}H NMR** (400 MHz, $CDCl_3$) δ 8.03 (dt, *J* = 7.9, 1.0 Hz, 1H), 7.64 – 7.50 (m, 2H), 7.39 (ddd, *J* = 8.0, 6.9, 1.2 Hz, 1H), 3.93 – 3.85 (m, 2H), 1.82 – 1.71 (m, 2H), 1.40 – 1.33 (m, 4H), 0.94 – 0.87 ppm (m, 3H); **^{13}C NMR** (101 MHz, $CDCl_3$) δ 165.44, 140.29, 131.74, 126.80, 125.54, 125.03, 120.43, 44.08, 29.37, 28.84, 22.41, 14.05 ppm; **ESI HR-MS**: calcd. for $[C_{12}H_{15}NOS+H]^+$: 222.0947, found: 222.0949. The spectroscopic data correspond to those reported in the literature.[11]

2-(Pyridin-2-yl)benzo[*d*]isothiazol-3(2*H*)-one (8)[12]

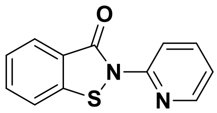

General Procedure C was followed using CuI (21.47 mg, 0.11 mmol), 1,10-phenanthroline (20.34 mg, 0.11 mmol), 2-Iodo-*N*-(pyridin-2-yl)benzamide (**25**) (123 mg, 0.38 mmol), S_8 (16.5 mg, 0.49 mmol), and K_2CO_3 (68.1 mg, 0.49 mmol). The reaction mixture diuted with HCl (1M) and extracted with EtOAc. Purification by flash chromatography (0 to 45%, v/v EtOAc/cyclohexane) yielded 57 mg (65%) mg of **8** as a pink solid.

Mp 191-192 °C; **IR (neat):** ν (cm^{-1}) 3070, 2917, 2849, 1672, 1583, 1450, 1259, 1119; **^{1}H NMR** (400 MHz, $CDCl_3$) δ 8.75 (dt, *J* = 8.5, 1.0 Hz, 1H), 8.41 (ddd, *J* = 5.0, 1.8, 0.9 Hz, 1H), 8.06 (dt, *J* = 7.9, 1.0 Hz, 1H), 7.81 (ddd, *J* = 8.5, 7.3, 1.8 Hz, 1H), 7.65 (ddd, *J* = 8.2, 7.1, 1.2 Hz, 1H), 7.58 (dt, *J* = 8.1, 1.0 Hz, 1H), 7.40 (ddd, *J* = 8.1, 7.1, 1.1 Hz, 1H), 7.14 ppm (ddd, *J* = 7.3, 4.9, 1.0 Hz, 1H); **13C NMR** (101 MHz, $CDCl_3$) δ 164.19, 150.56, 147.75, 141.16, 138.55, 132.96, 126.95, 126.76, 125.66, 120.83, 120.45, 114.62 ppm; **ESI HR-MS**: calcd. for $[C_{12}H_8N_2OS+H]^+$: 229.0430, found 229.0433. The spectroscopic data match those reported in the literature.[12]

(*S*)-2-((5-Oxopyrrolidin-3-yl)methyl)benzo[*d*]isothiazol-3(2*H*)-one (9)

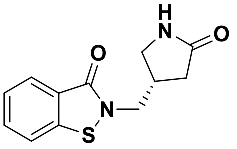

General Procedure C was followed with CuI (24.8 mg, 0.03 mmol), 1,10-phenanthroline (23.5 mg, 0.03 mmol), 2-Iodo-*N*-([(3*S*)-5-oxopyrrolidin-3-yl]methyl)benzamide (**26**) (34.6 mg, 0.1 mmol), S_8 (4.4 mg, 0.13 mmol), and K_2CO_3 (18 mg, 0.13 mmol). The reaction mixture was filtered through cotton wool and purified using flash chromatography (0→20% MeOH in CH_2Cl_2) to give 4 mg (17%) of **9** as an orange wax.

[α]$_D^{25}$ = +8.8 (c = 1, $CDCl_3$); **IR (neat):** ν (cm^{-1}) 3276, 2927, 1685, 1645, 1447, 1104; **^{1}H NMR** (400 MHz, $CDCl_3$) δ 8.04 (dt, *J* = 7.9, 1.0 Hz, 1H), 7.64 (ddd, *J* = 8.2, 7.0, 1.2 Hz, 1H), 7.56 (dt, *J* = 8.1, 0.9 Hz, 1H), 7.43 (ddd, *J* = 8.0, 7.1, 1.0 Hz, 1H), 5.89 (s, 1H), 4.09 (dd, *J* = 14.2, 8.2 Hz, 1H), 3.87 (dd, *J* = 14.2, 6.5 Hz, 1H), 3.52 (dd, *J* = 9.9, 7.7 Hz, 1H), 3.32 (dd, *J* = 10.0, 5.6 Hz, 1H), 3.11 – 2.97 (m, 1H), 2.53 (dd, J = 17.1, 8.8 Hz, 1H), 2.24 ppm (dd, *J* = 17.1, 6.8 Hz, 1H); **^{13}C NMR** (101 MHz, $CDCl_3$) δ 176.99, 165.87, 140.22, 132.30, 126.97, 125.94, 124.25, 120.53, 47.15, 45.60, 35.12, 34.68 ppm; **ESI HR-MS**: calcd. for $[C_{12}H_{12}N_2O_2S+H]^+$ 249.0692, found 249.0692. The spectroscopic data match those reported in the literature.[5]

2-(2-Methoxyethyl)-2,3-dihydro-1,2-benzothiazol-3-one (10)[13]

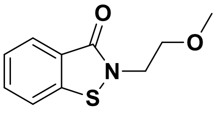

Application of General Procedure C using CuI (17 mg, 0.09 mmol), 1,10-phenanthroline (16 mg, 0.09 mmol), 2-iodo-*N*-(2-methoxy-ethyl)benzamide (**27**) (95 mg, 0.31 mmol), S_8 (13 mg, 0.4 mmol), and K_2CO_3 (55 mg, 0.4 mmol) yielded 40 mg (62%) of **10** as a white solid.

Mp 136-138 °C; **IR (neat):** ν (cm^{-1}) 2961, 2923, 2852, 1653, 1558, 1541, 1458, 1260, 1090; **^{1}H NMR** (400 MHz, $CDCl_3$) δ 8.03 (dt, *J* = 7.9, 1.0 Hz, 1H), 7.59 (ddd, *J* = 8.2, 7.0, 1.3 Hz, 1H), 7.55 – 7.51 (m, 1H), 7.38 (ddd, *J* = 8.0, 7.0, 1.1 Hz, 1H), 4.09 (dd, *J* = 5.5, 4.7 Hz, 2H), 3.69 (dd, *J* = 5.5, 4.7 Hz, 2H), 3.40 ppm (s, 3H) **^{13}C NMR** (101 MHz, $CDCl_3$) δ 165.67, 141.23, 131.86, 126.74, 125.44, 124.34, 120.28, 71.25, 59.04, 43.96 ppm; **ESI HR-MS**: calcd. for

$[C_{10}H_{11}NO_2S+H]^+$: 210.0583 found 210.0584. The spectroscopic data is consistent with those reported in the literature.[13]

Methyl 3-(3-oxo-2,3-dihydro-1,2-benzothiazol-2-yl)propanoate (11)[14]

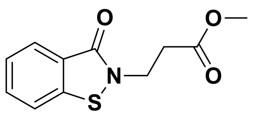

Application of General Procedure C using CuI (32.1 mg, 0.17 mmol), 1,10-phenanthroline (30.6 mg, 0.17 mmol), Methyl 3-[(2-iodophenyl)formamido]propanoate (**28**) (188.6 mg, 0.57 mmol), S_8 (24.5 mg, 0.74 mmol), and K_2CO_3 (101.6 mg, 0.74 mmol) yielded 108 mg (80%) of **11** as a pale yellow solid.

Mp 97-98 °C; **IR (neat):** ν (cm^{-1}) 3066, 2951, 2858, 1727, 1649, 1423, 1371, 1176; **^{1}H NMR** (400 MHz, $CDCl_3$) δ 8.02 (dt, *J* = 7.9, 1.1 Hz, 1H), 7.60 (ddd, *J* = 8.3, 7.1, 1.3 Hz, 1H), 7.53 (dt, *J* = 8.1, 0.9 Hz, 1H), 7.39 (ddd, *J* = 8.1, 7.0, 1.0 Hz, 1H), 4.18 (t, *J* = 6.6 Hz, 2H), 3.72 (s, 3H), 2.80 ppm (t, *J* = 6.6 Hz, 2H); **^{13}C NMR** (101 MHz, $CDCl_3$) δ 171.68, 165.56, 140.72, 132.03, 126.75, 125.64, 124.39, 120.44, 52.15, 40.01, 33.97 ppm; **ESI HR-MS**: calcd. for $[C_{11}H_{11}NO_3S+H]^+$: 238.0532 found 238.0534. The spectroscopic data match those reported in the literature.[14]

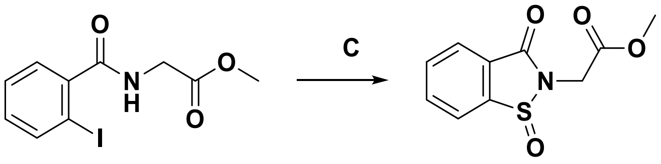

Figure S7. Synthesis of the sulfoxide derivative 12. Conditions; C: 1,10-phenanthroline/CuI, S_8, K_2CO_3, ambient atmosphere, DMF, 110 °C.

Methyl 2-(1-oxido-3-oxobenzo[*d*]isothiazol-2(3*H*)-yl)acetate (12)

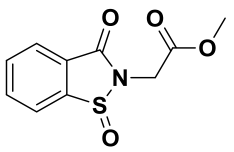

Application of General Procedure C using CuI (36 mg, 0.2 mmol), 1,10-phenanthroline (39 mg, 0.2 mmol), Methyl 2-[(2-iodophenyl)formamido]acetate (**29**) (217 mg, 0.68 mmol), S_8 (29 mg, 0.88 mmol), and K_2CO_3 (122 mg, 0.88 mmol) yielded 25 mg (15%) of **12** as a colourless wax on one occasion.

IR (neat): ν (cm^{-1}) 3058, 2921, 1751, 1649, 1530, 1465, 1209; **^{1}H NMR** (400 MHz, $CDCl_3$) δ 7.64 (dd, *J* = 7.6, 1.6 Hz, 1H), 7.47 – 7.37 (m, 1H), 7.34 (dd, *J* = 8.0, 1.3 Hz, 1H), 7.08 – 6.95 (m, 1H), 4.28 (d, *J* = 5.1 Hz, 2H), 3.80 ppm (s, 3H); **^{13}C NMR** (151 MHz, $CDCl_3$) δ 168.15, 165.76, 140.84, 132.32, 127.03, 125.66, 123.44, 120.40, 52.68, 44.52 ppm. **ESI HR-MS**: calcd. For $[C_{10}H_9NO_4S+H]^+$: 240.0324, found 240.0325.

Methyl 2-(3-oxo-2,3-dihydro-1,2-benzothiazol-2-yl)acetate (12i)[15]

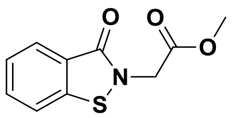

Application of General Procedure C using CuI (36 mg, 0.2 mmol), 1,10-phenanthroline (39 mg, 0.2 mmol), methyl-(2-iodobenzoyl)glycinate (**29**) (217 mg, 0.68 mmol), S_8 (29 mg, 0.88 mmol), and K_2CO_3 (122 mg, 0.88 mmol) yielded 13 mg (29%) of **12** as a colourless oil.

^{1}H NMR (400 MHz, $CDCl_3$) δ 8.05 (d, *J* = 8.0 Hz, 1H), 7.63 (d, *J* = 8.0 Hz, 1H), 7.56 (d, *J* = 7.9 Hz, 1H), 7.43 (d, *J* = 7.9 Hz, 1H), 4.63 (s, 2 H), 3.79 ppm (s, 3H); **^{13}C NMR** (101 MHz, $CDCl_3$) δ 168.38, 166.12, 140.78, 132.67, 127.02, 125.93, 123.51, 120.58, 52.89, 44.78 ppm. **ESI HR-MS**: calcd. For $[C_{10}H_9NO_3S+H]^+$: 224.0376, found 224.0375. The spectroscopic data match those reported in the literature.[15]

General Procedure D – Suzuki-Miyaura cross-coupling reaction

A reaction vial was charged with 7-bromo-2-phenylbenzo[*d*]isothiazol-3(2*H*)-one (52.6 mg, 0.17 mmol, 1 equiv), phenylboronic acid (41.9 mg, 0.34 mmol, 2 equiv), K_2CO_3 (118.5 mg, 0.86 mmol, 5 equiv), and $Pd(dppf)Cl_2$ (18.84 mg, 0.026 mmol, 0.15 equiv). The atmosphere was purged with nitrogen and anhydrous, degassed dioxane (11.6 mL, 0.015 M) was transferred to the reaction vial. The vial was sealed and stirred at 80 °C until full conversion of the starting material (TLC, MS). The reaction mixture was filtered using diatomaceous earth, concentrated

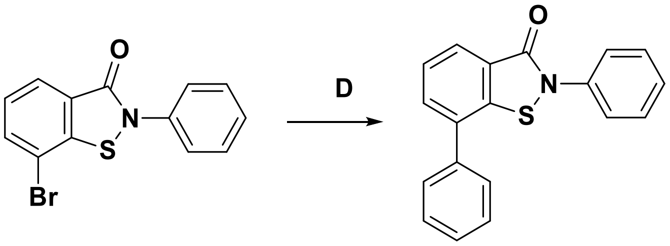

in vacuo, and purified using flash chromatography (0→20% diethyl ether in n-pentane) to yield the desired compound.

Figure S8. Suzuki-Miyaura cross-coupling reaction (3d). Conditions; D: phenylboronic acid, K_2CO_3, $PdCl_2$(dppf), 1,4-dioxane, 80 °C.

2,7-Diphenyl-2,3-dihydro-1,2-benzothiazol-3-one (3d)

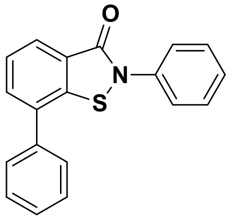

Application of General Procedure D using 7-Bromo-2-phenylbenzo[*d*]isothiazol-3(2*H*)-one (52.6 mg, 0.17 mmol), phenylboronic acid (41.9 mg, 0.34 mmol), and K_2CO_3 (118.5 mg, 0.86 mmol) yielded 10 mg (19%) of **3d** as a white solid.

Mp 153-154 °C; **IR (neat): υ_{max}(cm^{-1})** 3055, 2918, 2850, 1675, 1495, 1327, 1174; **^{1}H NMR** (400 MHz, $CDCl_3$) δ 8.11 (dd, *J* = 7.8, 1.2 Hz, 1H), 7.76 – 7.68 (m, 3H), 7.62 – 7.43 (m, 8H), 7.31 ppm (m, 1H); **^{13}C NMR** (101 MHz, $CDCl_3$) δ 164.57, 139.17, 138.41, 137.41, 134.53, 131.93, 129.64, 129.49, 129.01, 127.24, 127.20, 126.98, 126.14, 126.02, 124.73 ppm; **ESI HR-MS**: calcd. for $[C_{19}H_{13}NOS+H]^+$: 304.0791, found: 304.0792.

Biochemical Assays

General Information

M^{pro} assays were performed exclusively using freshly purified recombinant M^{pro} solution, prepared as reported.[16] Refrozen M^{pro} samples exhibited reduced activity and were not used. LCMS (Merck, Supelco) grade solvents were used for MS analyses and to prepare buffers. The peptide substrate and M^{pro} solution were diluted in freshly prepared reaction buffer (defined below) to prepare stock solutions. Compound stock solutions were prepared to 10mM in DMSO and diluted using the reaction buffer.

Fluoroscence Assays

The inhibitory activities of compounds **1-12** against M^{pro} were investigated using a spectroscopic assay, monitoring M^{pro} catalysed hydrolysis of the fluorogenic substrate ([5-FAM]-AVLQSGFR-[Lys(Dabcyl)]-K-amide) as reported.[17] In brief, assays were conducted at room temperature in clear-bottomed Greiner 384 black well microplates using a ClarioStar or PHERAstar FS microplate reader (BMG LabTech). Compound dilutions were transferred in

quadruplicate from a 96 well plate to a 384 well plate (5 µL/well) using a CyBio Liquid Handler (Analytik Jena AG). M^{pro} (5 µl/well) and substrate (10 µL/well) were dispensed across 384 well plates using a Multidrop Combi dispenser (Thermo Scientific), the plates were then centrifuged (500 g, 15 s, Axygen Plate Spinner Centrifuge, Corning). Final reaction concentrations were 50 nM M^{pro}, 375 nM substrate, 20 mM HEPES, pH 7.3, 50 mM NaCl, 10% glycerol, 1% DMSO, 0.01% v/v Triton X-100. The initial rates of reaction (measured after 15 min pre-incubation of the inhibitor with the enzyme) were assessed by monitoring the fluorescence intensity at λ_{ex} = 485 nm and λ_{em} = 520 nm. Following the determination of initial rates of reaction, data were fitted using a four-parameter function: log (inhibitor) vs. response, variable slope in GraphPad Prism 5 to obtain IC_{50} values.

Solid-Phase Extraction Coupled to MS Assays

The inhibitory activity of compounds **1-12** against M^{pro} was also investigated using a RapidFire (RF) 365 high-throughput sampling robot (Agilent) connected to an iFunnel Agilent 6550 accurate mass quadrupole time-of-flight (Q-TOF) mass spectrometer as reported.[16] In brief, compounds **1-12** were dispensed in an 11-point, 3-fold dilution series (100 µM top concentration) across 384-well plates using an acoustic Echo Dispenser (LabCyte). Ebselen and DMSO were used as positive and negative controls, respectively. M^{pro} (0.3 µM) was dispensed across 384-well plates (25 µL/well) using a Multidrop Combi dispenser (Thermo Scientific) and incubated for 15 min at rt. TSAVLQ/SGFRK-NH_2 peptide solution (4 µM) was then dispensed to the mixture (25 µL/well). Reactions were incubated (10 minutes), then quenched by addition of 10% (v/v) aqueous formic acid (5 µL/well). Data was extracted and processed as reported.[16]

Protein Observed Mass Spectrometry

The reactions of compounds **1-12** with recombinant M^{pro} were investigated using protein observed mass spectrometry coupled to solid phase extraction purification as reported.[16] In brief, the compounds were dispensed across 384 well plates using an acoustic Echo Dispenser (LabCyte). M^{pro} was dispensed across 384-well plates using a Multidrop Combi dispenser (Thermo Scientific). The plates were then centrifuged (500 g, 15 s, Axygen Plate Spinner Centrifuge, Corning). Final reaction conditions: 40 µM compound, 2 µM M^{pro}, 20 mM HEPES, pH 7.5, 50 mM NaCl, 50µL final volume/well. The mass spectrometer was operated in positive ion mode with the following parameters: drying gas temperature (225 °C), drying gas flow rate

(13 L/min), nebulizer pressure (40 psig), sheath gas temperature (350 °C), sheath gas flow rate (12 L/min), capillary voltage (4000 V), nozzle voltage (1000 V).

References

[1] S. Mehta, D. Brahmchari, *J. Org. Chem.* **2019**, *84*, 5492-5503.

[2] B. S. Bhakuni, A. Kumar, S. J. Balkrishna, J. A. Sheikh, S. Konar, S. Kumar, *Org. Lett.* **2012**, *14*, 2838-2841.

[3] L. Yadav, M. K. Tiwari, B. R. K. Shyamlal, S. Chaudhary, *J. Org. Chem.* **2020**, *85*, 8121-8141.

[4] A. J. Pacuła, J. Ścianowski, K. B. Aleksandrzak, *RSC Adv.* **2014**, *4*(90), 48959-48962.

[5] M. Hellal, G. D. Cuny, *Tetrahedron Lett.* **2011**, *52*, 5508-5511.

[6] J. S. Kumar, G. S. Reddy, R. Medishetti, A. Ray, S. D. Bele, K. A. Hossain, B. Thirupataiah, R. K. Edwin, P. Behera, A. Joseph, G. G. Shenoy, C. M. Rao, M. Pal, *J. Mol. Struct.* **2021**, *1240*, 130574.

[7] A. D. Landgraf, A. S. Alsegiani, S. Alaqel, S. Thanna, Z. A. Shah, S. J. Sucheck, *ACS Chem. Neurosci.* **2020**, *11*, 3008-3016.

[8] F. Viani, B. Rossi, W. Panzeri, L. Merlini, A. M. Martorana, A. Polissi, Y. M. Galante, *Tetrahedron* **2017**, *73*, 1745-1761.

[9] V. Kavala, C.-C. Wang, Y.-H. Wang, C.-W. Kuo, D. Janreddy, W.-C. Huang, T.-S. Kuo, C. H. He, M.-L. Chen, C.-F. Yao, *Adv. Synth. Catal.* **2014**, *356*, 2609-2626.

[10] B. S. Bhakuni, S. J. Balkrishna, A. Kumar, S. J. T. L. Kumar, *Tetrahedron Lett.* **2012**, *53*, 1354-1357.

[11] H. X. Ngo, S. K. Shrestha, S. Garneau-Tsodikova, *ChemMedChem* **2016**, *11*, 1507-1516.

[12] L. Yang, L. Song, S. Tang, L. Li, H. Li, B. Yuan, G. Yang, *Eur. J. Org. Chem.* **2019**, *2019*, 1281-1285.

[13] B. R. Stockwell, A. Zask, A. Kaplan, WO2020113222A1, **2020**.

[14] K. Yang, H. Zhang, B. Niu, T. Tang, H. Ge, *Eur. J. Org. Chem.* **2018**, *2018*, 5520-5523.

[15] Z. Wang, Y. Kuninobu, M. Kanai, *J. Org. Chem.* **2013**, *78*, 7337-7342.

[16] T. R. Malla, A. Tumber, T. John, L. Brewitz, C. Strain-Damerell, C. D. Owen, P. Lukacik, H. T. H. Chan, P. Maheswaran, E. Salah, F. Duarte, H. Yang, Z. Rao, M. A. Walsh, C. J. Schofield, *Chem. Commun.* **2021**, *57*, 1430-1433.

[17] Z. Jin, X. Du, Y. Xu, Y. Deng, M. Liu, Y. Zhao, B. Zhang, X. Li, L. Zhang, C. Peng, Y.Duan, J. Yu, L. Wang, K. Yang, F. Liu, R. Jiang, X. Yang, T. You, X. Liu, X. Yang, F. Bai, H. Liu, X. Liu, L. W. Guddat, W. Xu, G. Xiao, C. Qin, Z. Shi, H. Jiang, Z. Rao, H. Yang, *Nat.* **2020**, *582*, 289-293.

NMR Spectra

^{1}H NMR of **13** (400 MHz, $CDCl_3$)

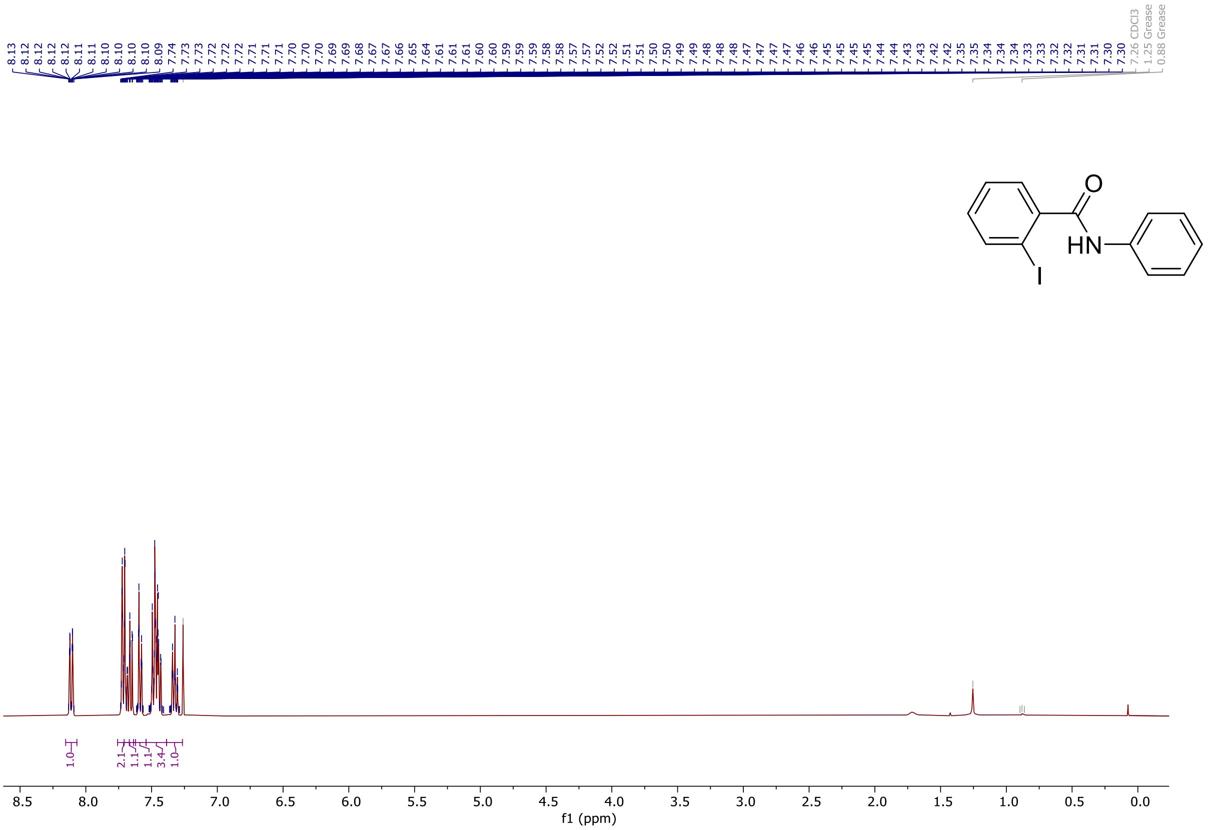

^{13}C NMR of **13** (101 MHz, $CDCl_3$)

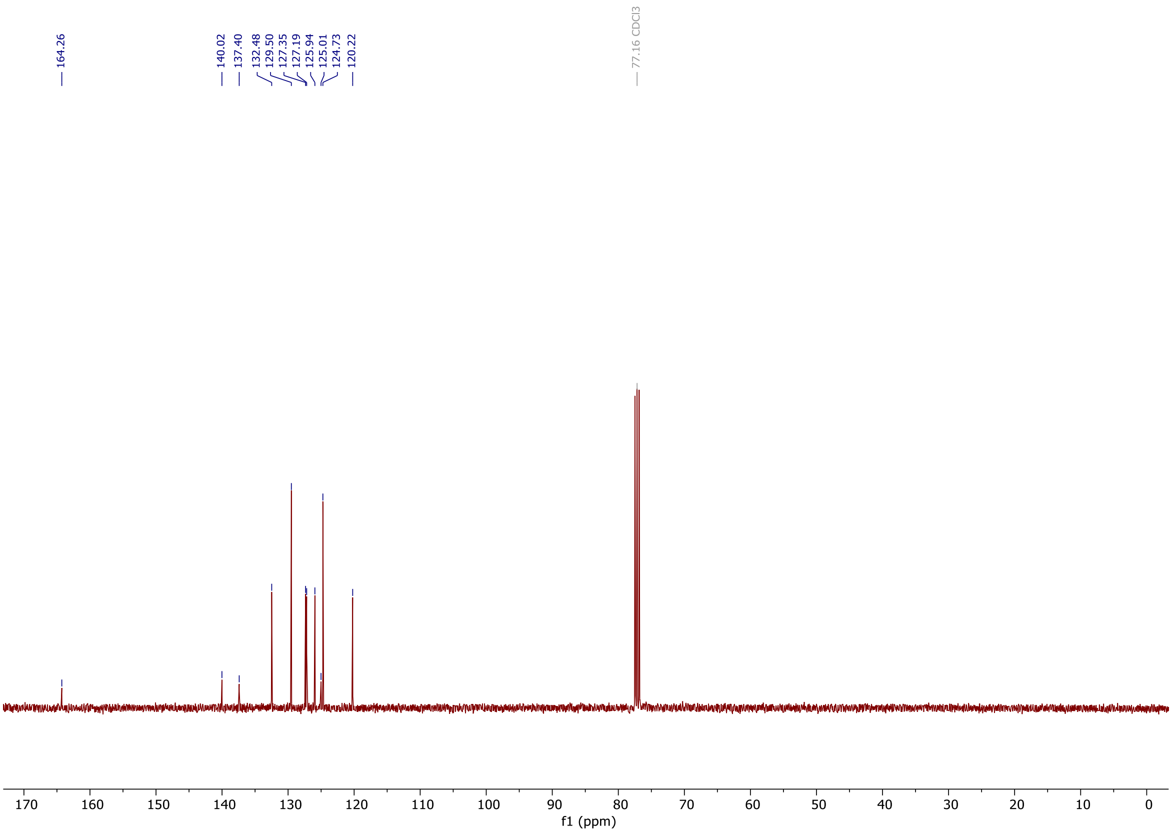

^{1}H NMR of **14** (400 MHz, $CDCl_3$)

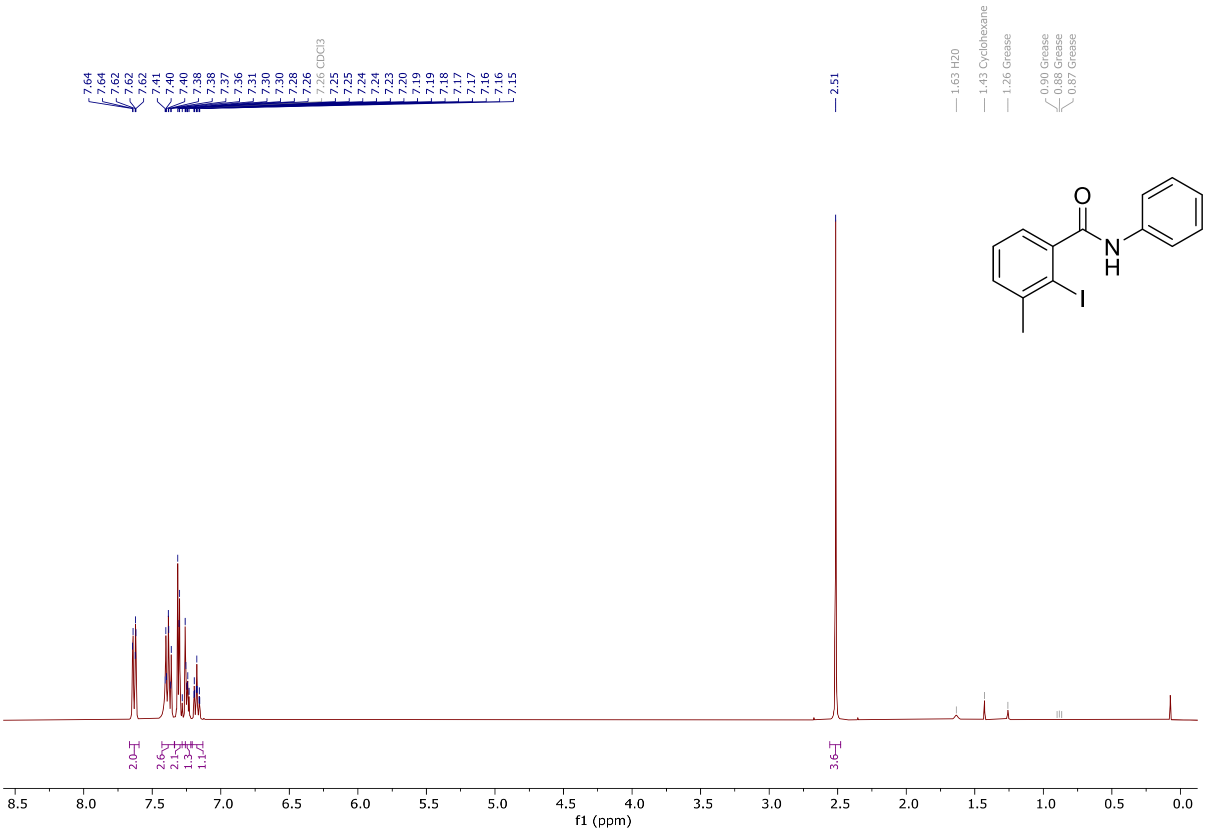

^{13}C NMR of **14** (101 MHz, $CDCl_3$)

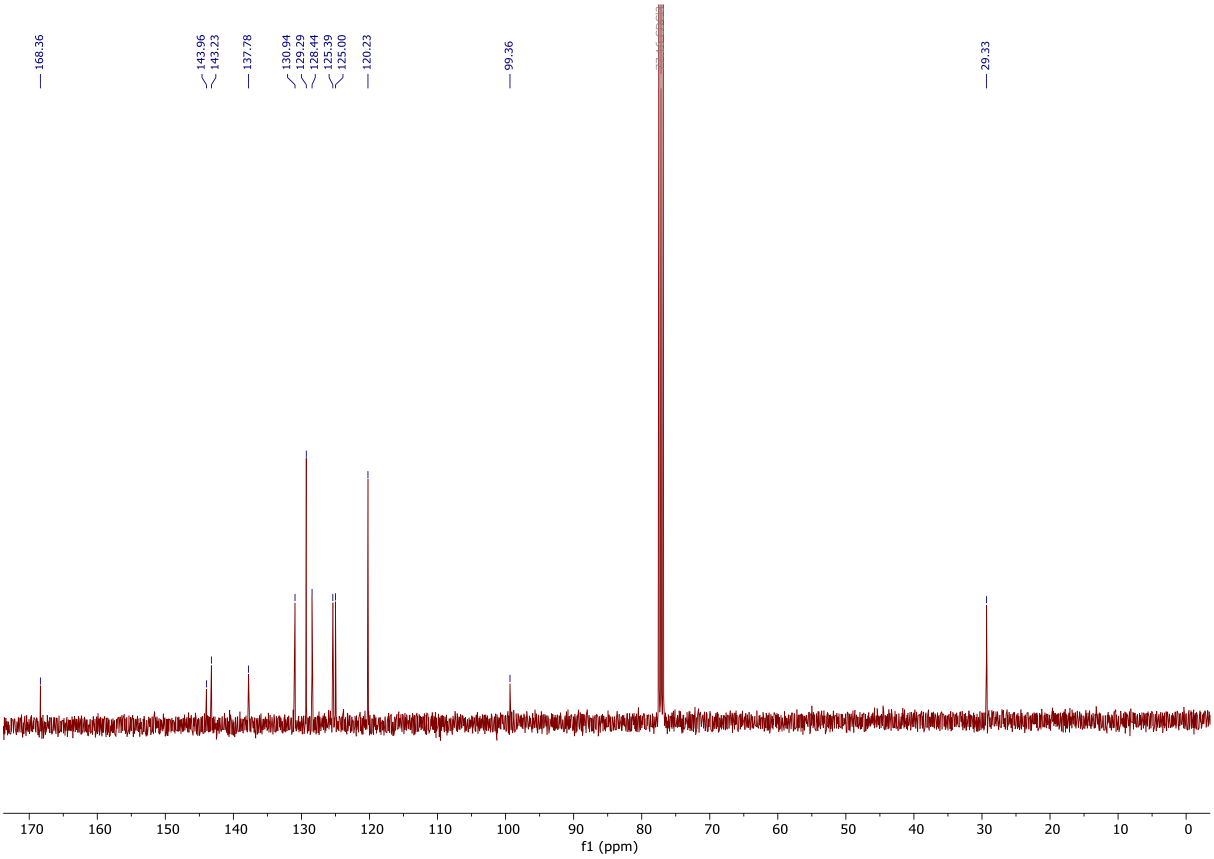

^{1}H NMR of **15** (400 MHz, $CDCl_3$)

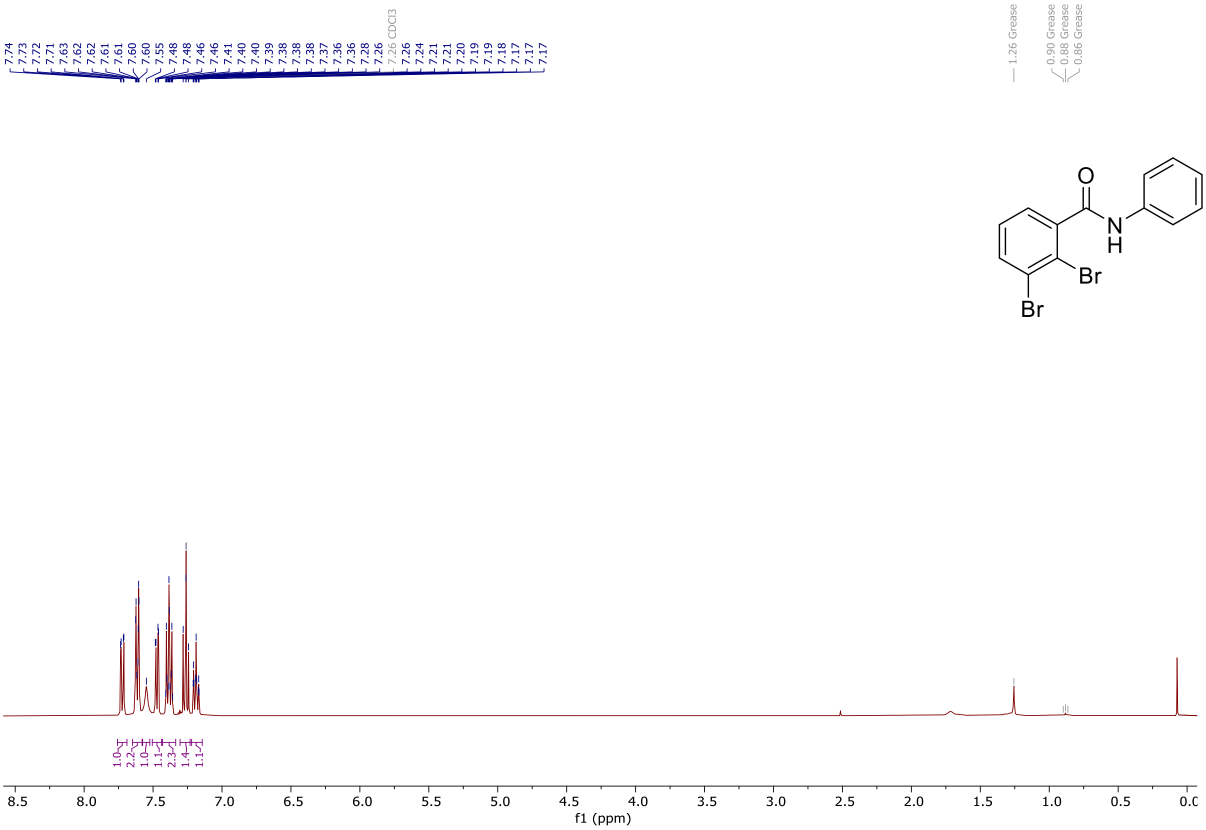

^{13}C NMR of **15** (101 MHz, $CDCl_3$)

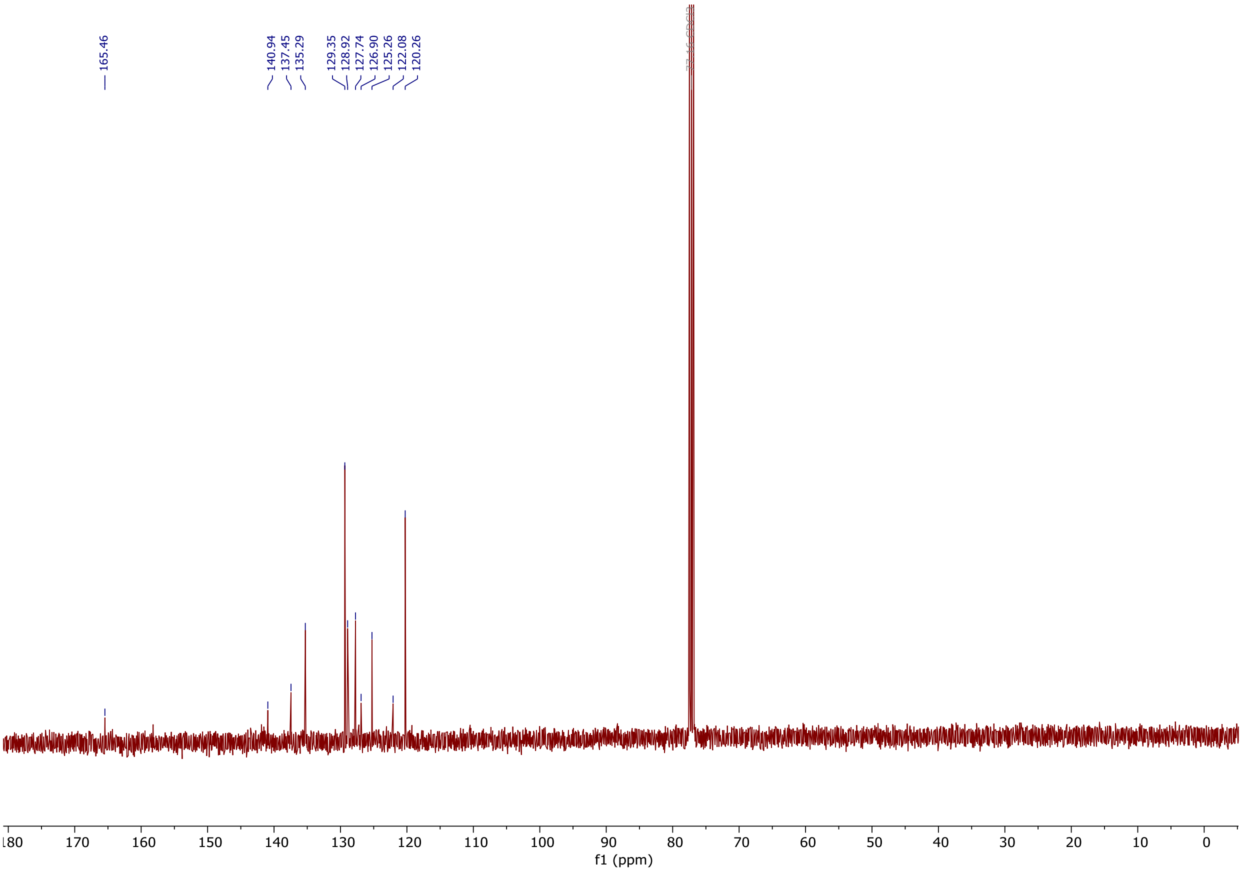

^{1}H NMR of **16** (400 MHz, $CDCl_3$)

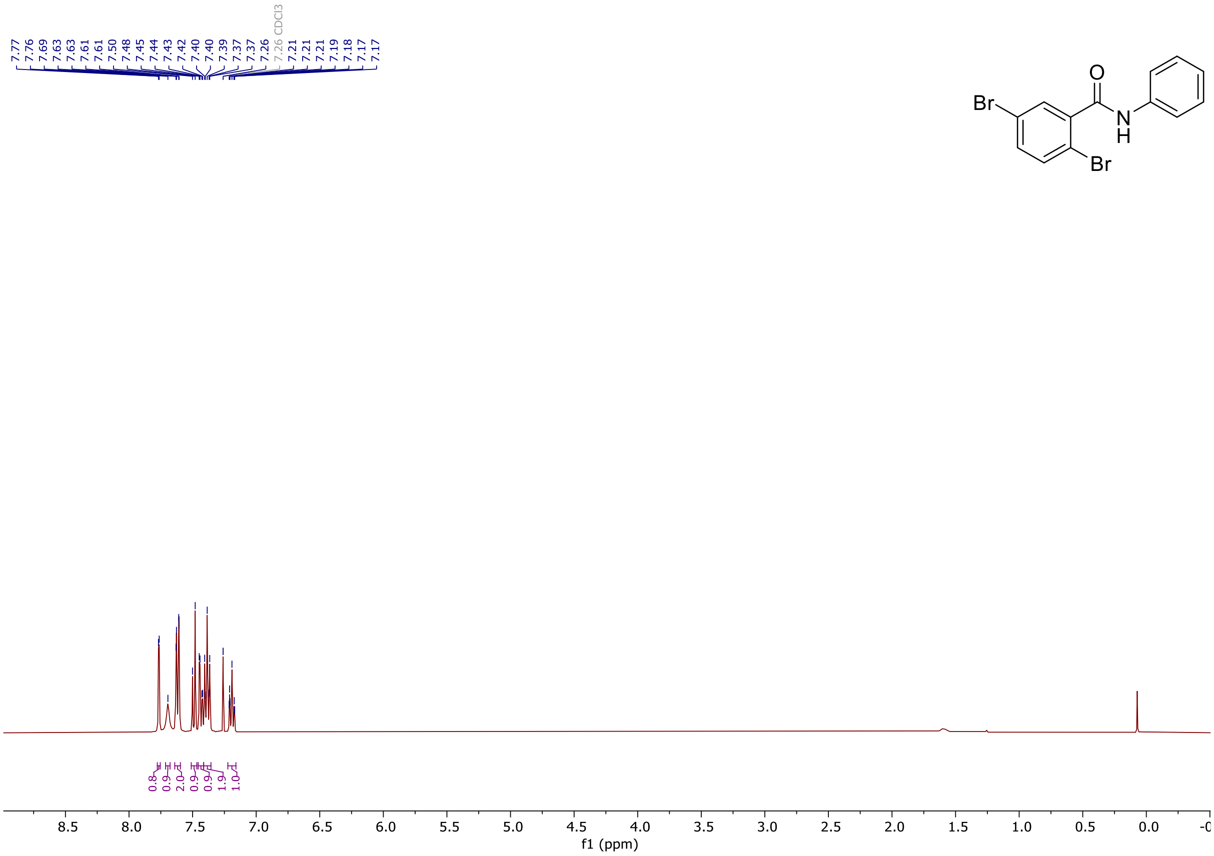

^{13}C NMR of **16** (101 MHz, $CDCl_3$)

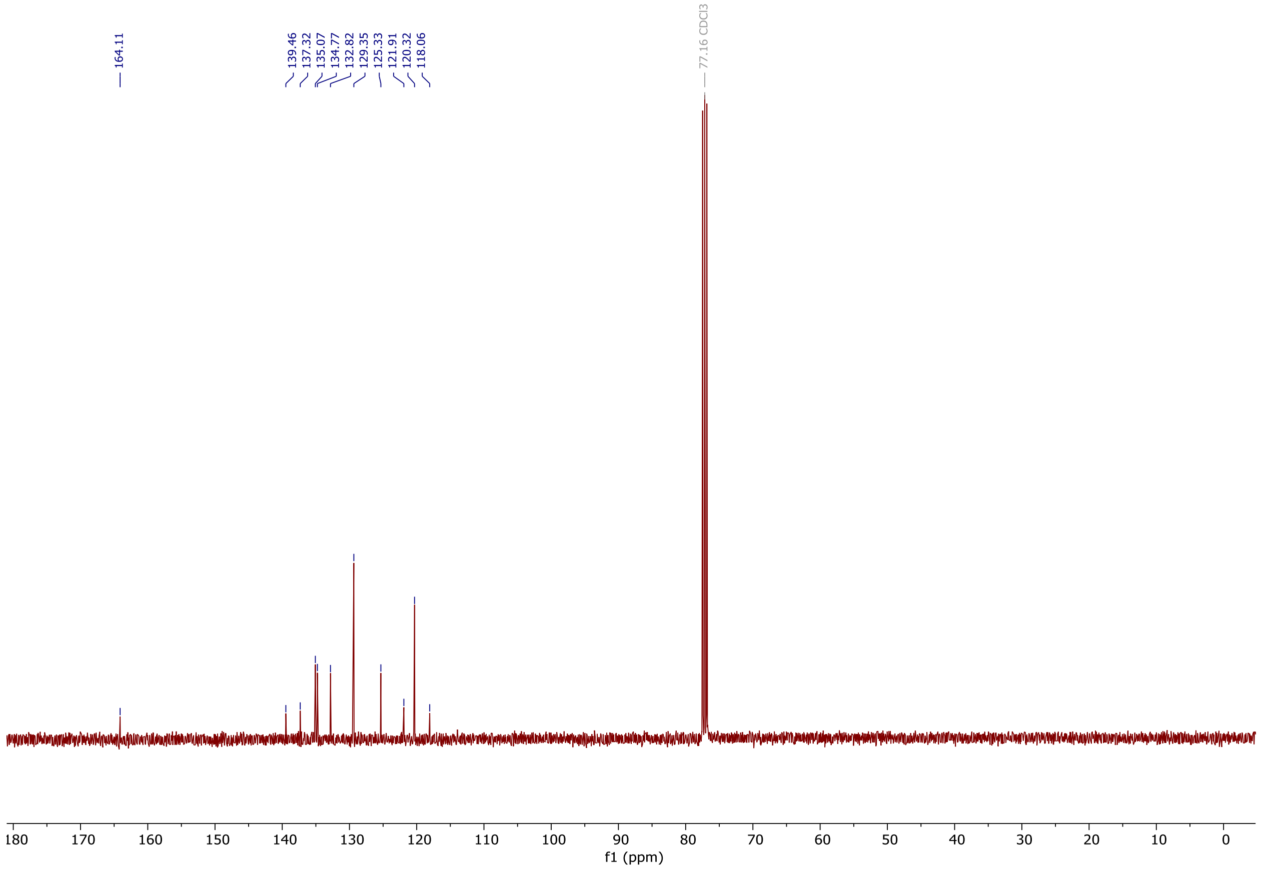

^{1}H NMR of **17** (400 MHz, $CDCl_3$)

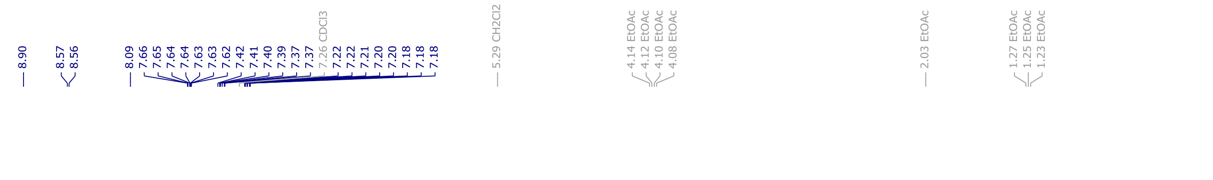

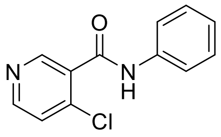

^{13}C NMR of **17** (101 MHz, $CDCl_3$)

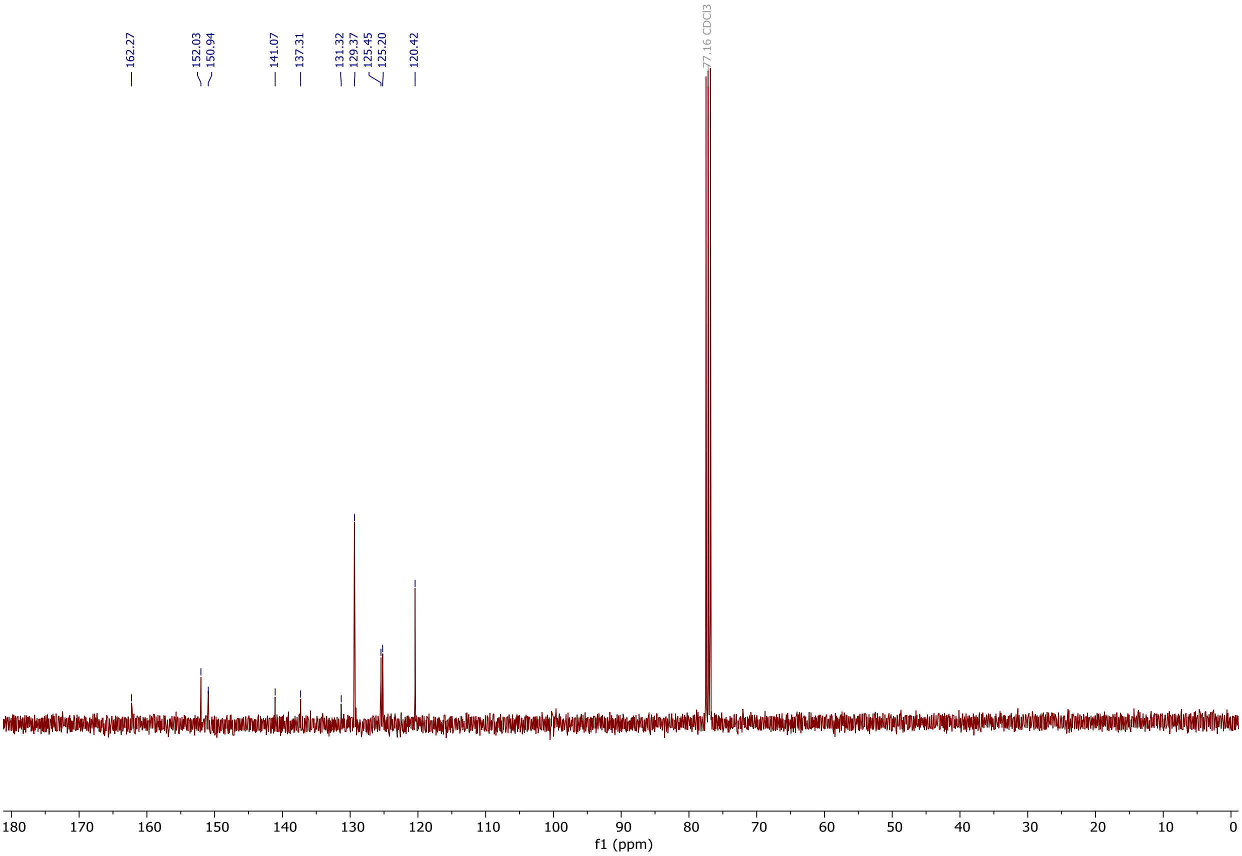

^{1}H NMR of **18** (400 MHz, $CDCl_3$)

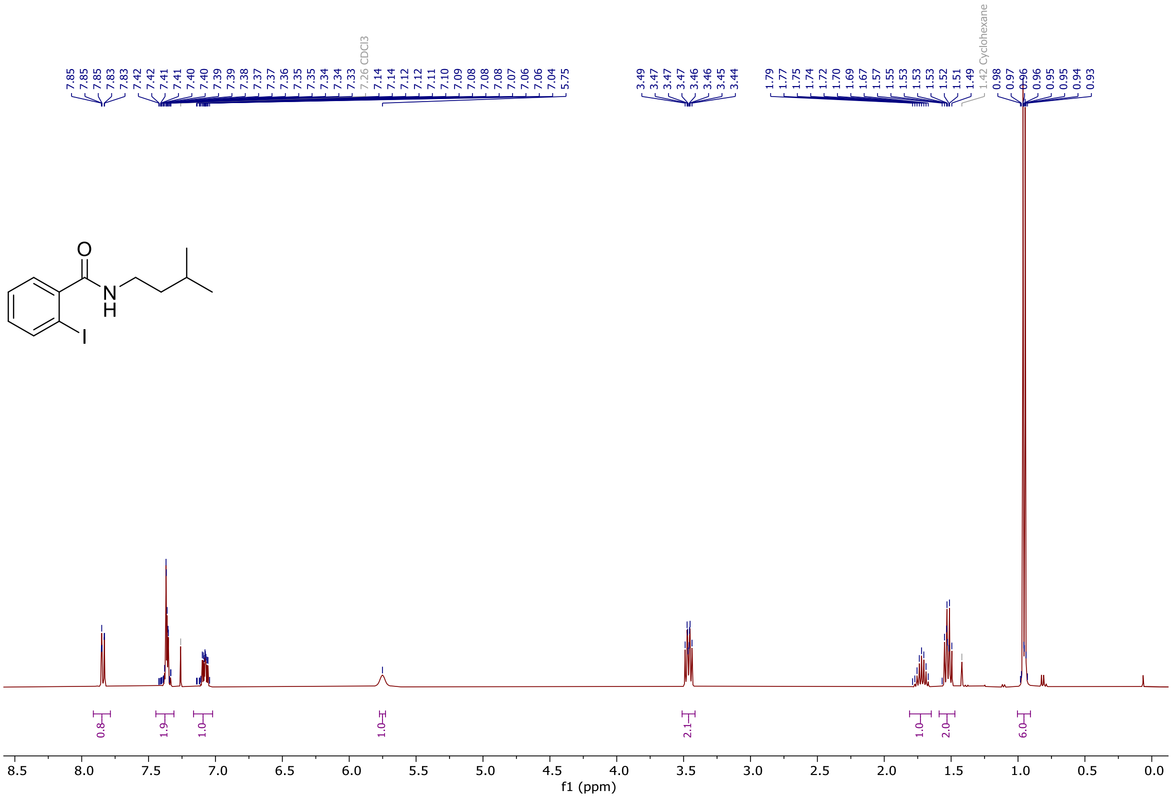

^{13}C NMR of **18** (101 MHz, $CDCl_3$)

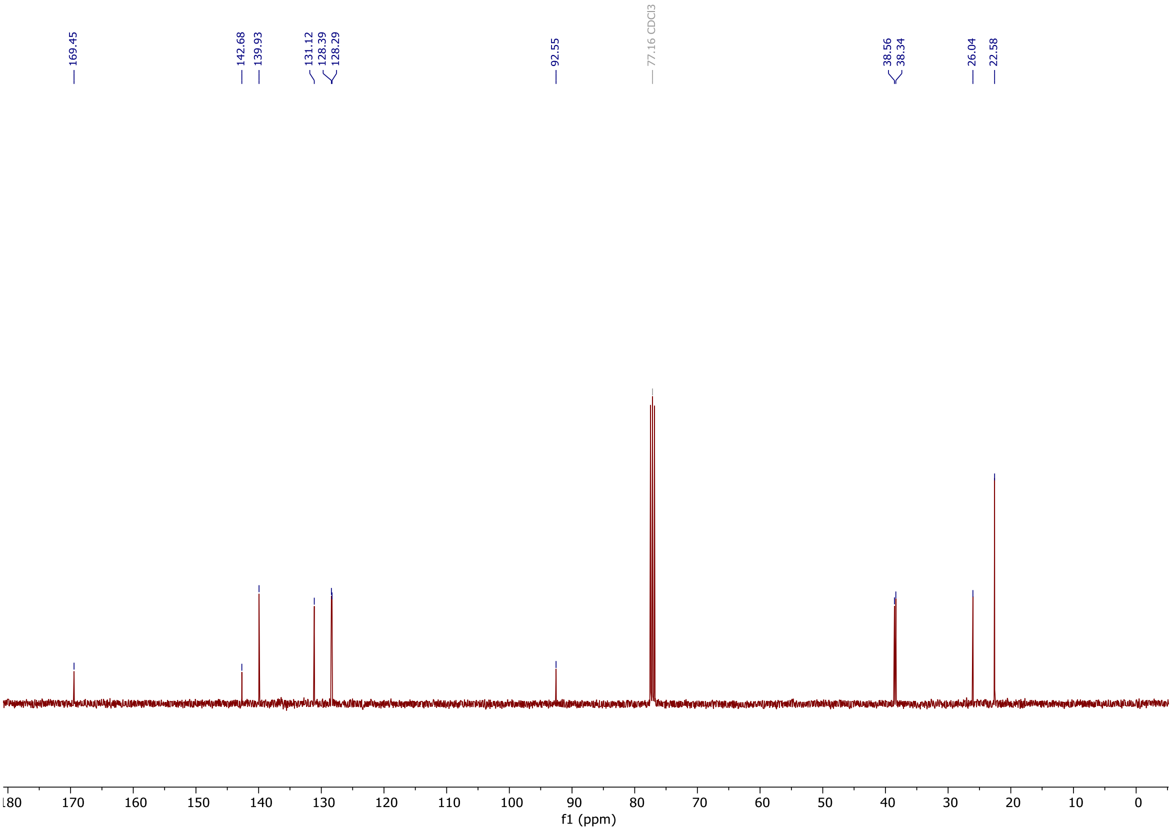

^{1}H NMR of **19** (400 MHz, $CDCl_3$)

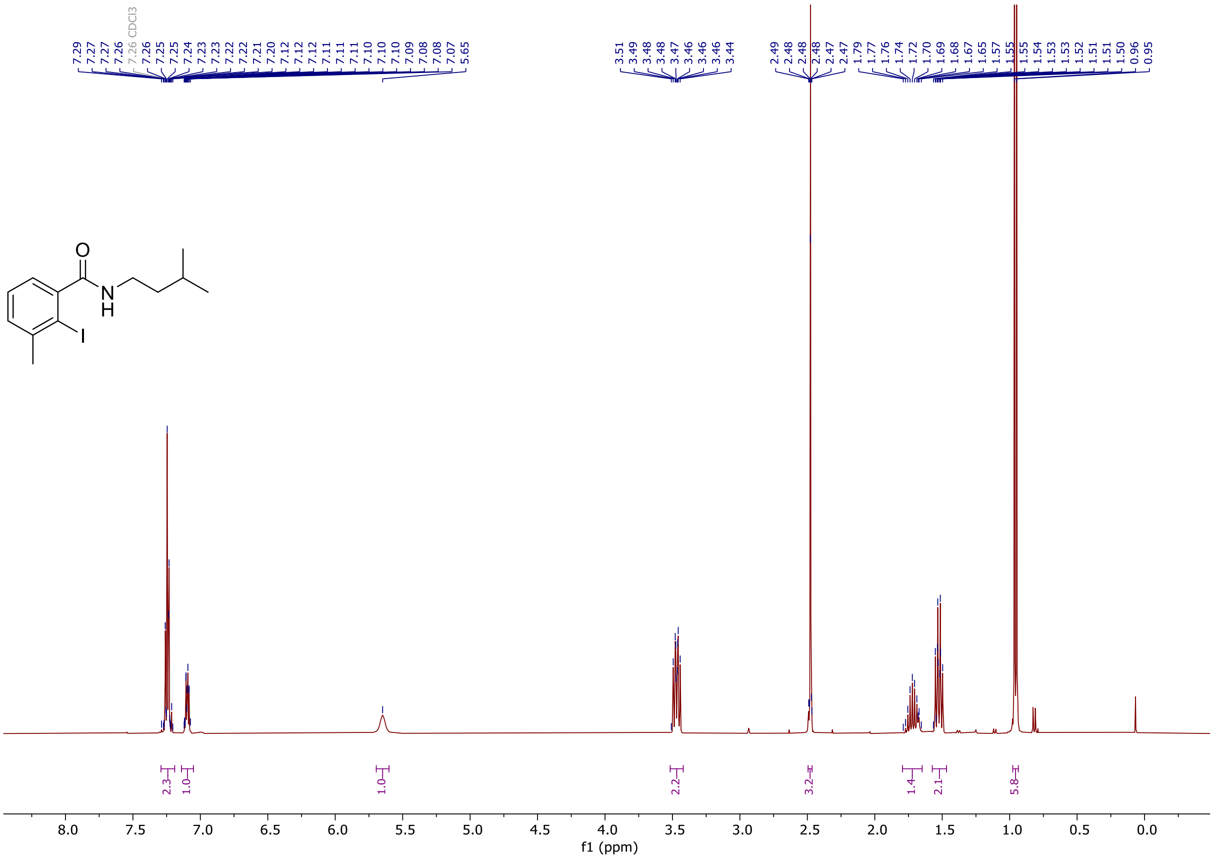

^{13}C NMR of **19** (101 MHz, $CDCl_3$)

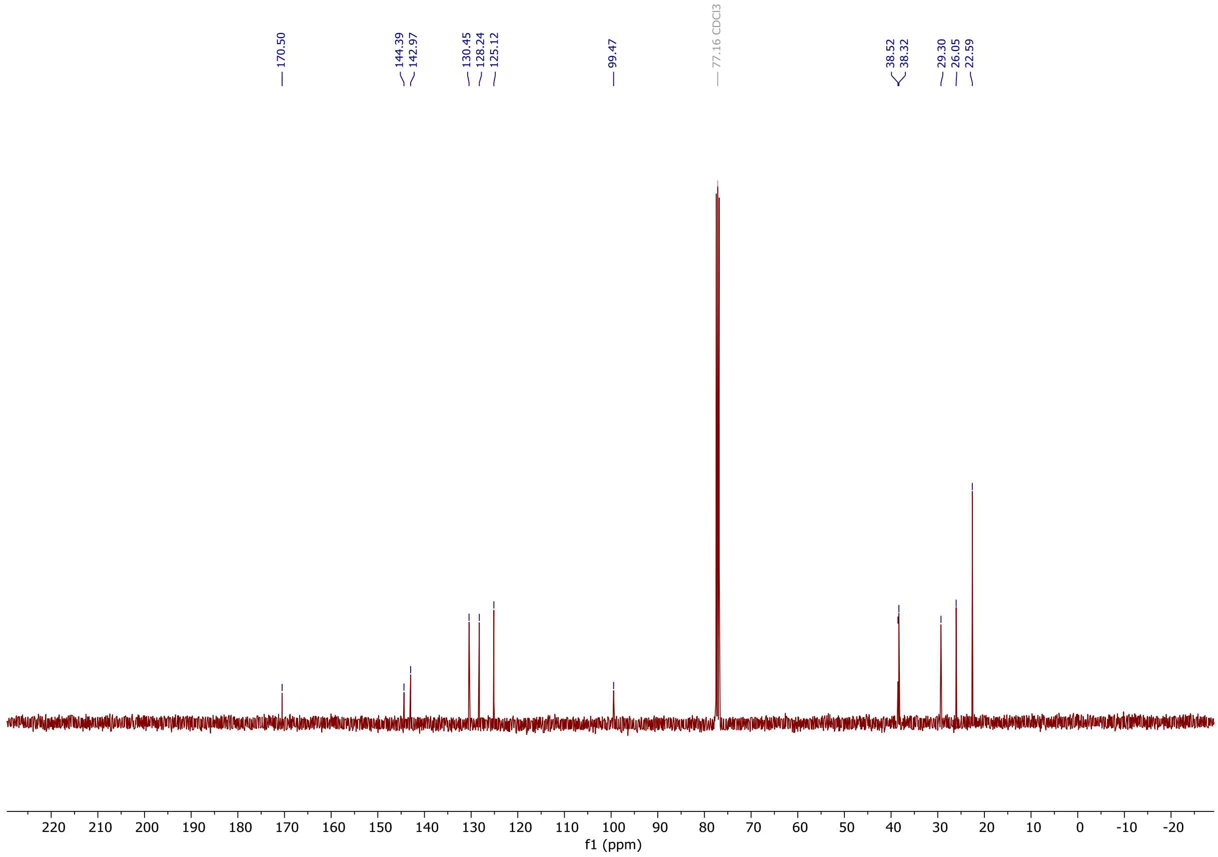
170.50
144.39
142.97
130.45
128.24
125.12
99.47
77.16 CDCl3
38.52
38.32
29.30
26.05
22.59
220 210 200 190 180 170 160 150 140 130 120 110 100 90 80 70 60 50 40 30 20 10 0 -10 -20
f1 (ppm)

^{1}H NMR of **20** (400 MHz, $CDCl_3$)

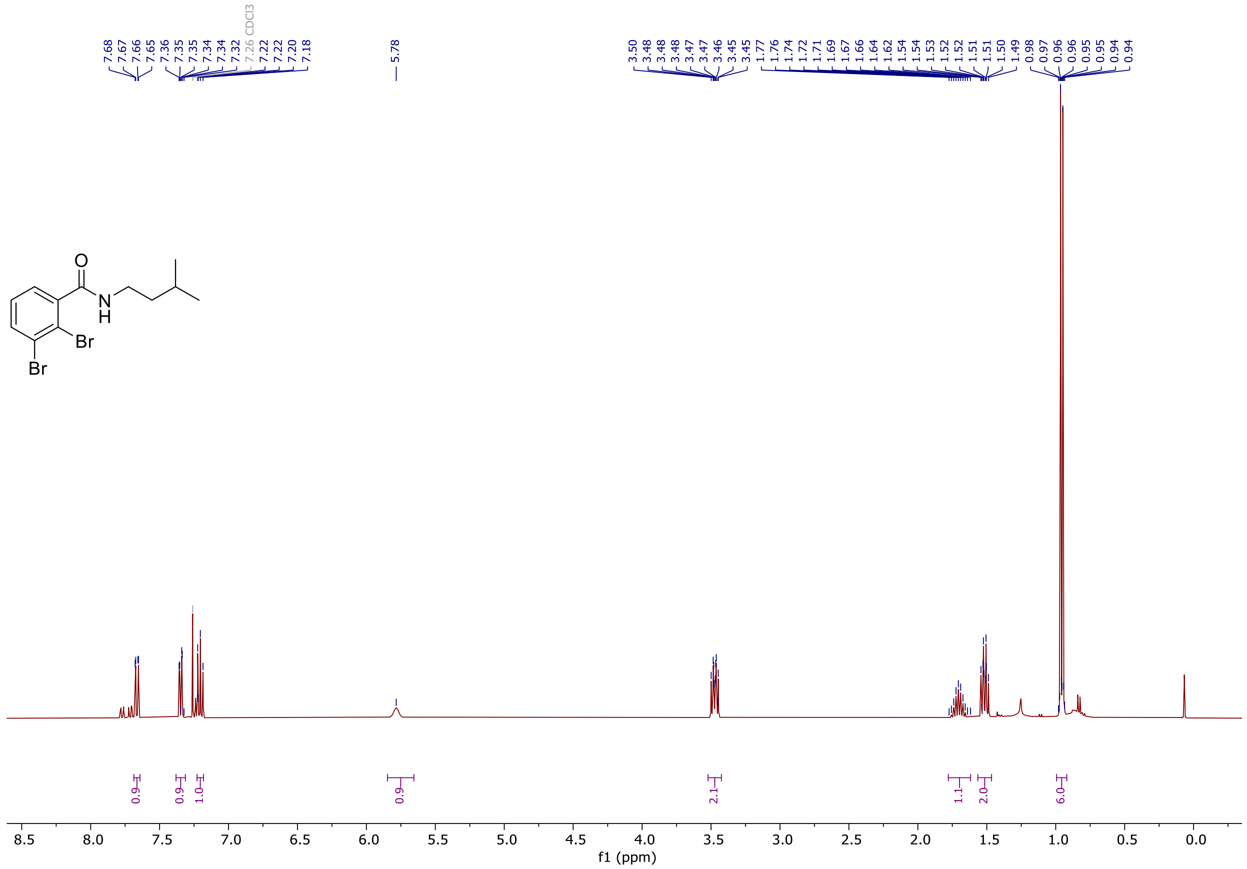

^{13}C NMR of **20** (101 MHz, $CDCl_3$)

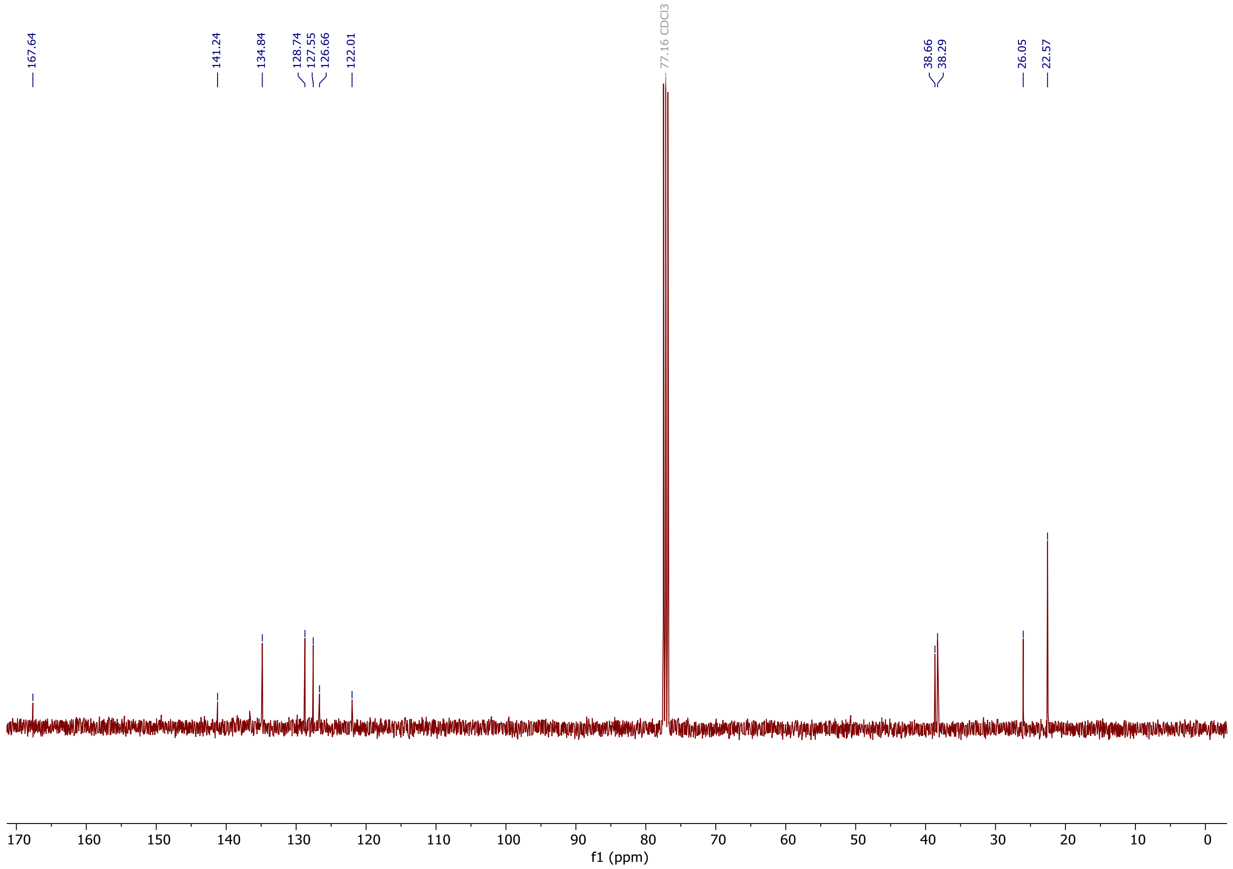

^{1}H NMR of **21** (400 MHz, $CDCl_3$)

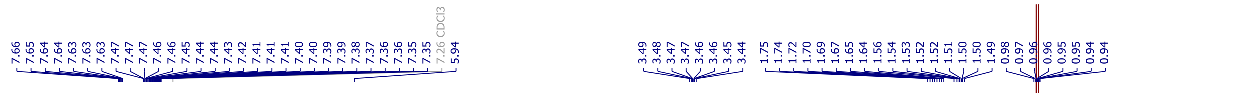

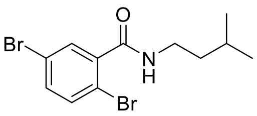

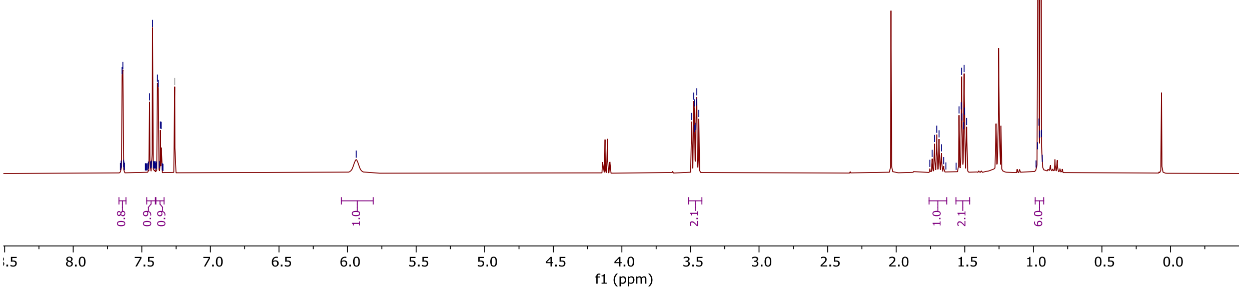

^{13}C NMR of **21** (101 MHz, $CDCl_3$)

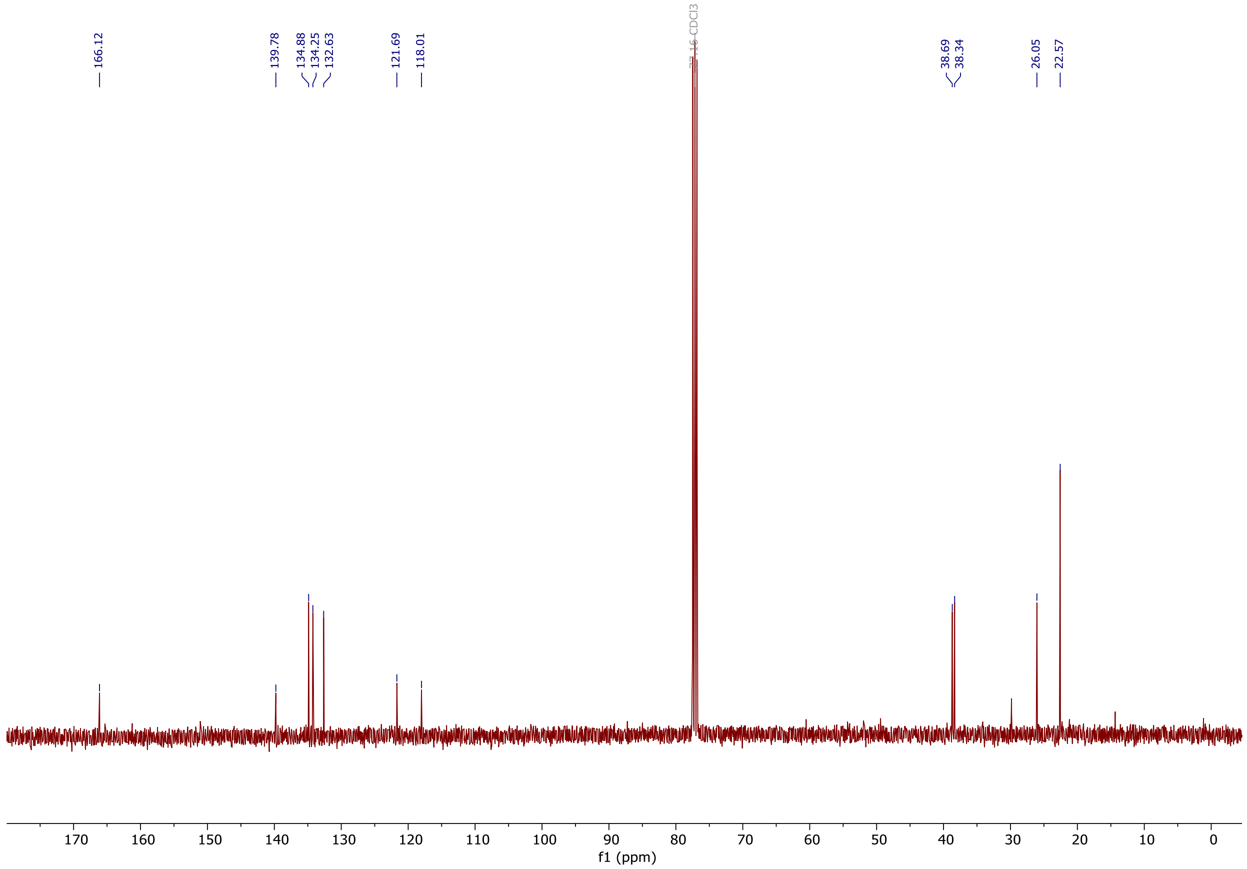

^{1}H NMR of **22** (400 MHz, $CDCl_3$)

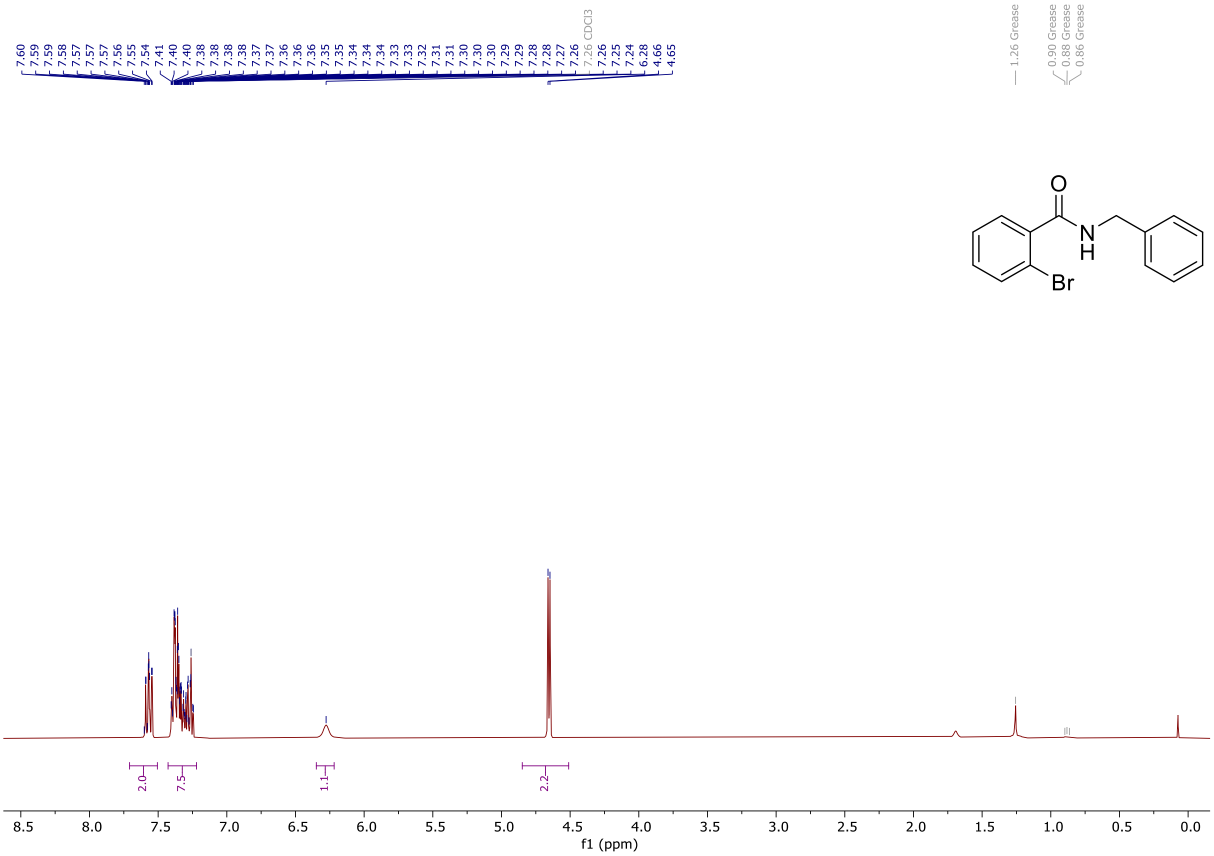

^{13}C NMR of **22** (101 MHz, $CDCl_3$)

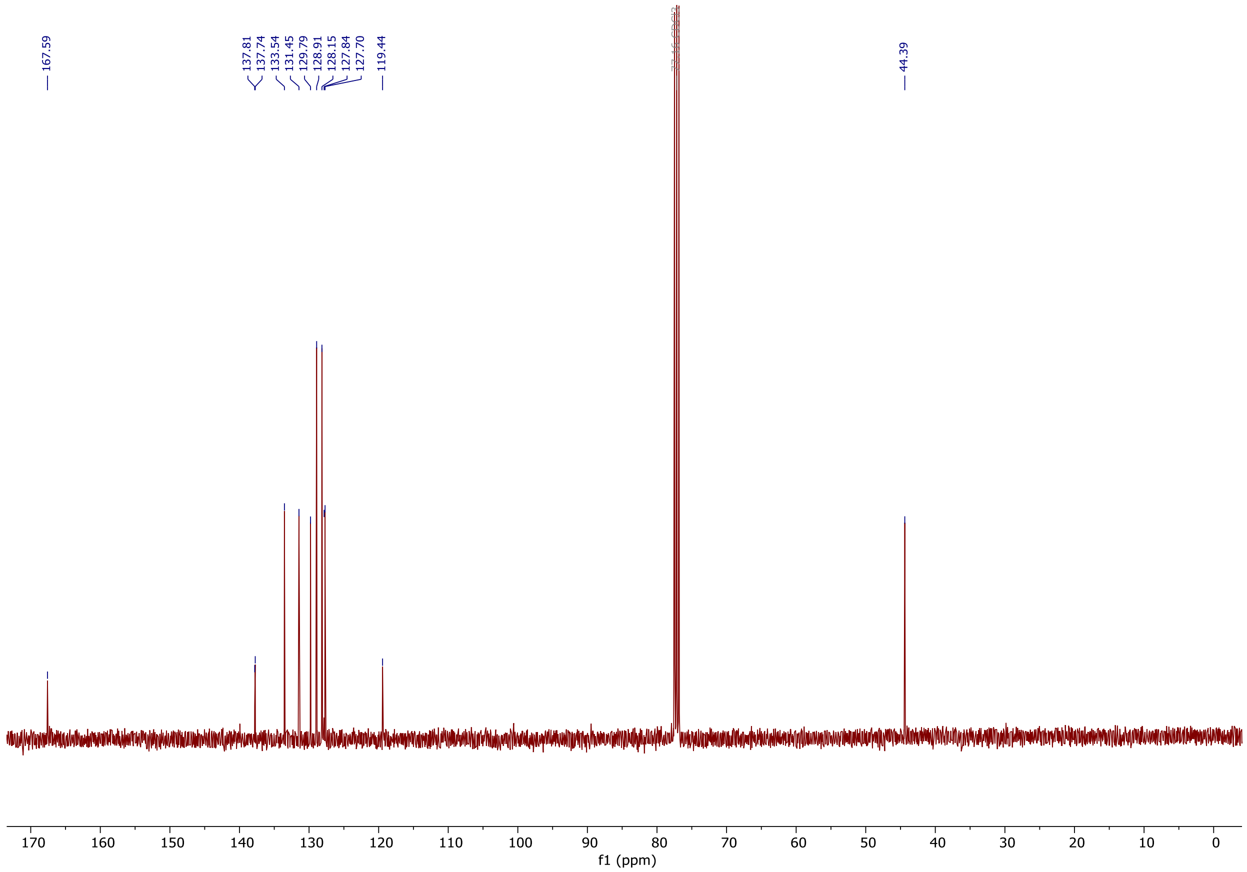

^{1}H NMR of **23** (400 MHz, $CDCl_3$)

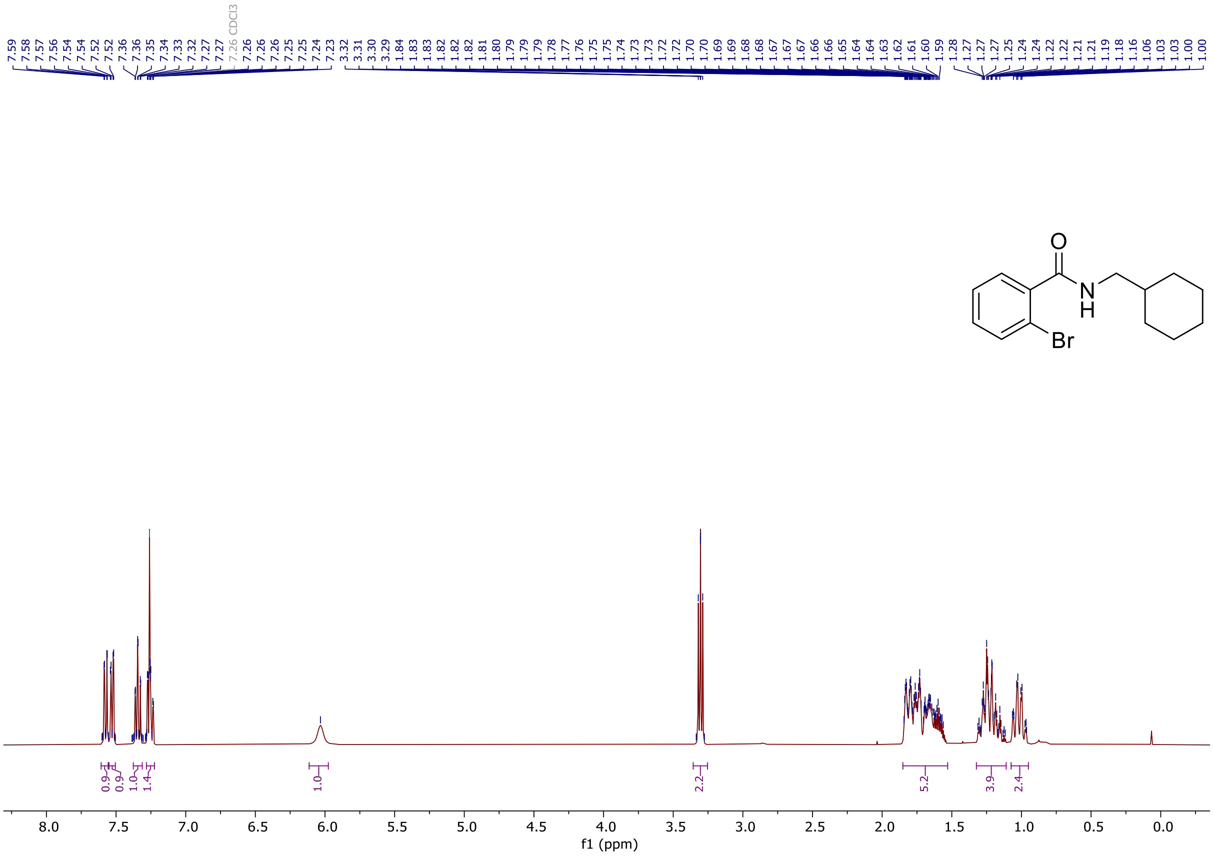

^{13}C NMR of **23** (101 MHz, $CDCl_3$)

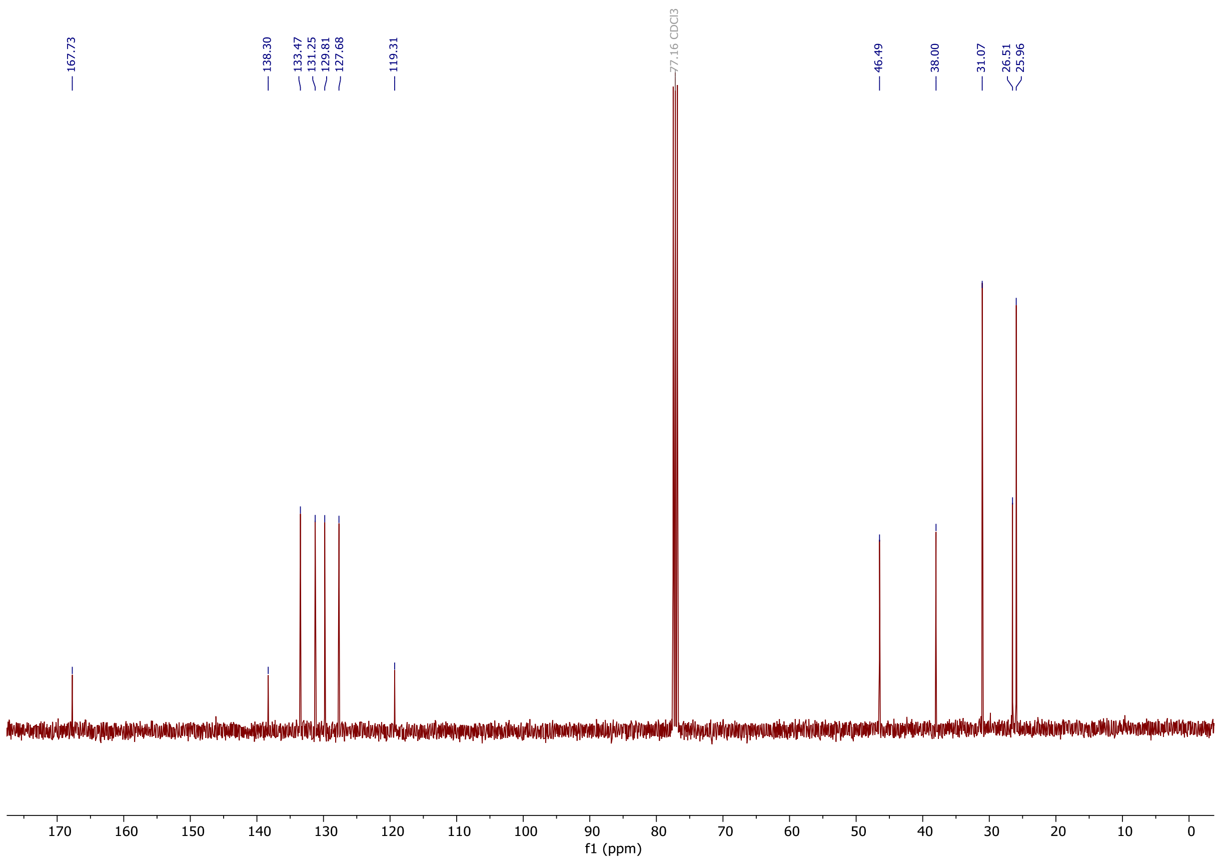
167.73
138.30
133.47
131.25
129.81
127.68
119.31
77.16 CDCl3
46.49
38.00
31.07
26.51
25.96
170
160
150
140
130
120
110
100
90
80
70
60
50
40
30
20
10
0
f1 (ppm)

^{1}H NMR of **24** (400 MHz, $CDCl_3$)

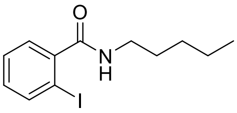

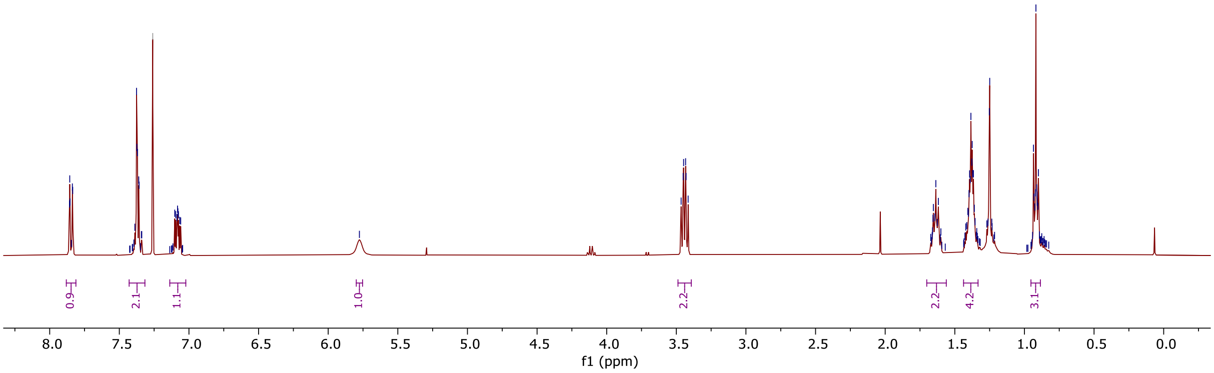

^{13}C NMR of **24** (101 MHz, $CDCl_3$)

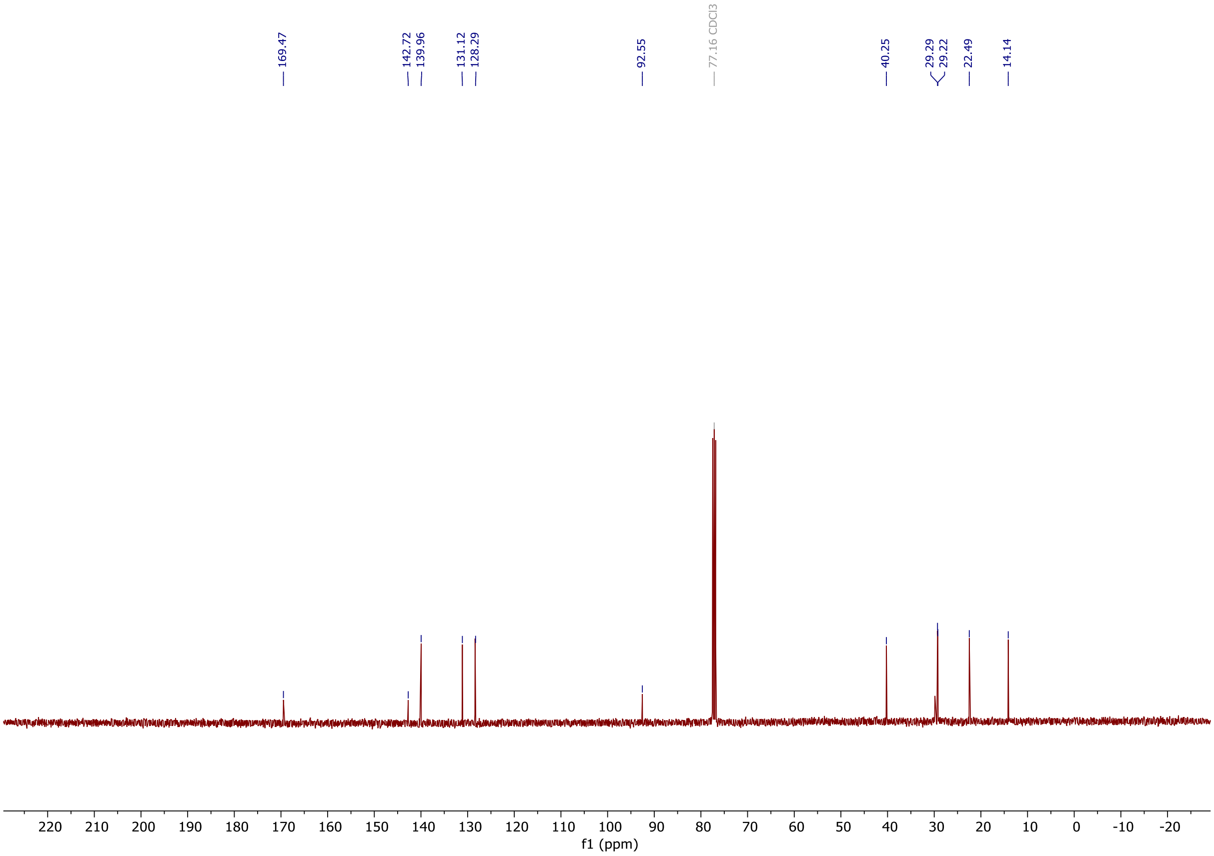

^{1}H NMR of **25** (400 MHz, $CDCl_3$)

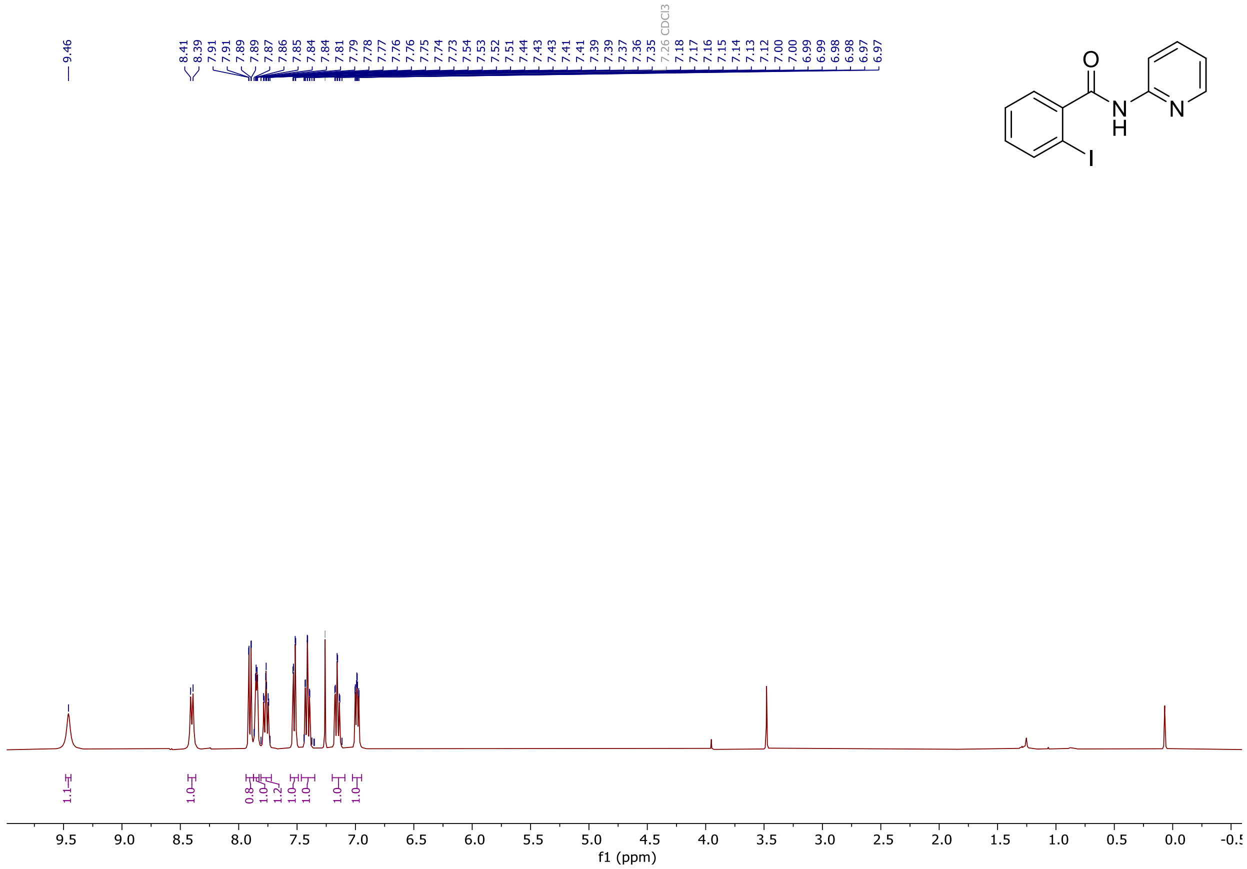

^{13}C NMR of **25** (101 MHz, $CDCl_3$)

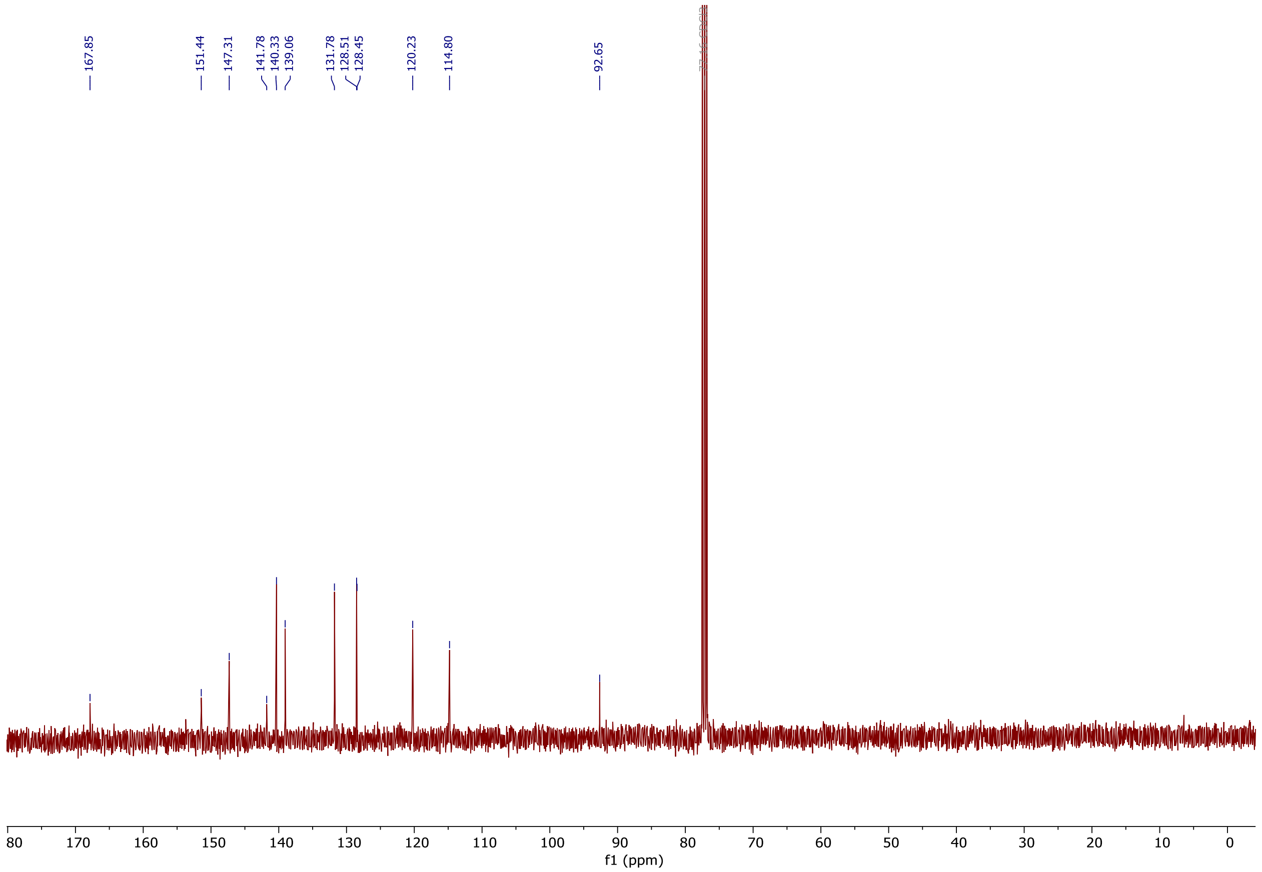

^{1}H NMR of **26** (400 MHz, C_2D_6OS)

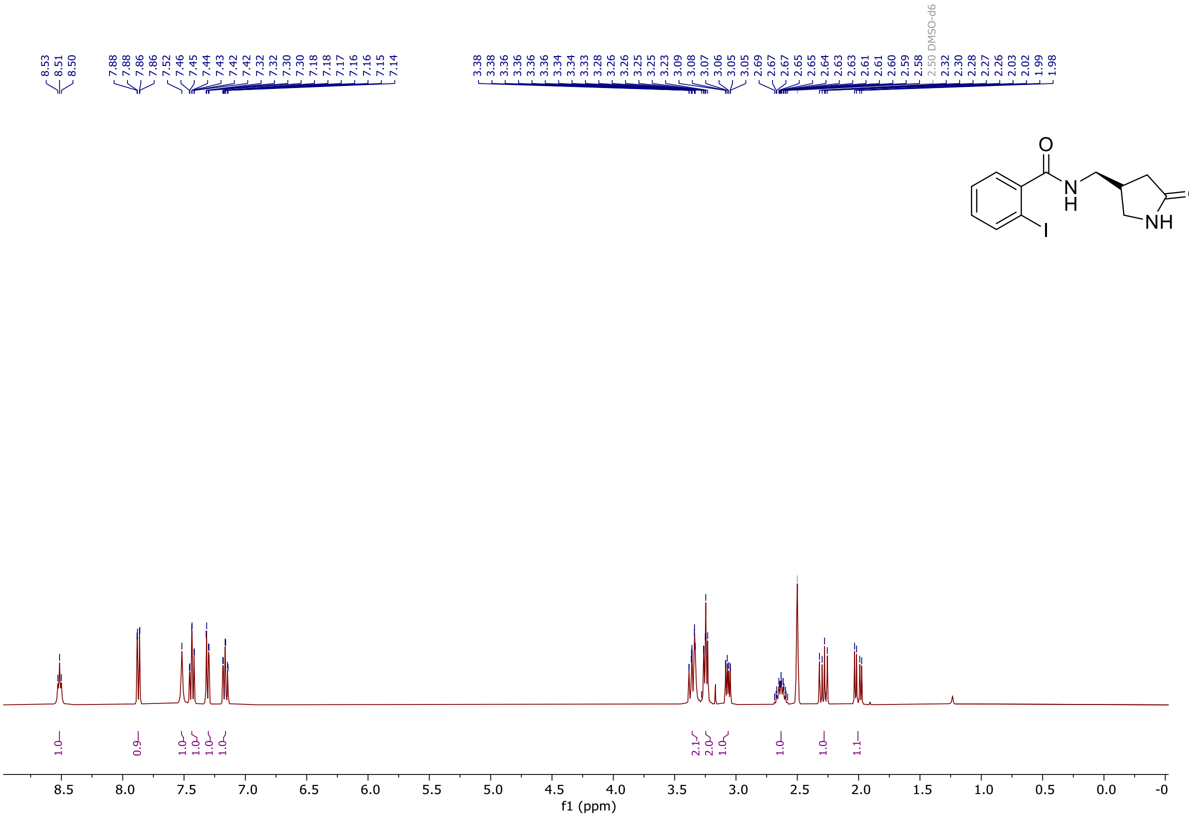

^{13}C NMR of **26** (101 MHz, C_2D_6OS)

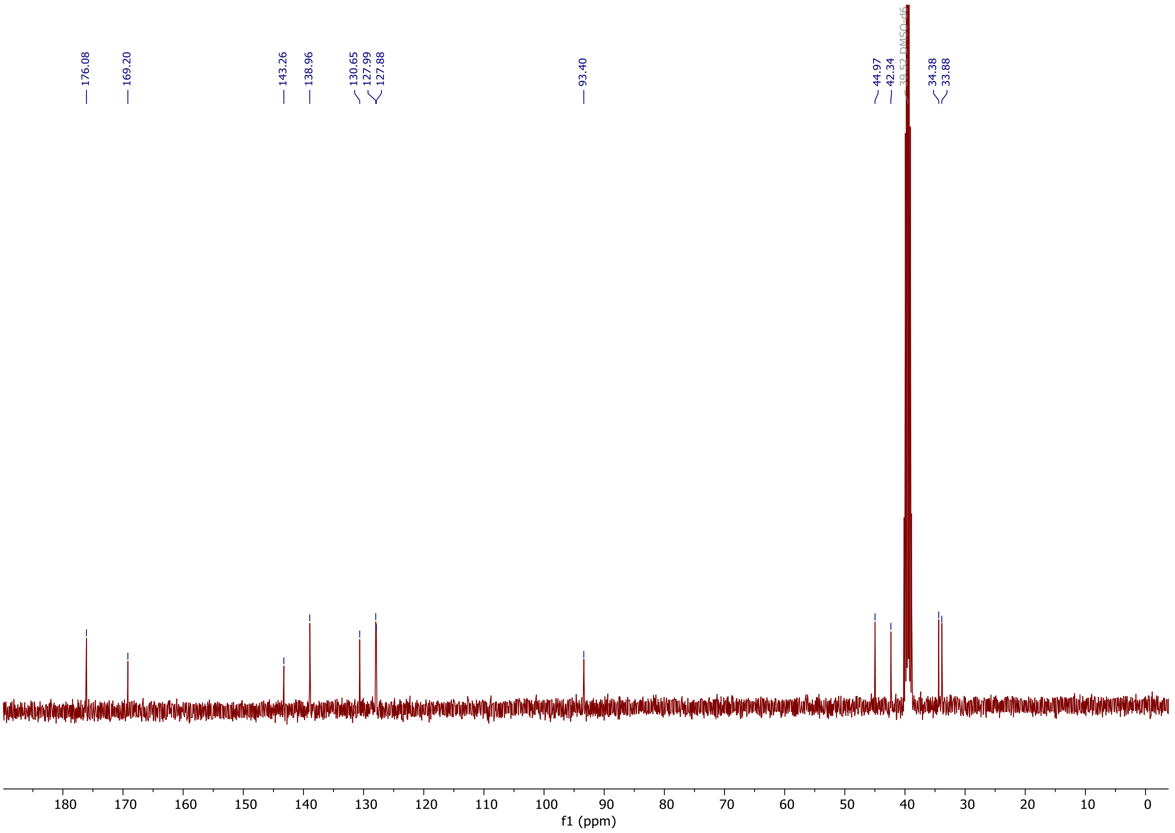

^{1}H NMR of **27** (400 MHz, $CDCl_3$)

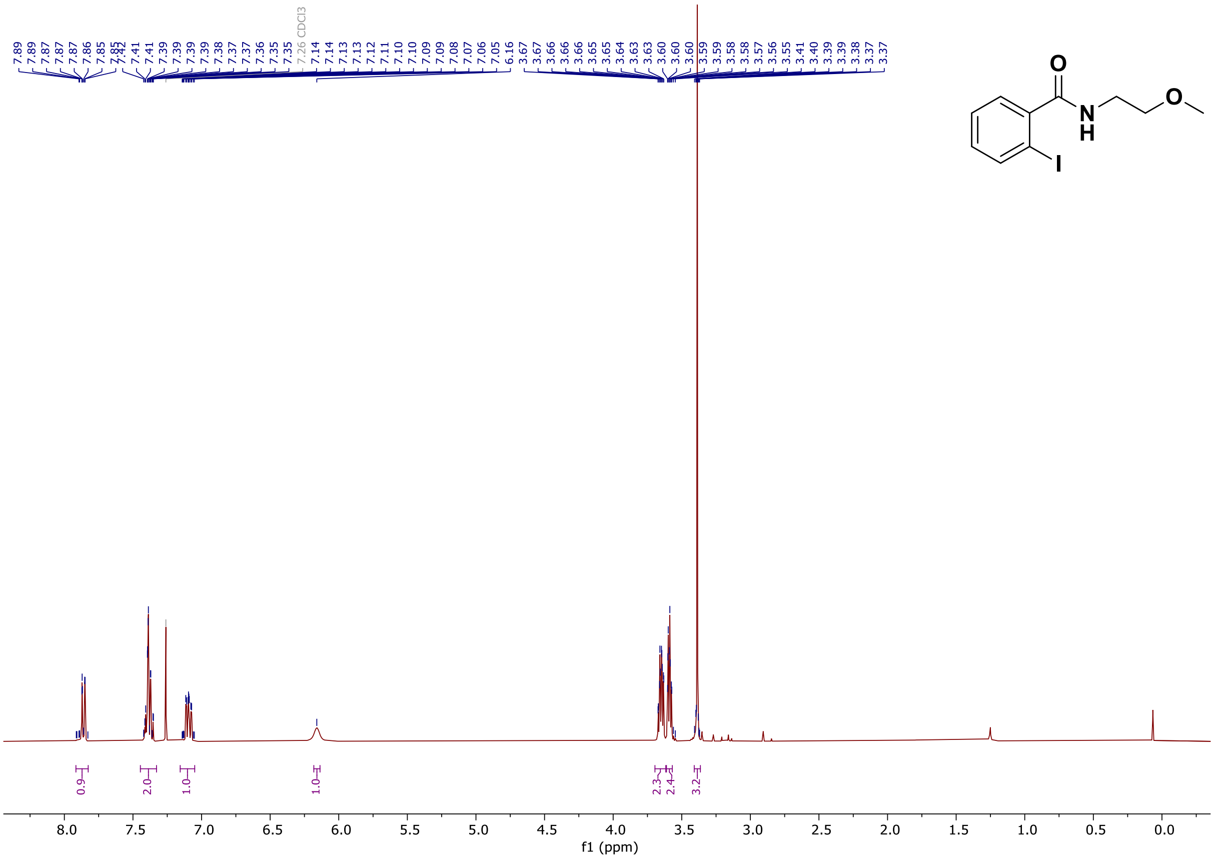

^{13}C NMR of **27** (101 MHz, $CDCl_3$)

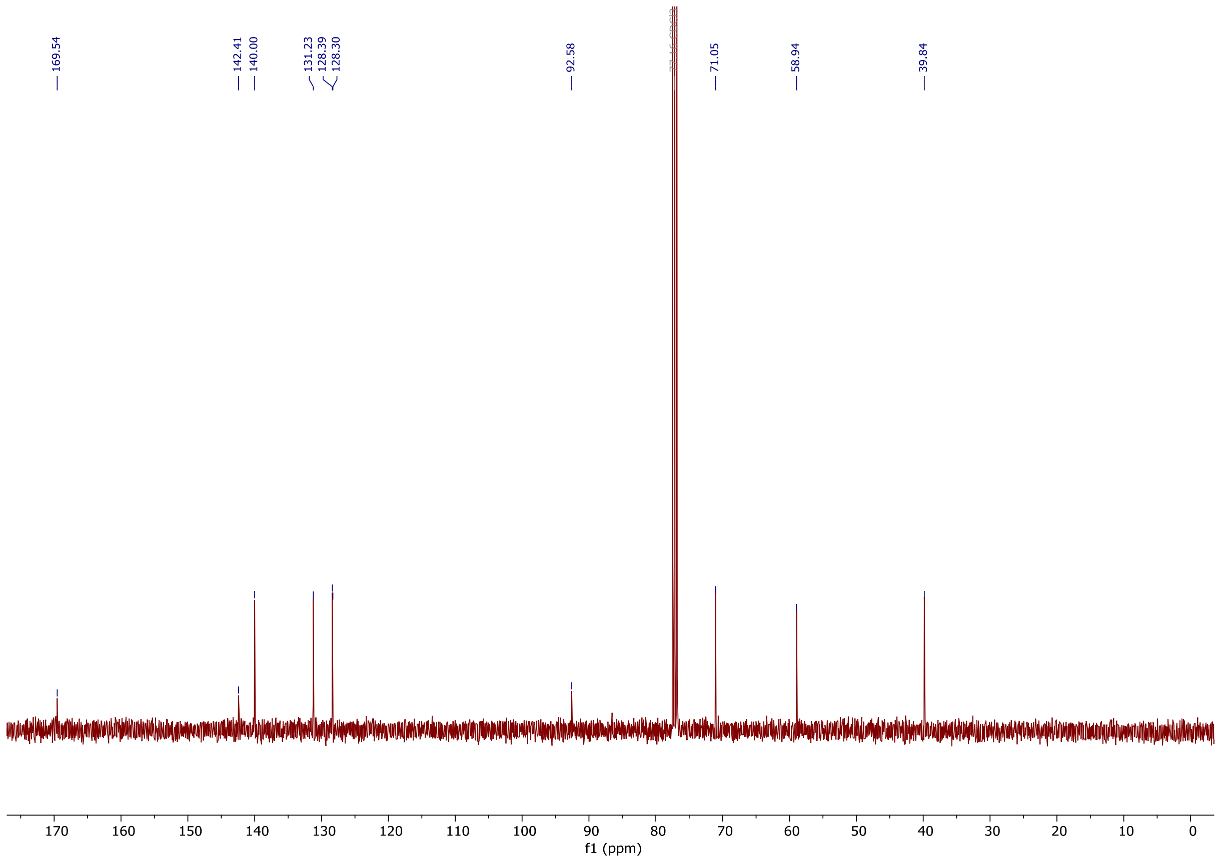
169.54
142.41
140.00
131.23
128.39
128.30
92.58
71.05
58.94
39.84
170 160 150 140 130 120 110 100 90 80 70 60 50 40 30 20 10 0
f1 (ppm)

^{1}H NMR of **28** (400 MHz, $CDCl_3$)

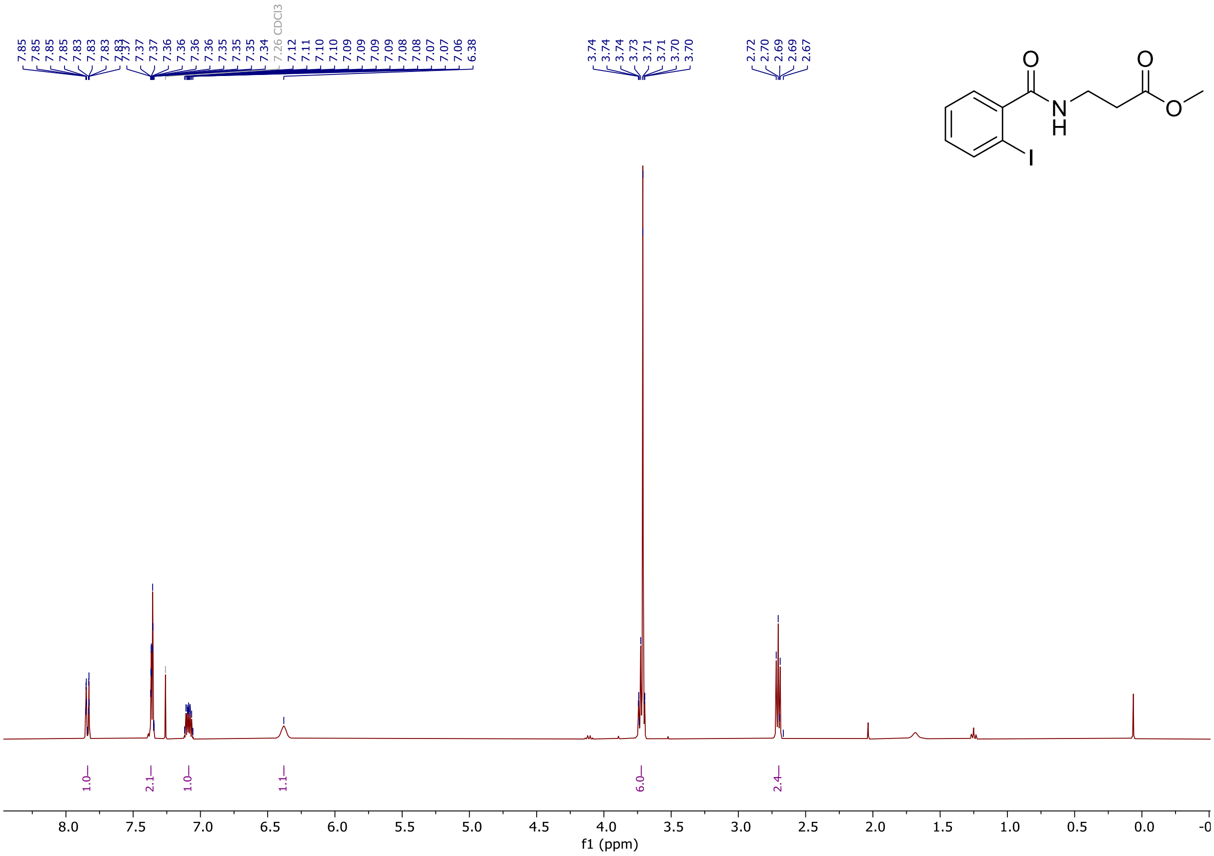

^{13}C NMR of **28** (101 MHz, $CDCl_3$)

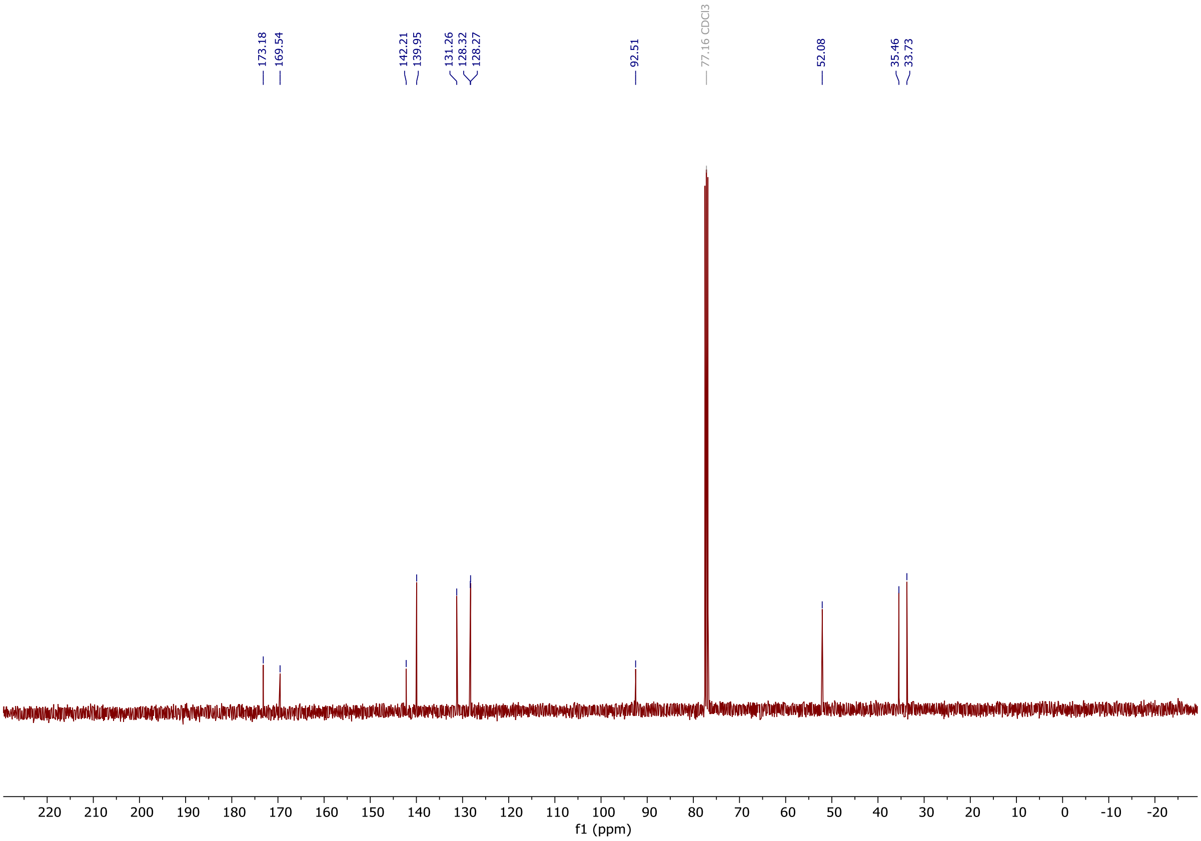

^{1}H NMR of **29** (400 MHz, $CDCl_3$)

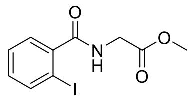

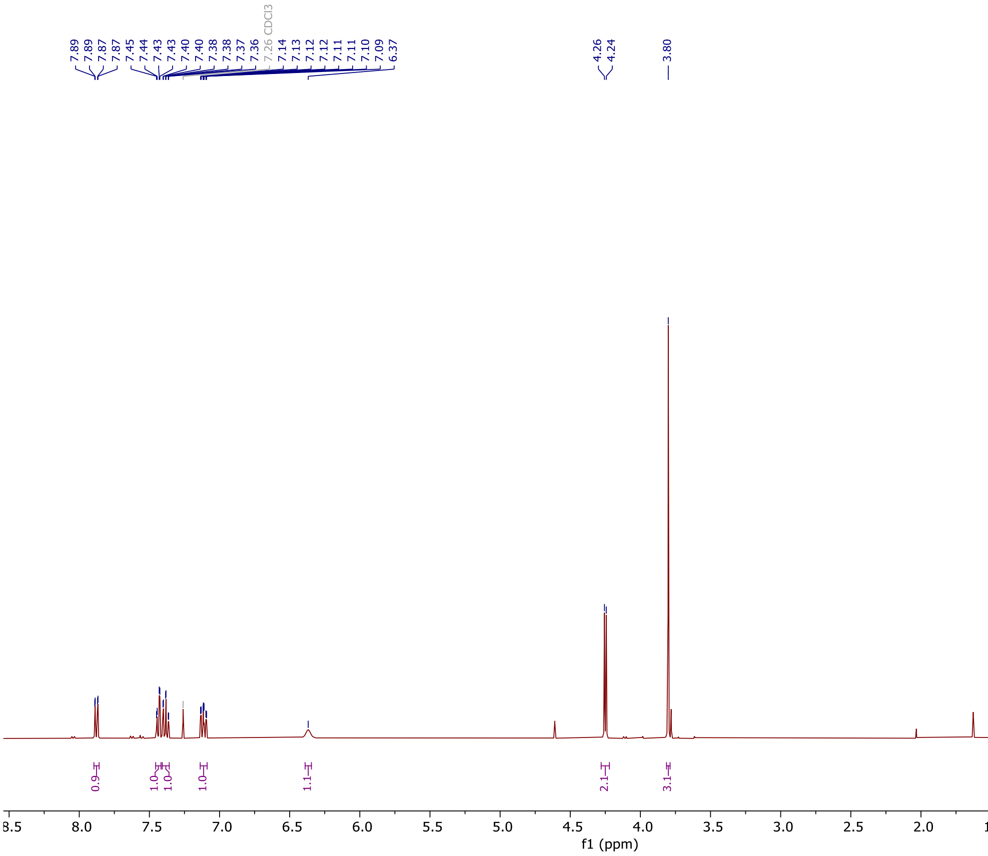

^{13}C NMR of **29** (101 MHz, $CDCl_3$)

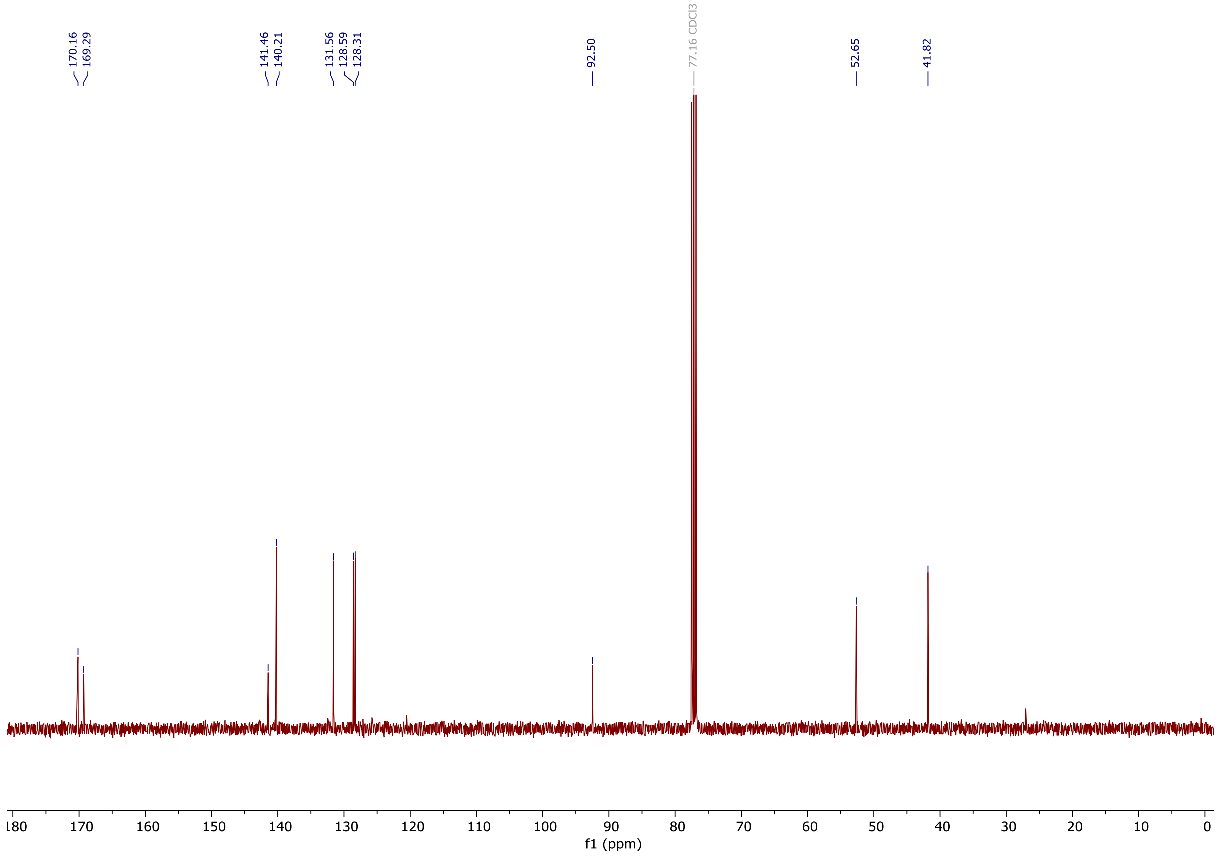

^{1}H NMR of **3a** (400 MHz, $CDCl_3$)

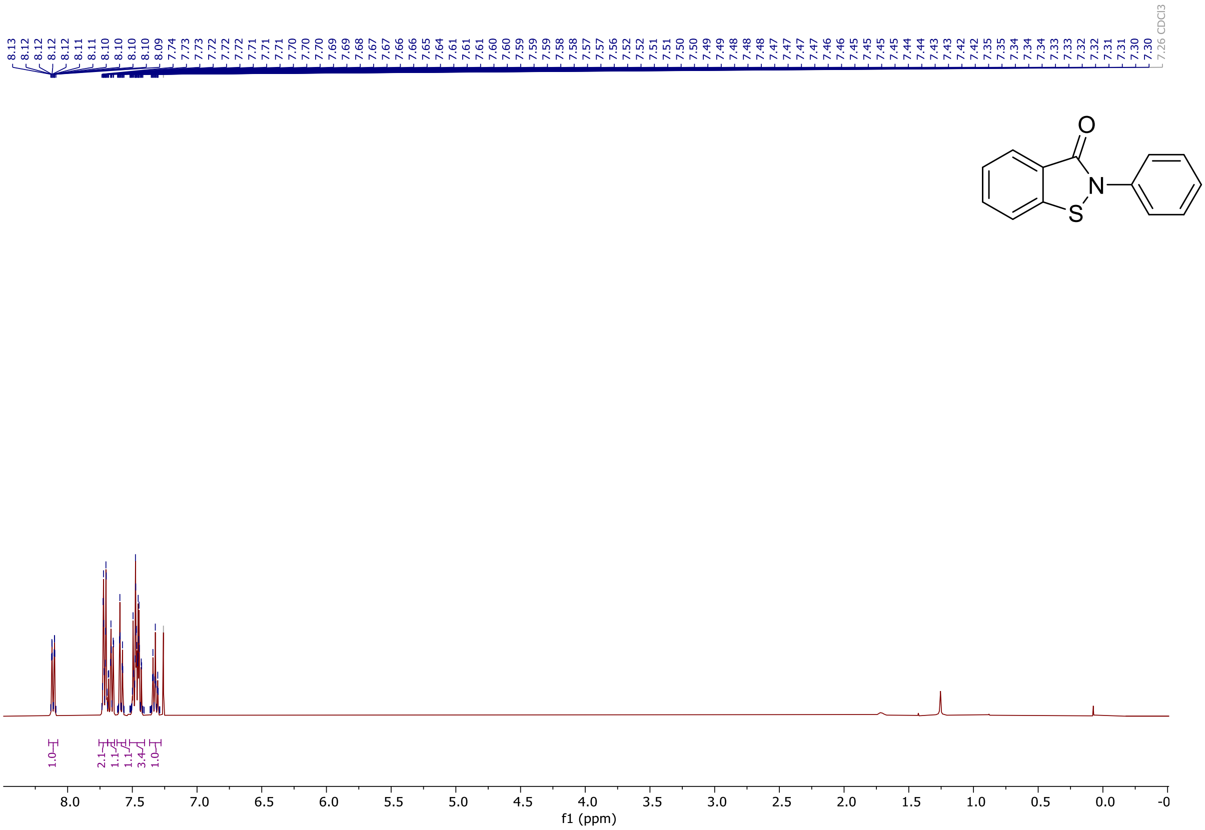

^{13}C NMR of **3a** (101 MHz, $CDCl_3$)

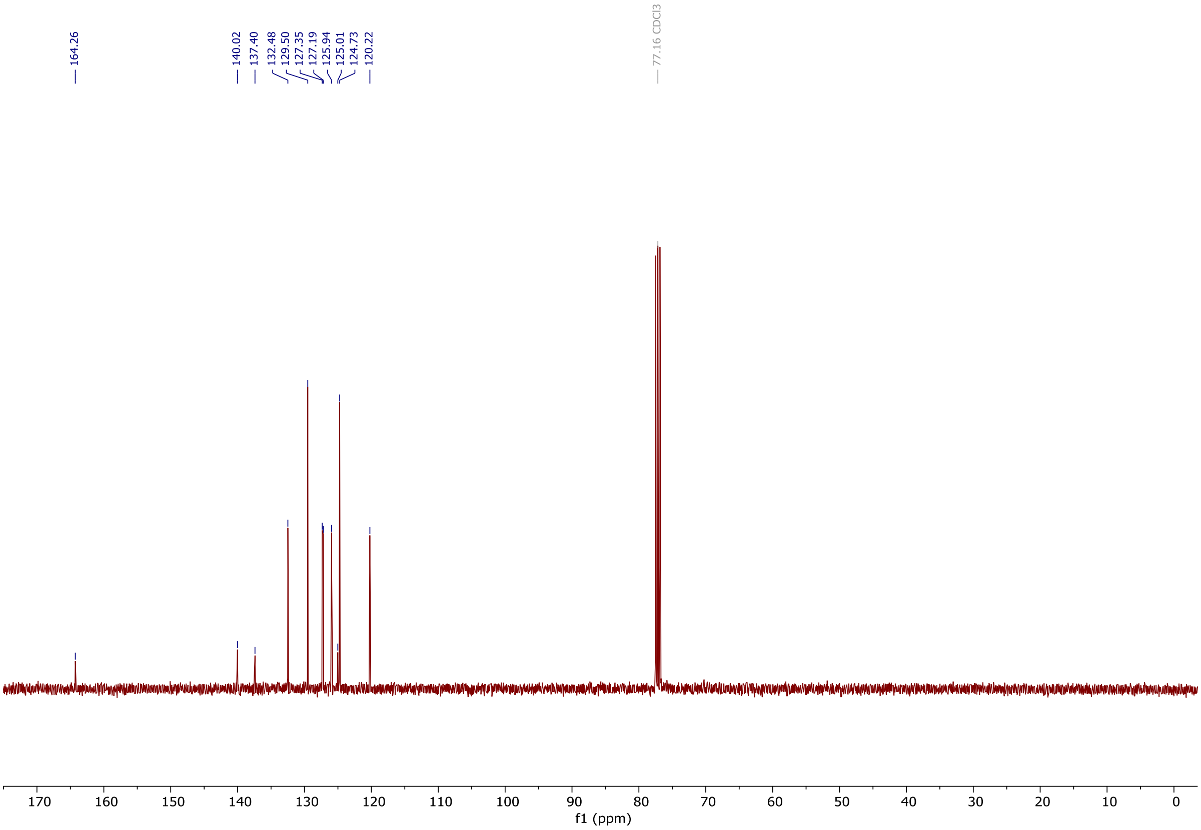

^{1}H NMR of **3b** (400 MHz, $CDCl_3$)

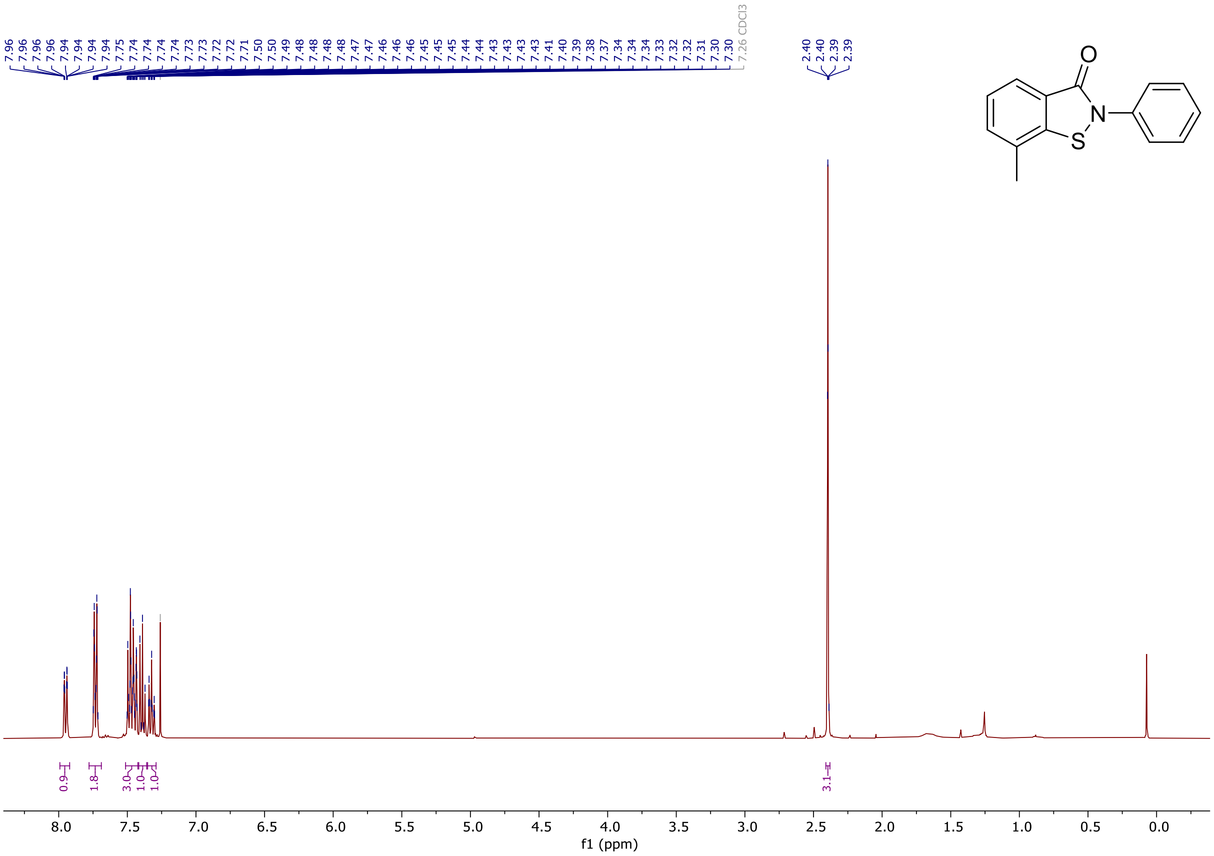

^{13}C NMR of **3b** (101 MHz, $CDCl_3$)

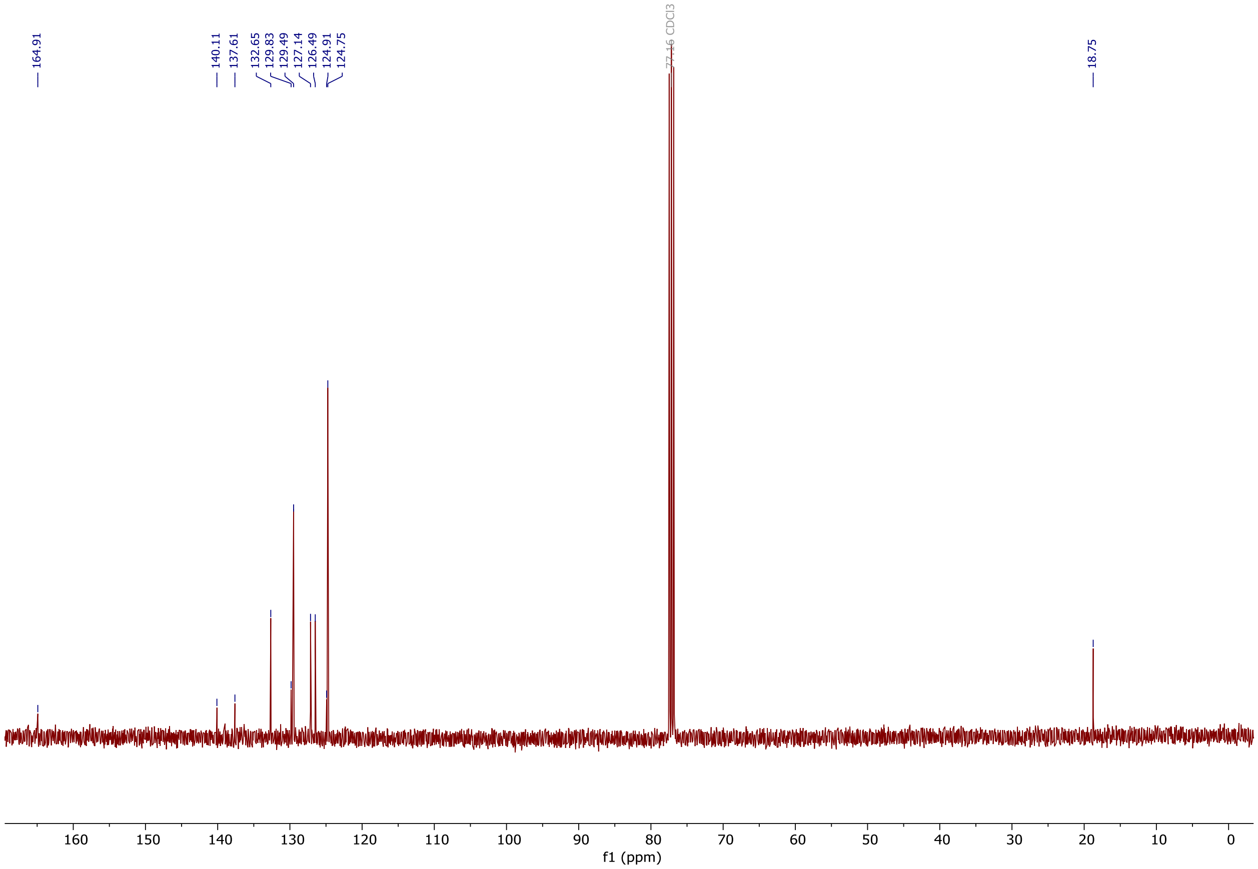

^{1}H NMR of **3c** (400 MHz, $CDCl_3$)

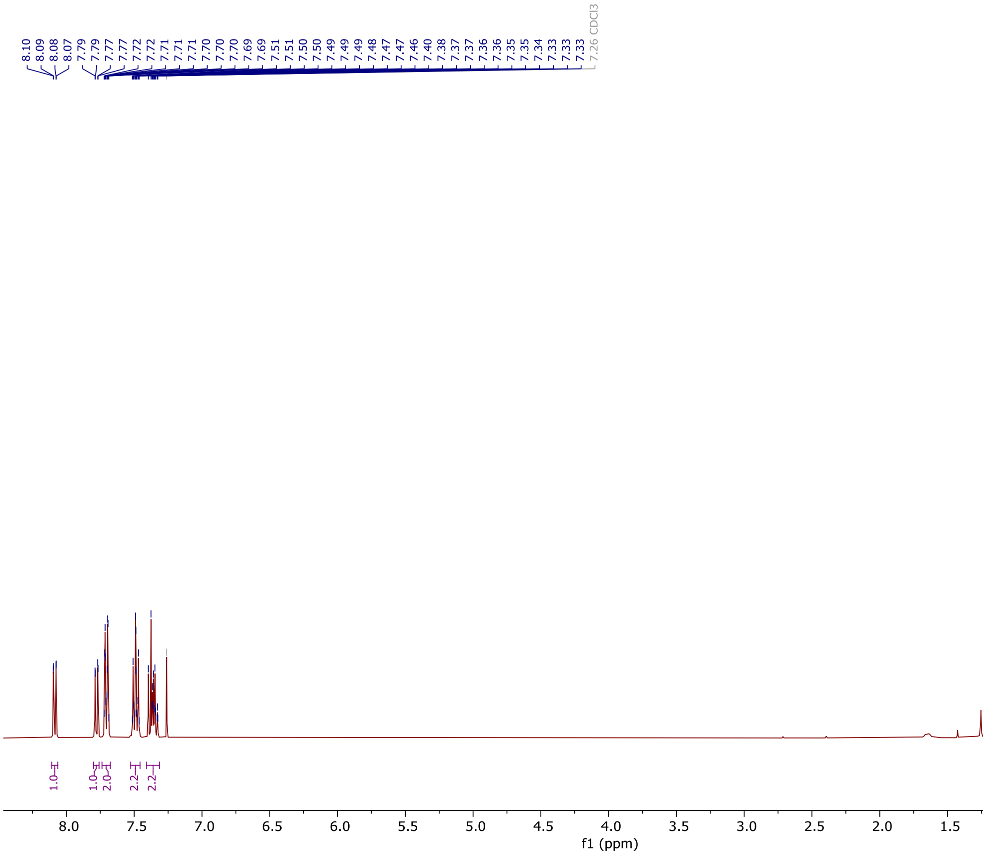

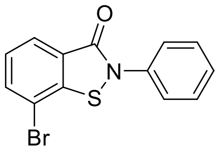

^{13}C NMR of **3c** (101 MHz, $CDCl_3$)

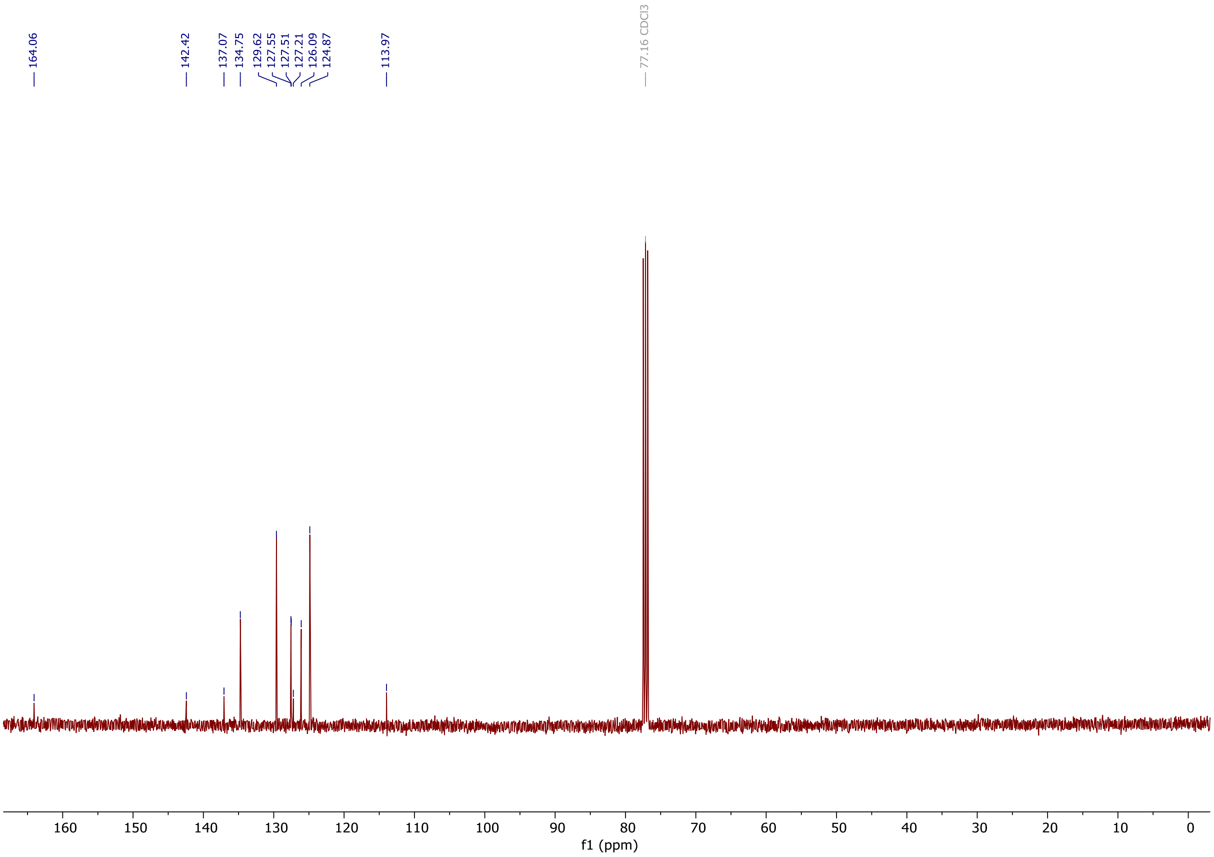

^{1}H NMR of **3d** (400 MHz, $CDCl_3$)

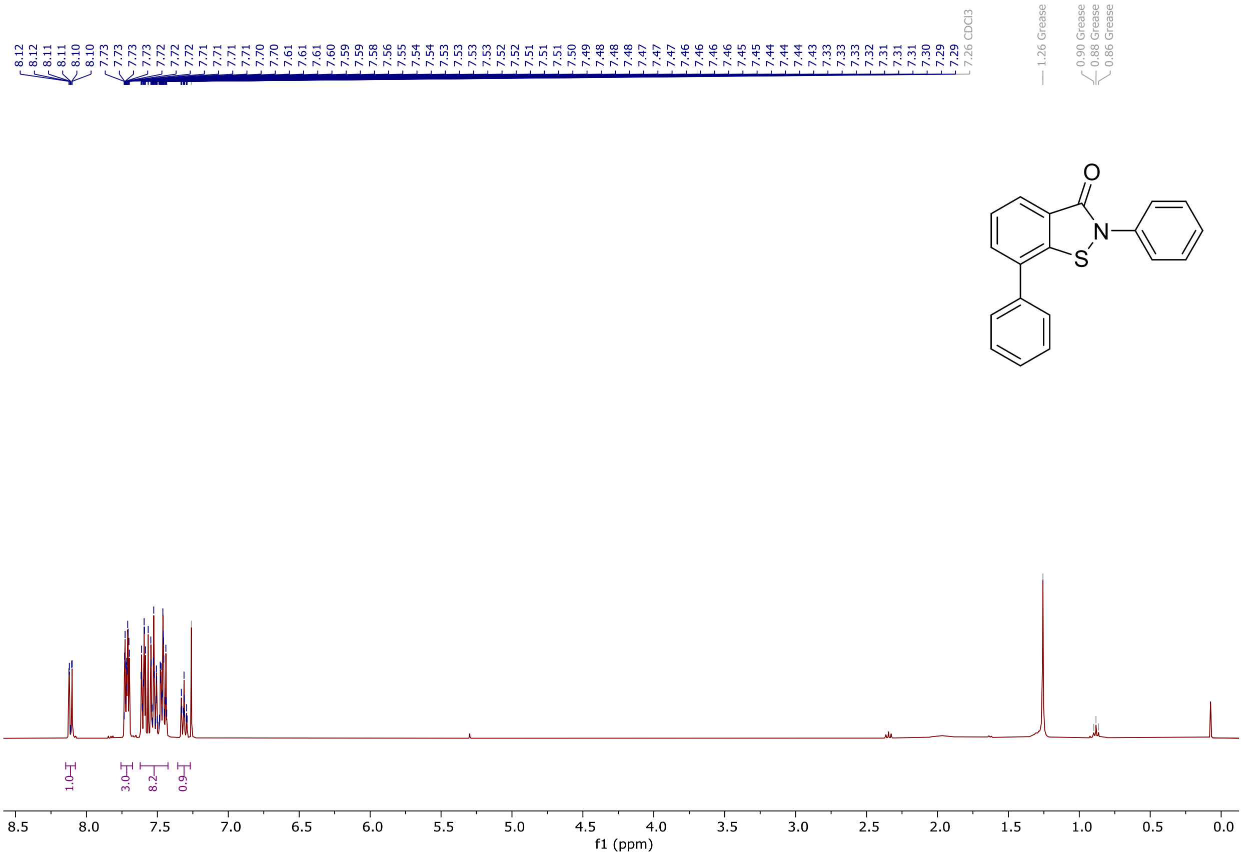

^{13}C NMR of **3d** (101 MHz, $CDCl_3$)

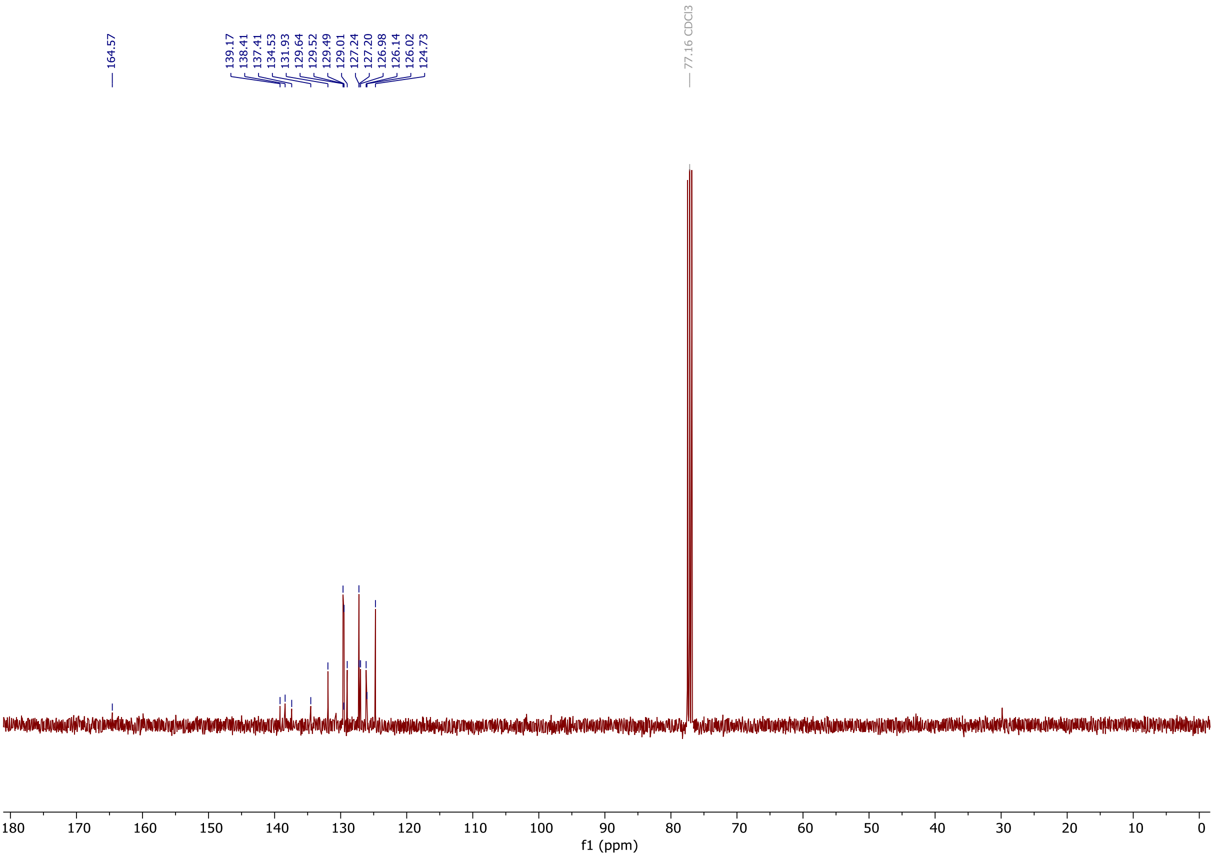

^{1}H NMR of **3e** (400 MHz, $CDCl_3$)

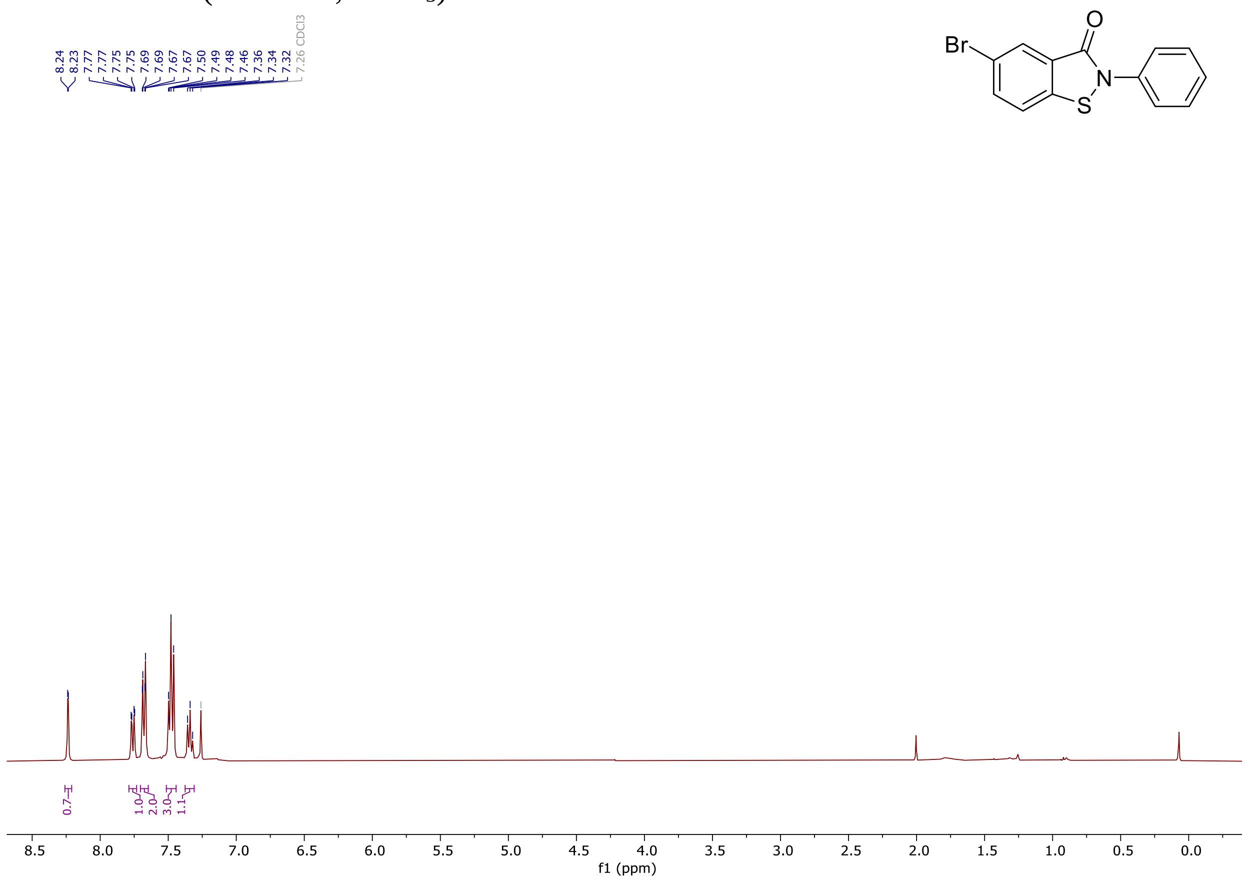

^{13}C NMR of **3e** (101 MHz, $CDCl_3$)

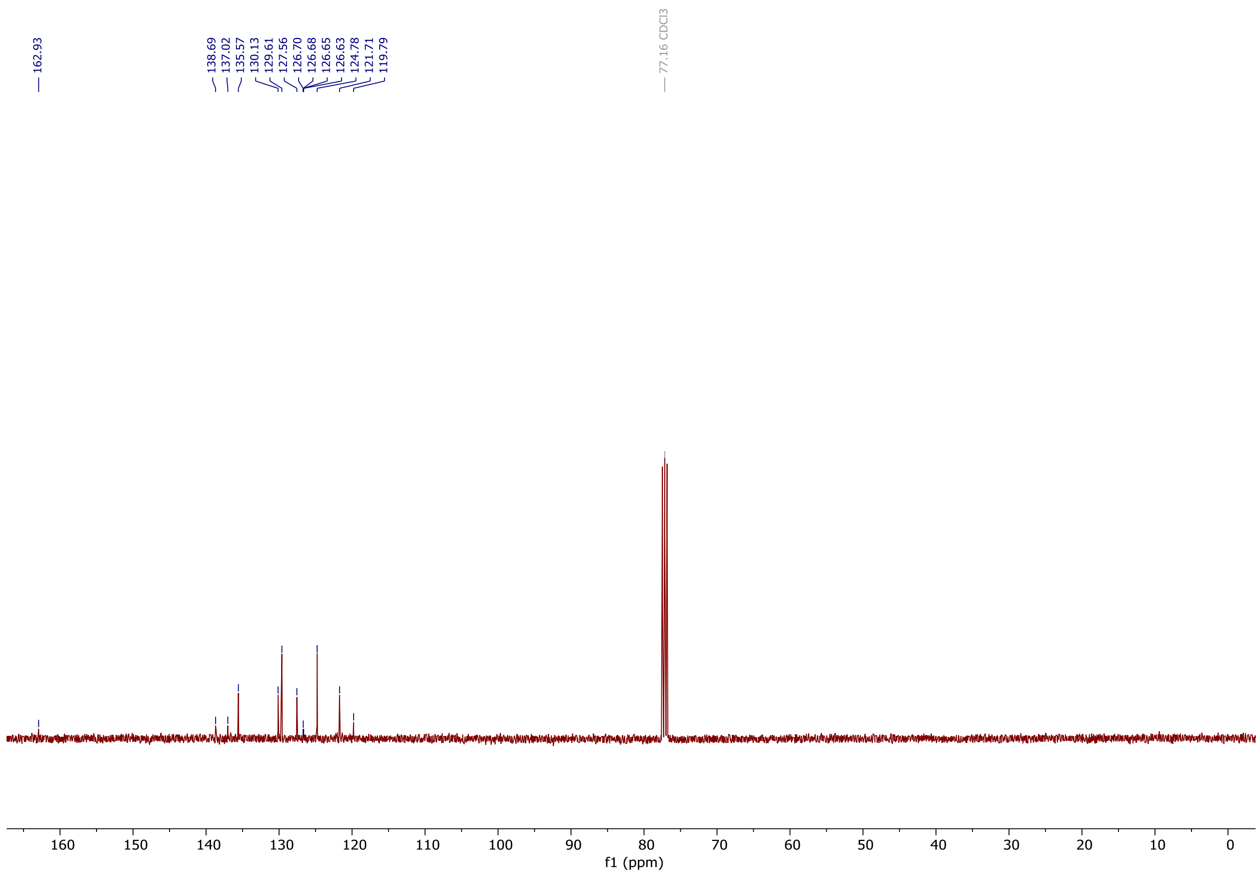

^{1}H NMR of **3f** (400 MHz, $CDCl_3$)

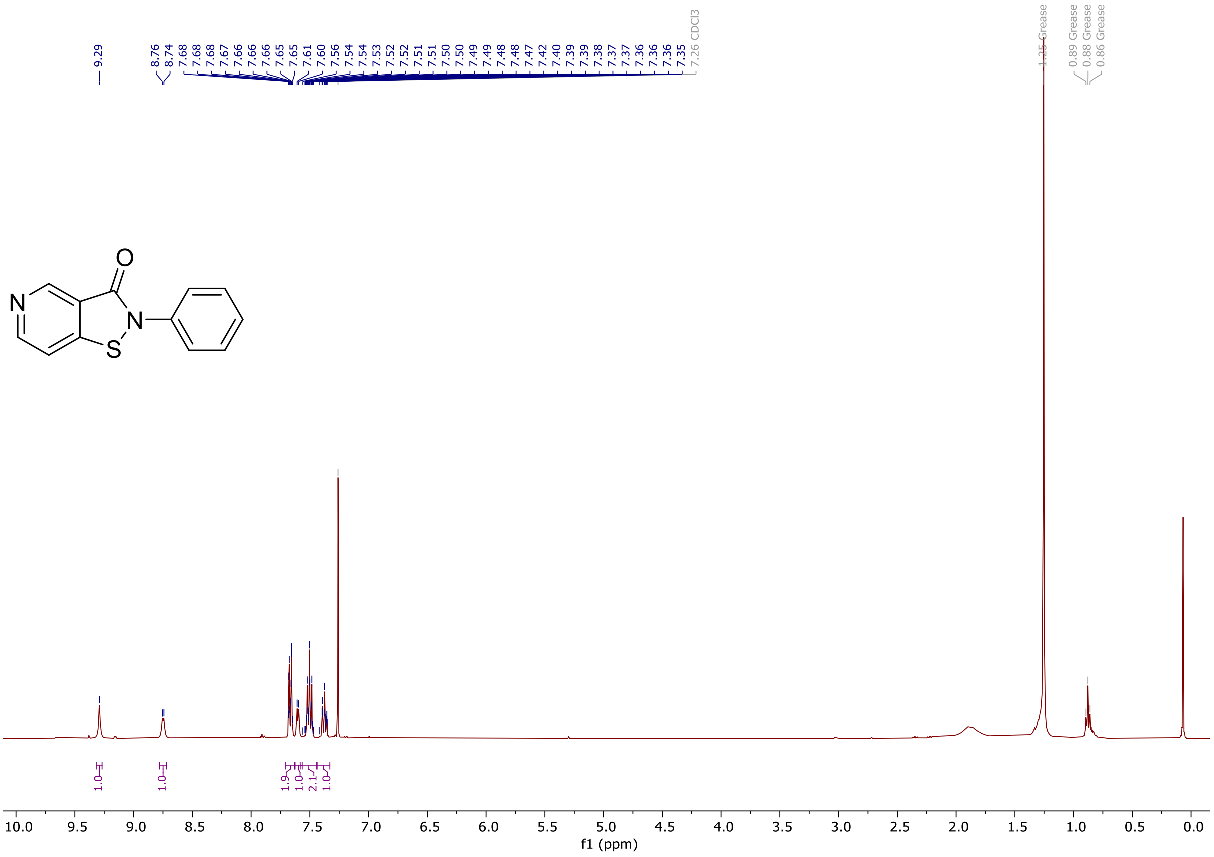

^{13}C NMR of **3f** (151 MHz, $CDCl_3$)

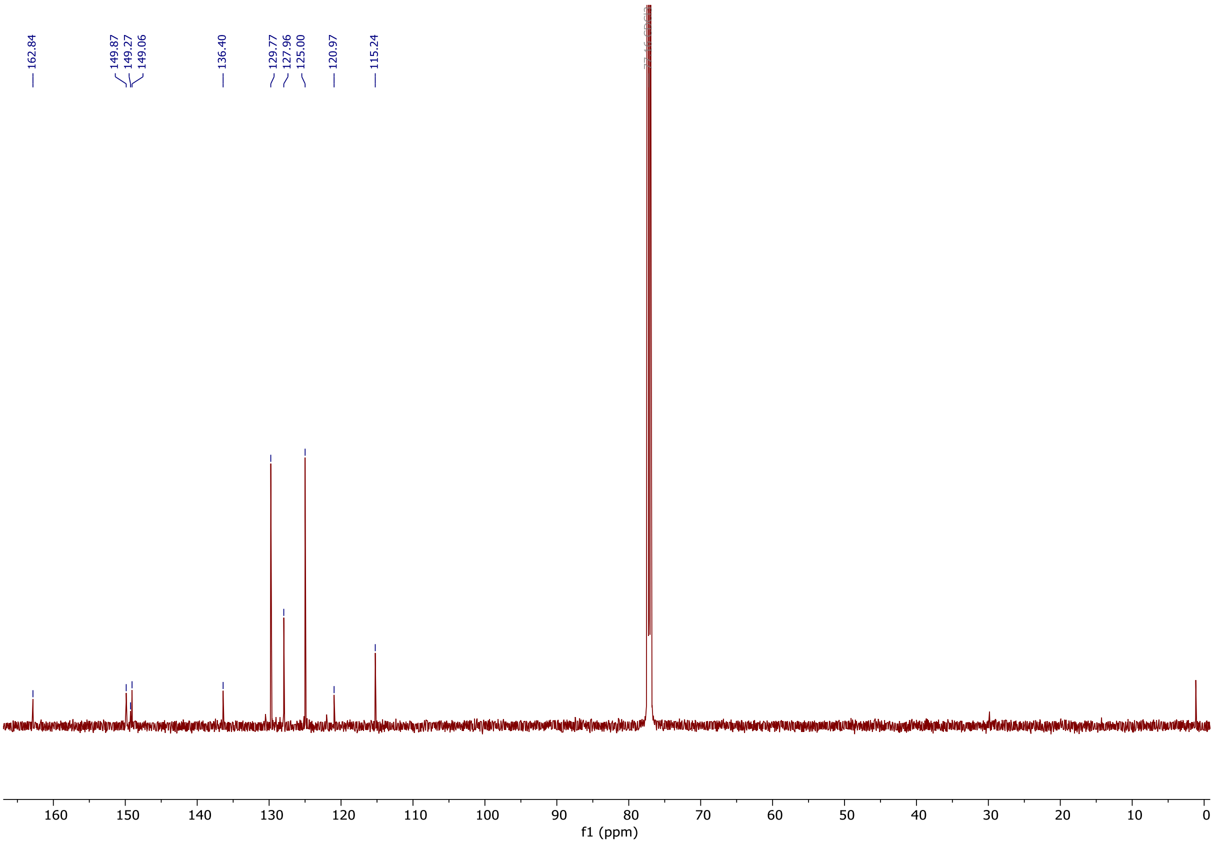

^{1}H NMR of **4a** (400 MHz, $CDCl_3$)

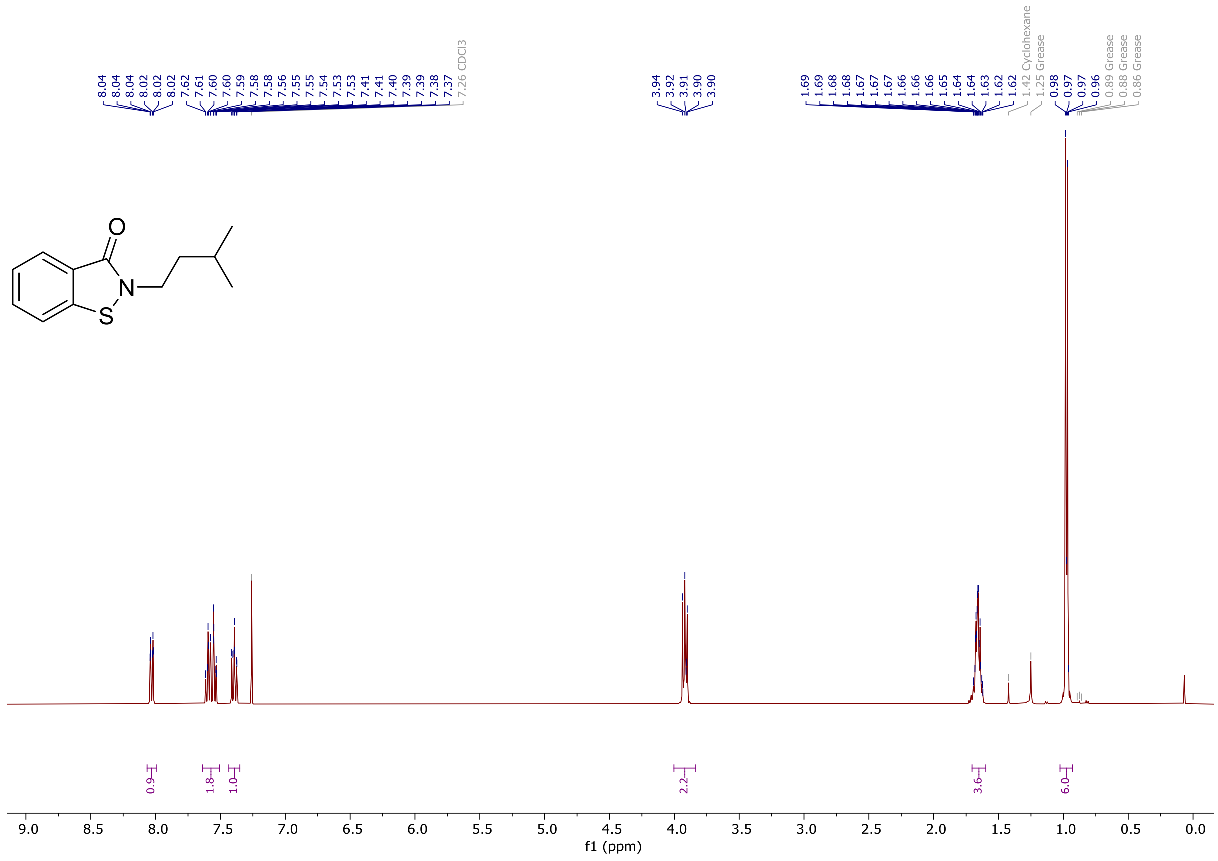

^{13}C NMR of **4a** (101 MHz, $CDCl_3$)

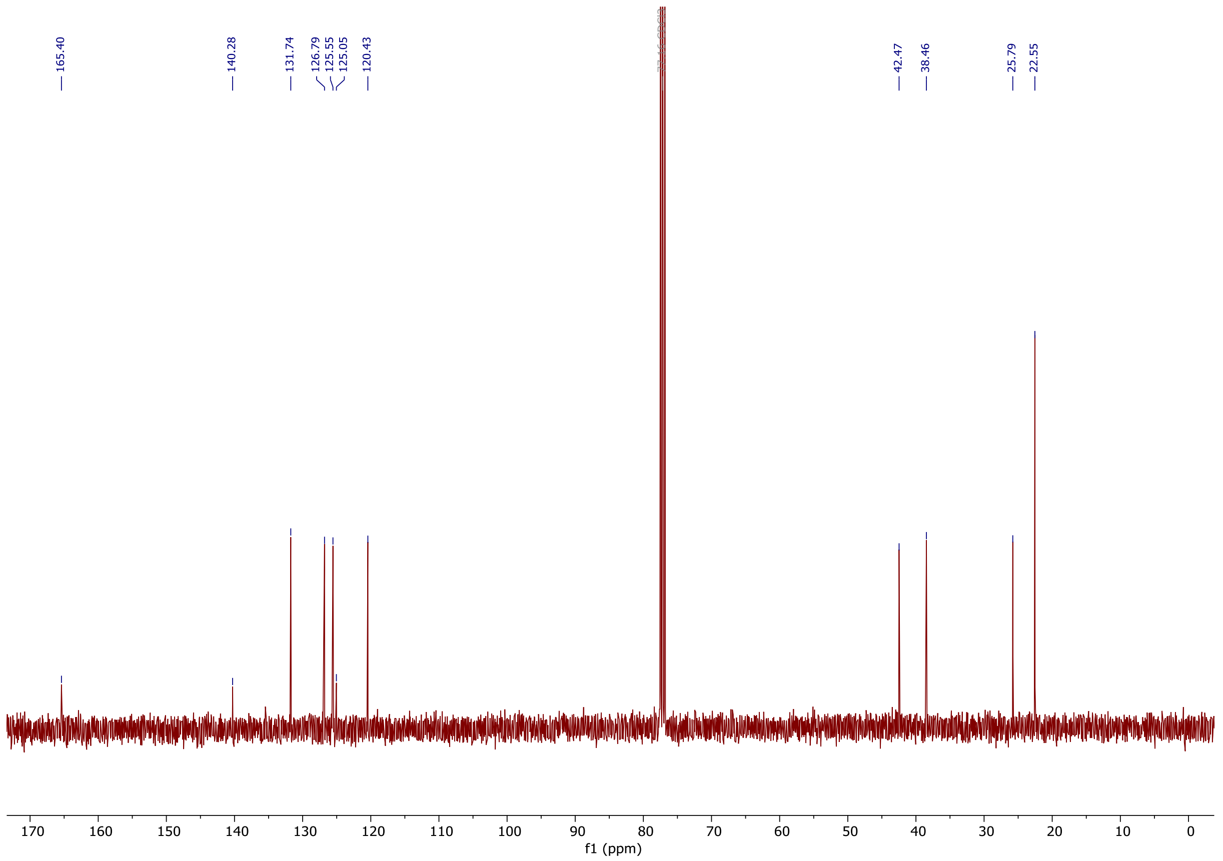

165.40
140.28
131.74
126.79
125.55
125.05
120.43
42.47
38.46
25.79
22.55
170
160
150
140
130
120
110
100
90
80
70
60
50
40
30
20
10
0
f1 (ppm)

^{1}H NMR of **4b** (400 MHz, $CDCl_3$)

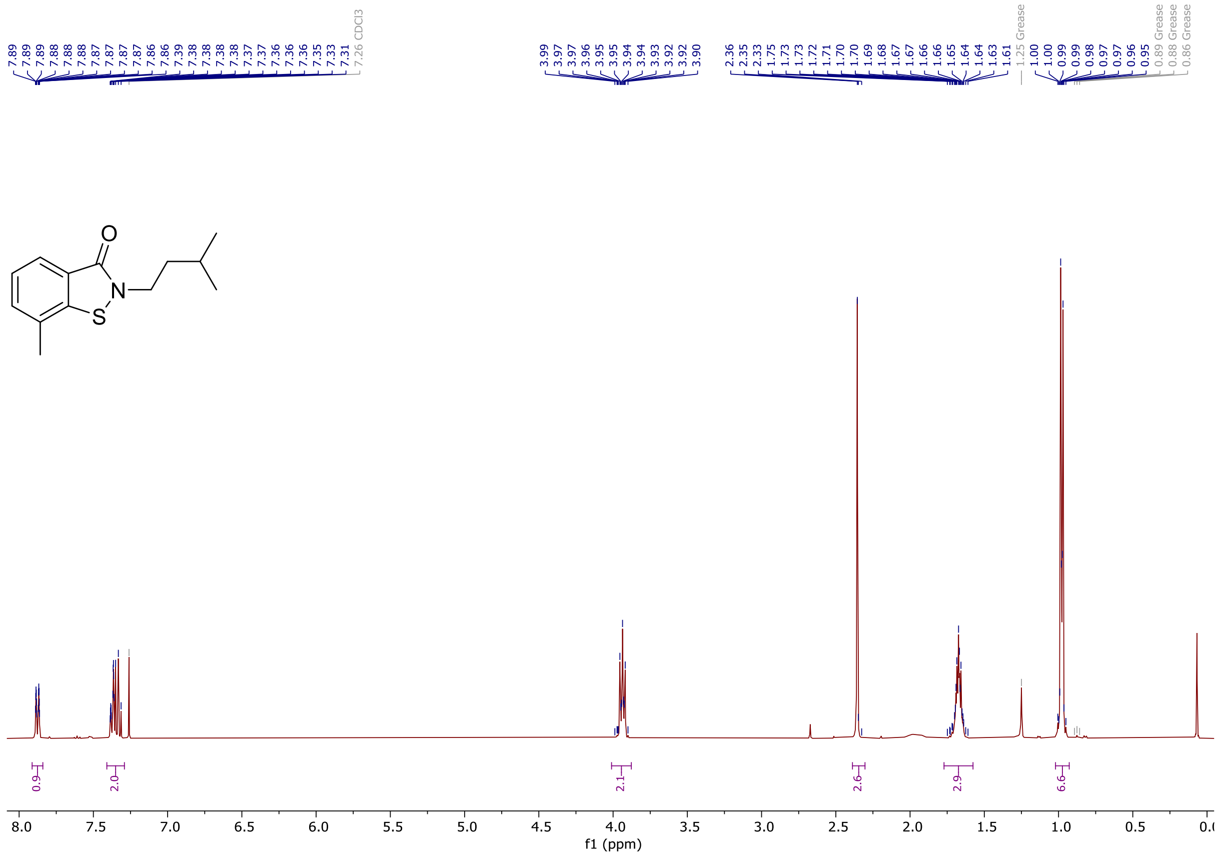

^{13}C NMR of **4b** (101 MHz, $CDCl_3$)

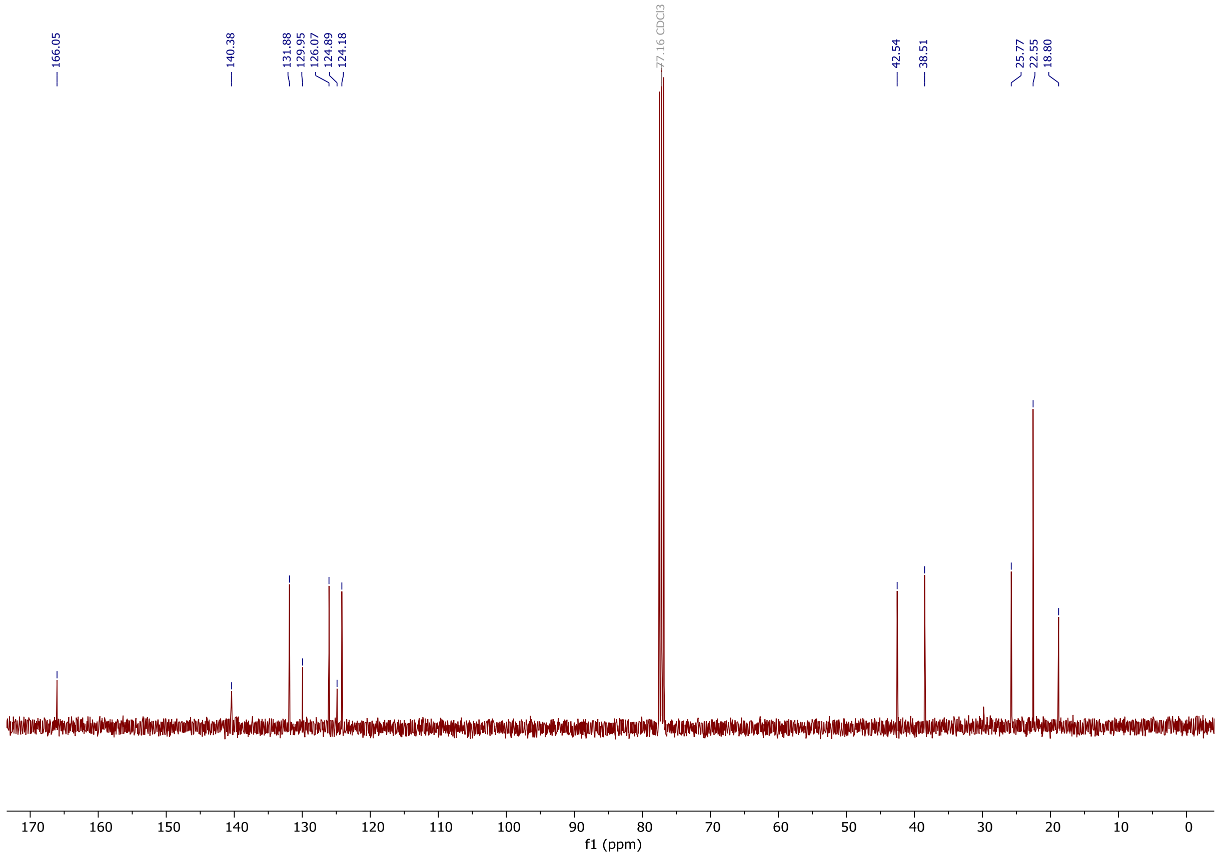
166.05
140.38
131.88
129.95
126.07
124.89
124.18
77.16 CDCl3
42.54
38.51
25.77
22.55
18.80
170 160 150 140 130 120 110 100 90 80 70 60 50 40 30 20 10 0
f1 (ppm)

^{1}H NMR of **4c** (400 MHz, C_2D_6OS)

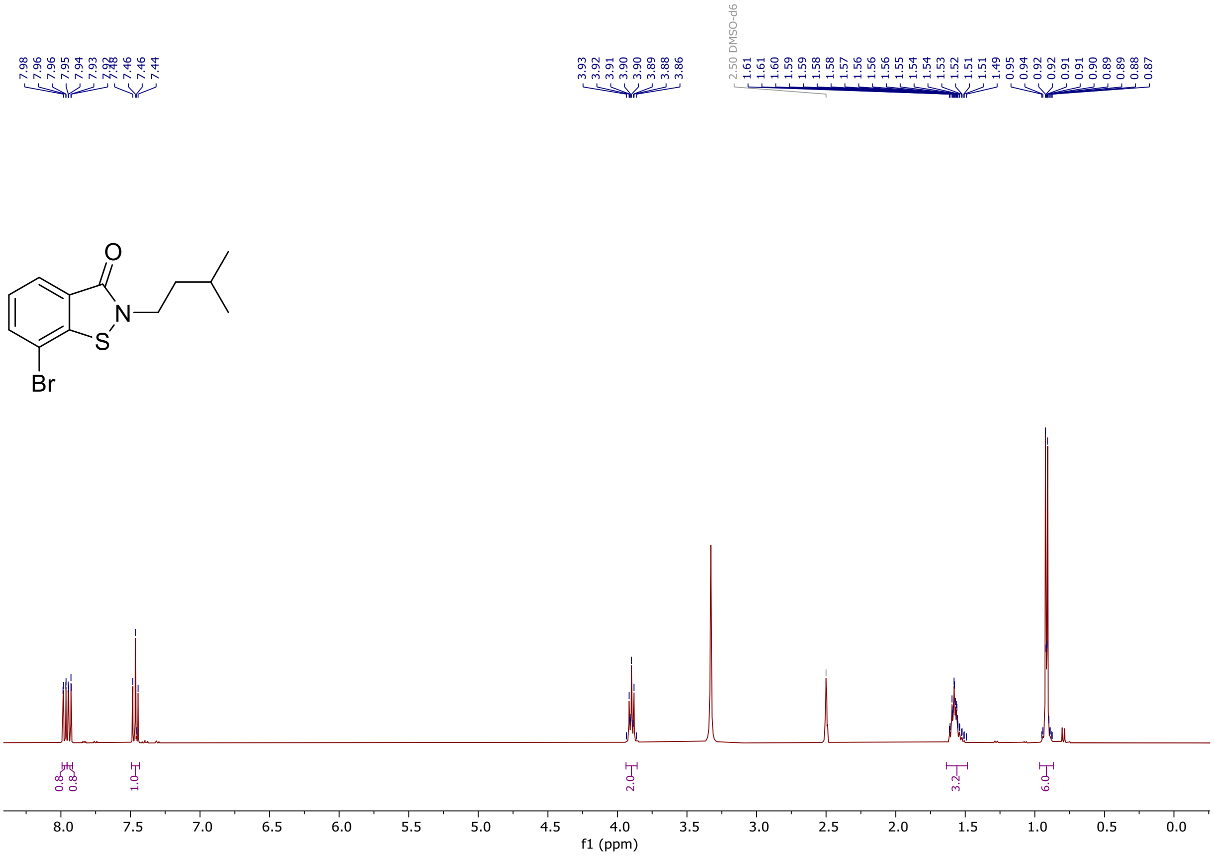

^{13}C NMR of **4c** (101 MHz, C_2D_6OS)

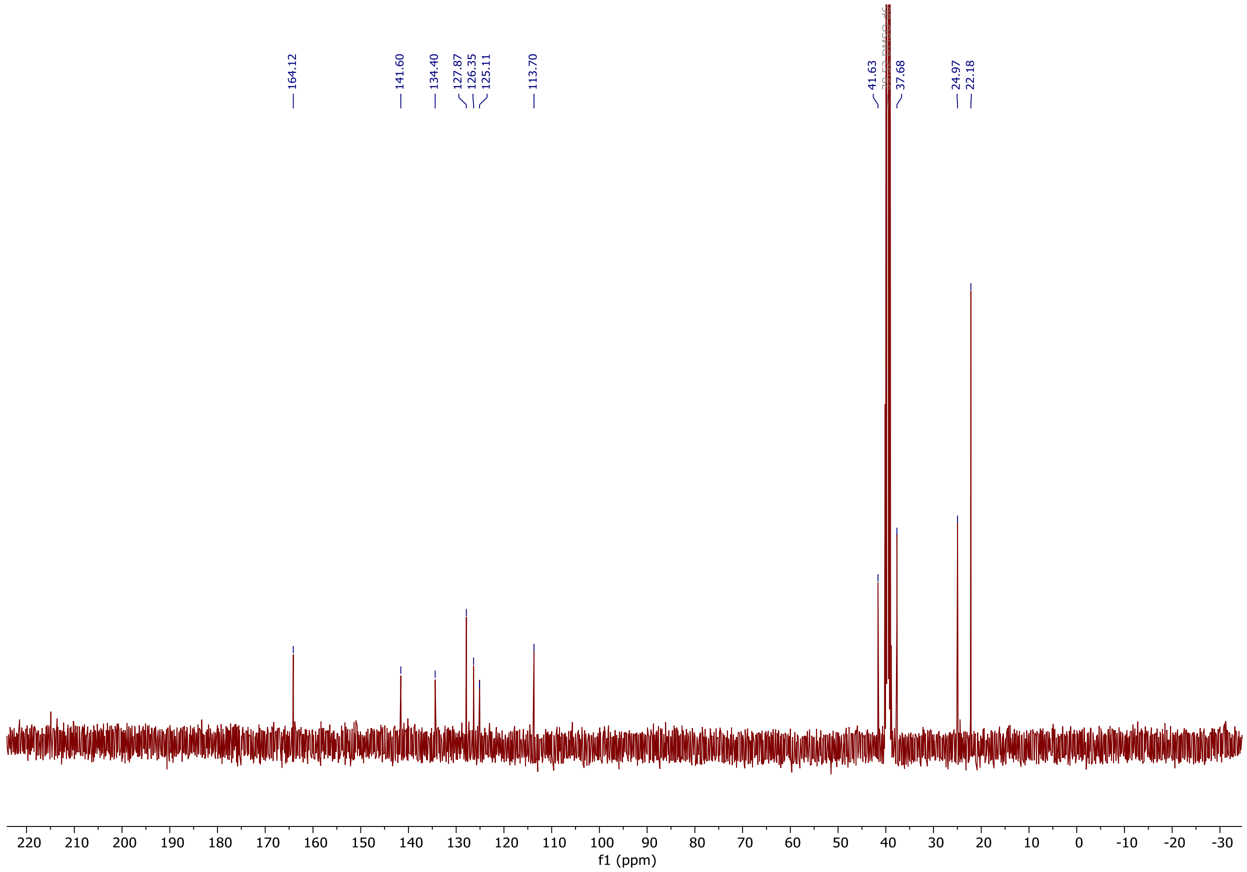

^{1}H NMR of **4d** (400 MHz, $CDCl_3$)

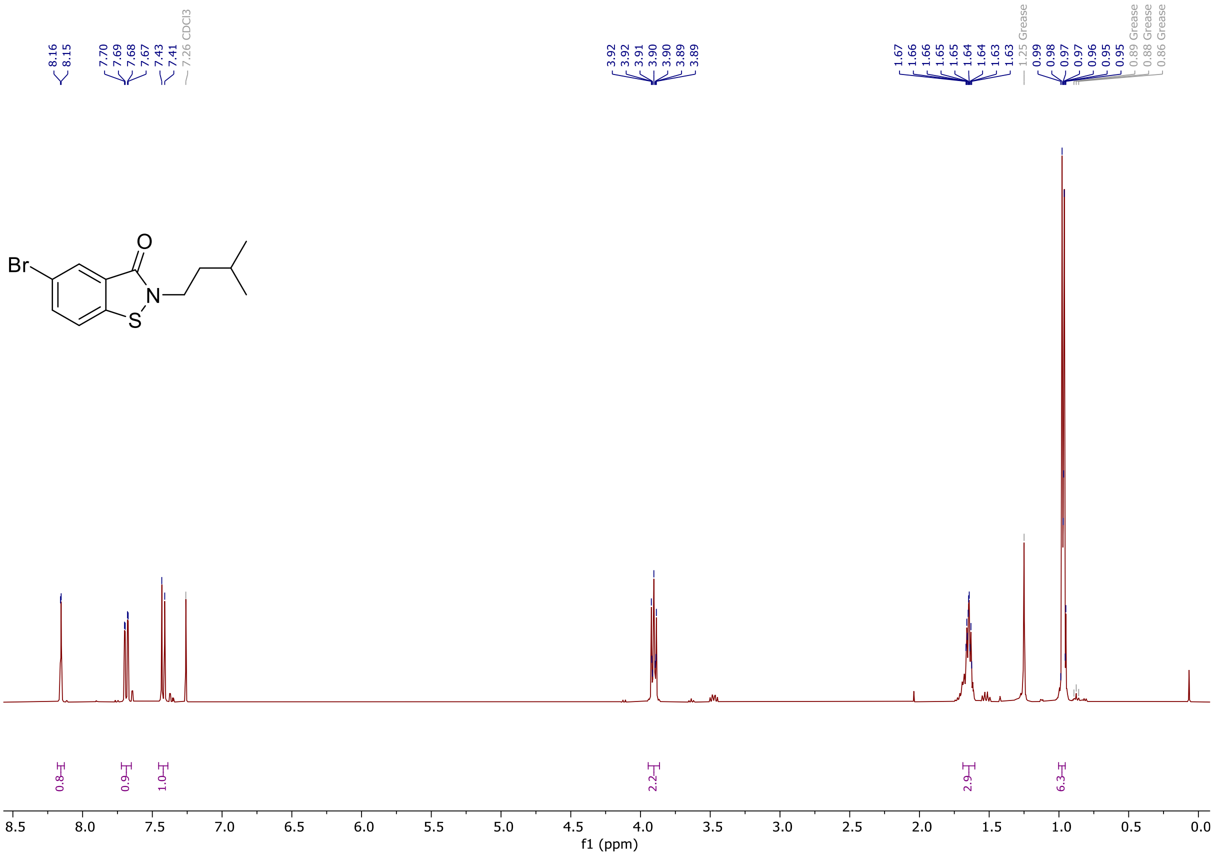

^{13}C NMR of **4d** (101 MHz, $CDCl_3$)

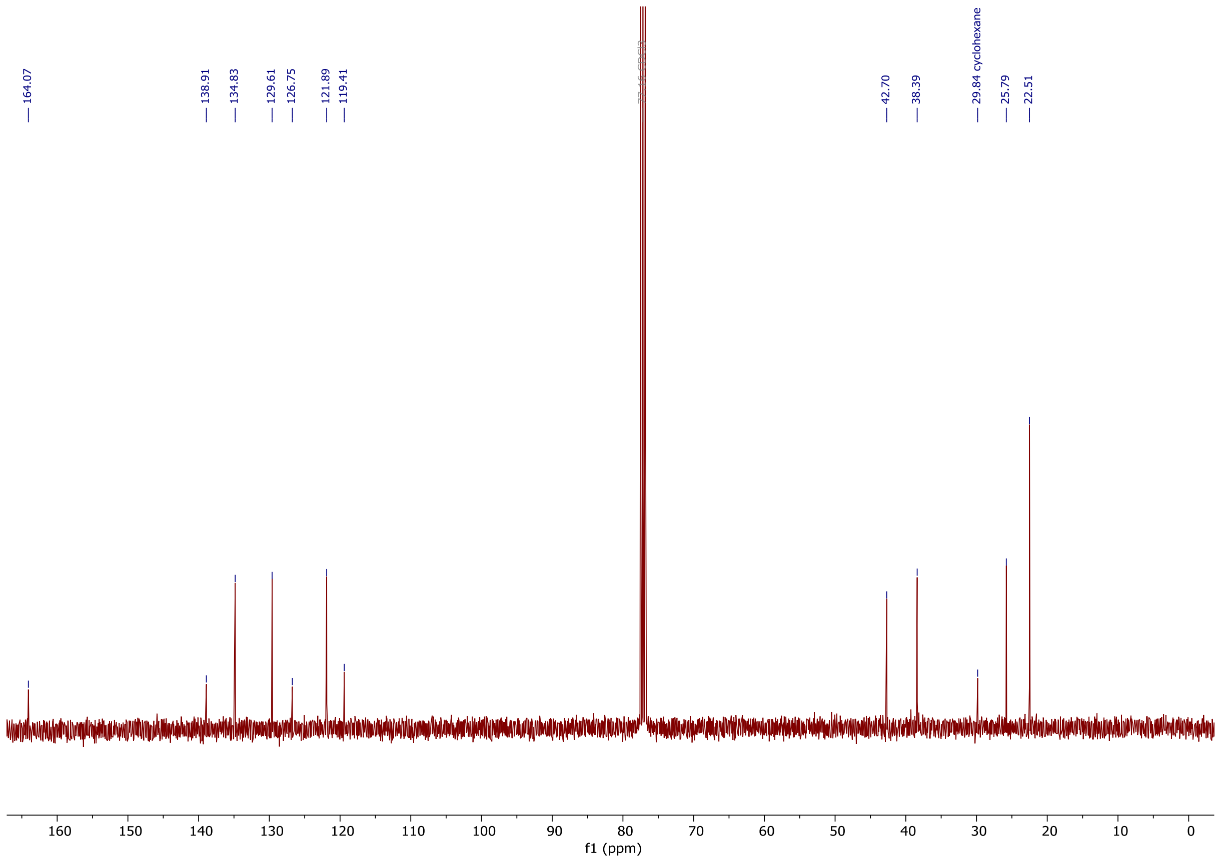
164.07
138.91
134.83
129.61
126.75
121.89
119.41
42.70
38.39
29.84 cyclohexane
25.79
22.51
160
150
140
130
120
110
100
90
80
70
60
50
40
30
20
10
0
f1 (ppm)

^{1}H NMR of **5** (400 MHz, $CDCl_3$)

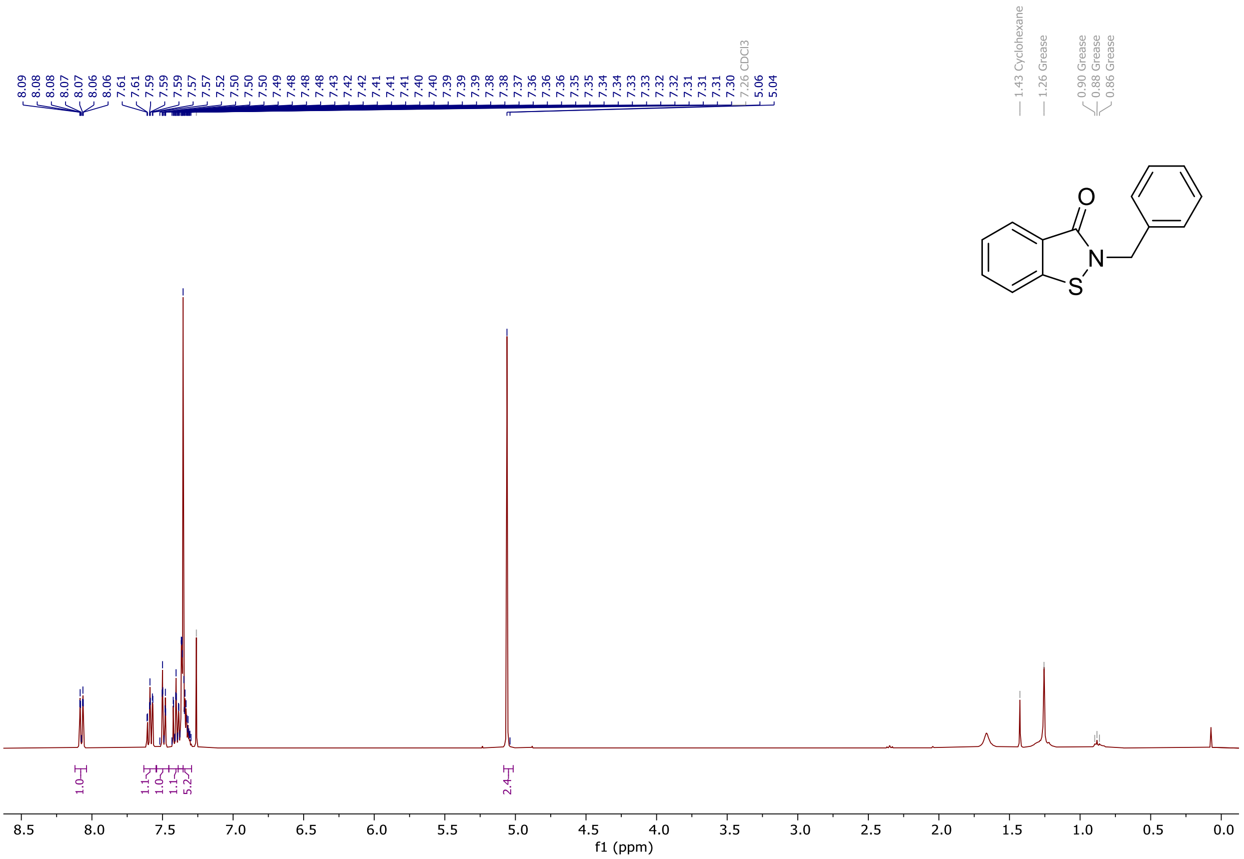

^{13}C NMR of **5** (101 MHz, $CDCl_3$)

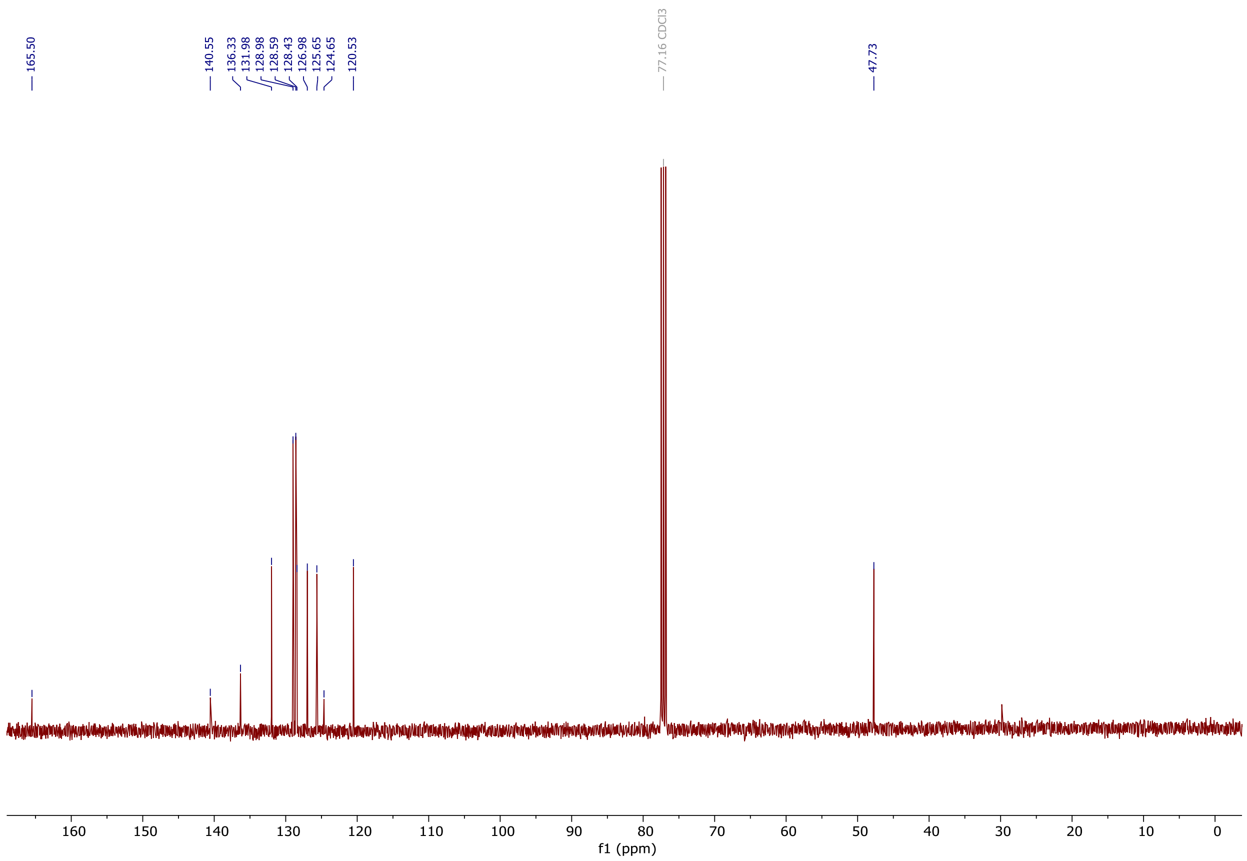
165.50
140.55
136.33
131.98
128.98
128.59
128.43
126.98
125.65
124.65
120.53
77.16 CDCl3
47.73
160
150
140
130
120
110
100
90
80
70
60
50
40
30
20
10
0
f1 (ppm)

^{1}H NMR of **6** (400 MHz, $CDCl_3$)

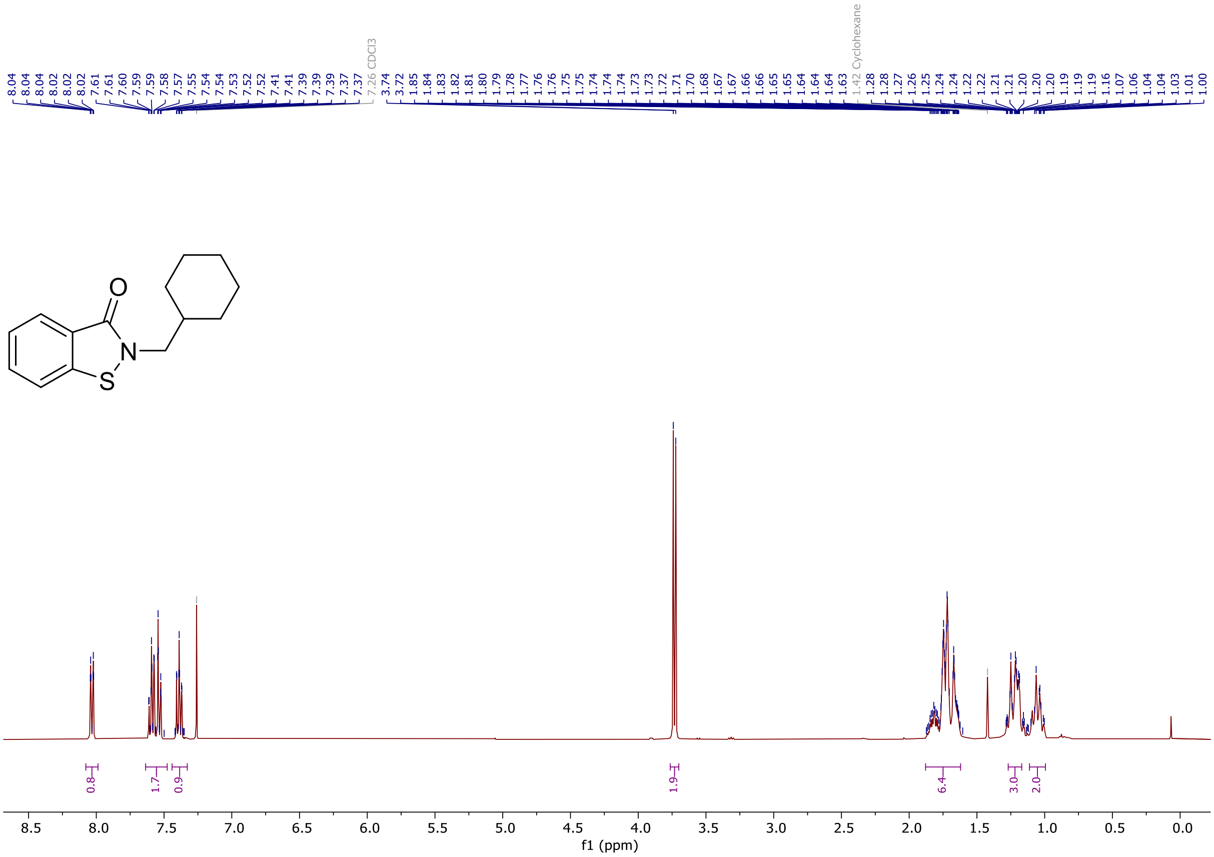

^{13}C NMR of **6** (101 MHz, $CDCl_3$)

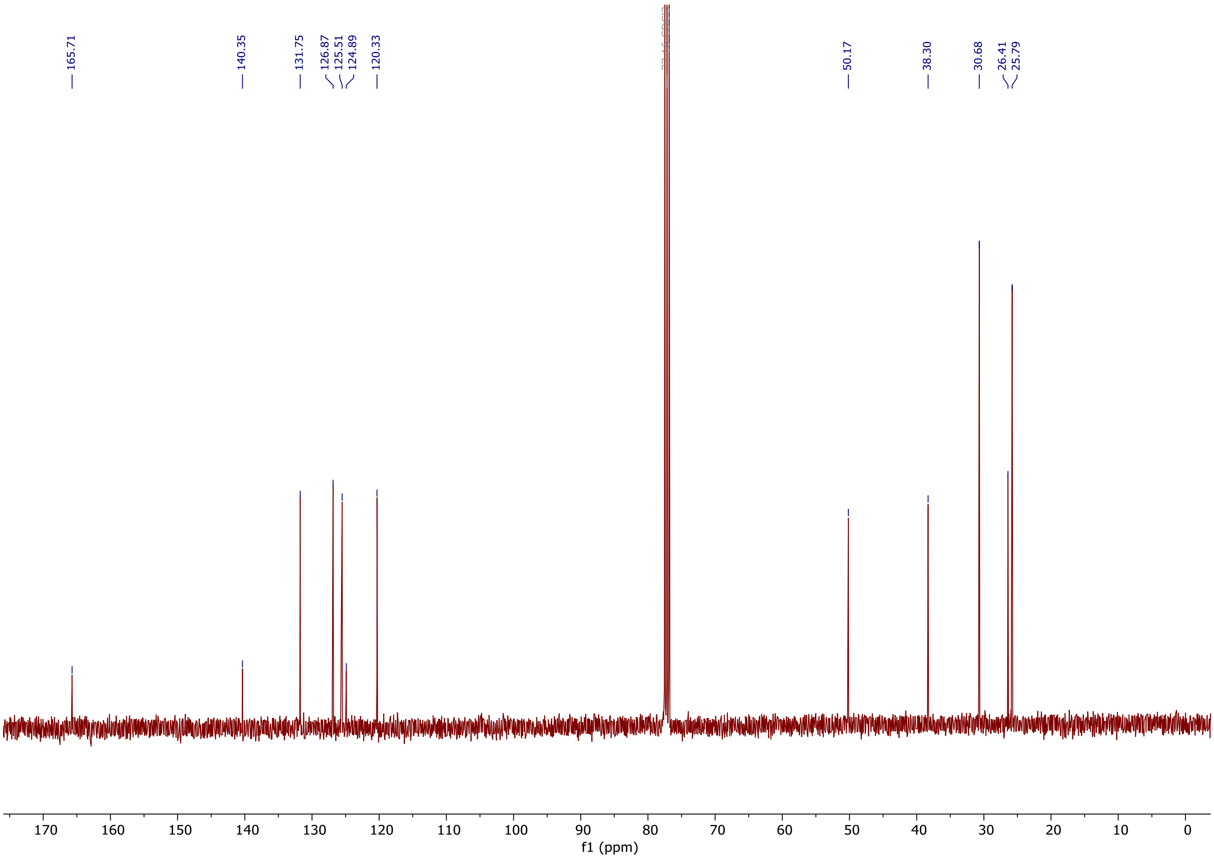

^{1}H NMR of **7** (400 MHz, $CDCl_3$)

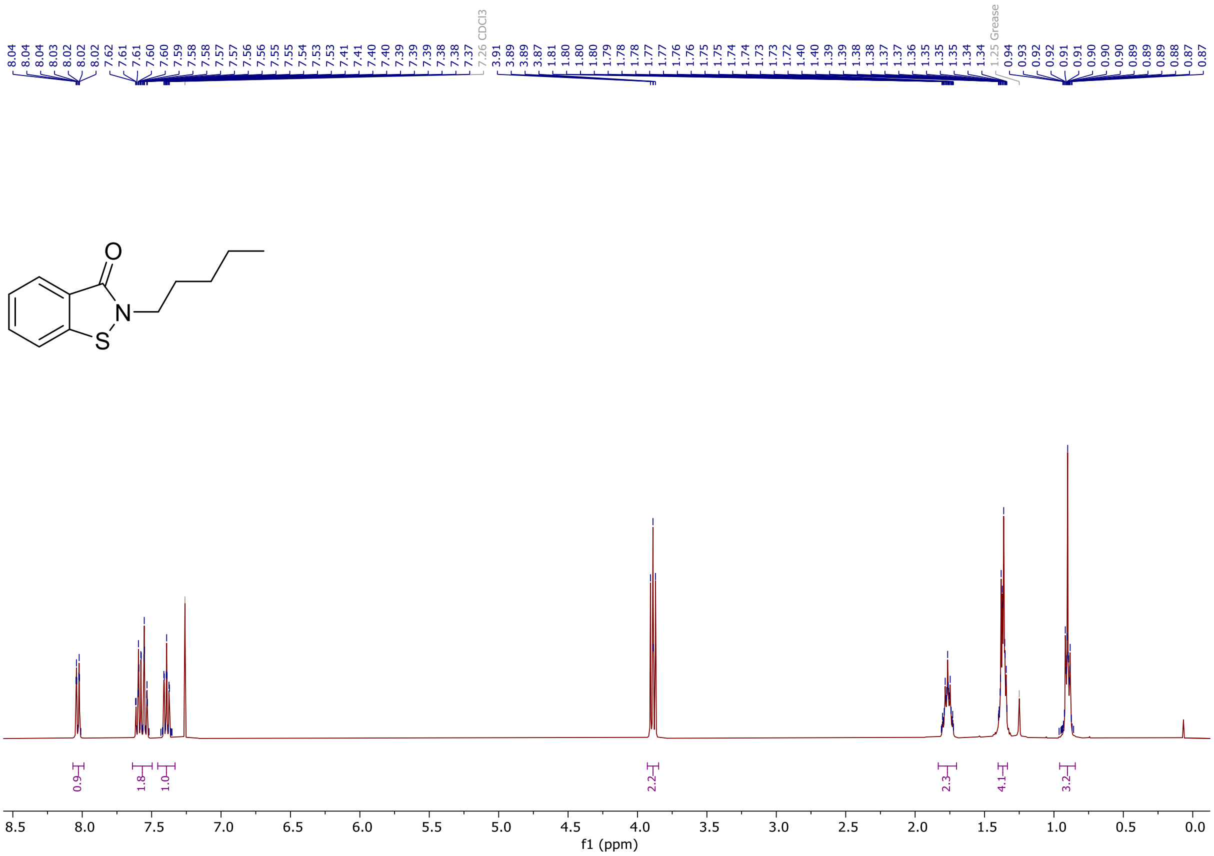

^{13}C NMR of **7** (101 MHz, $CDCl_3$)

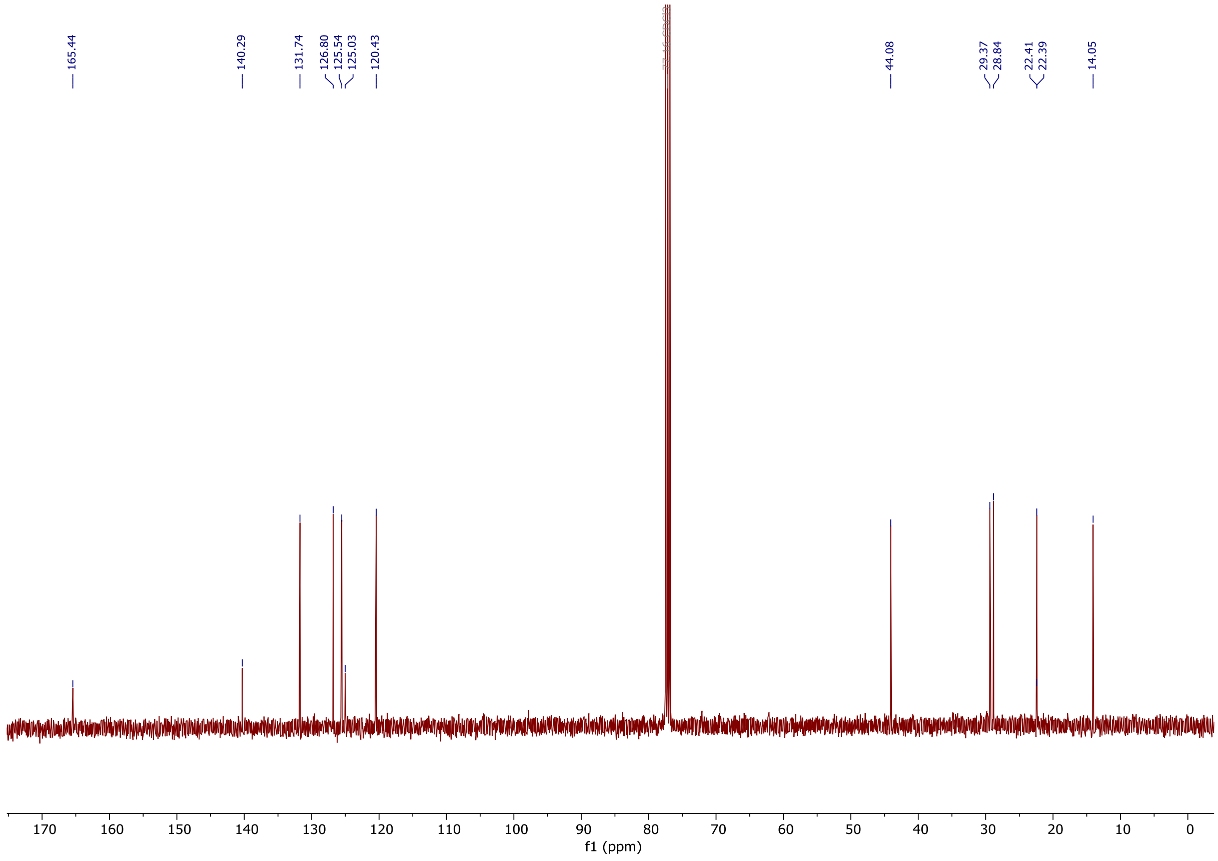

^{1}H NMR of **8** (400 MHz, $CDCl_3$)

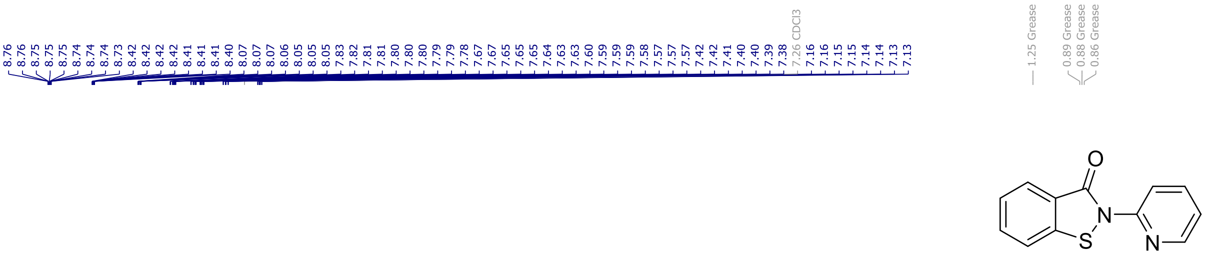

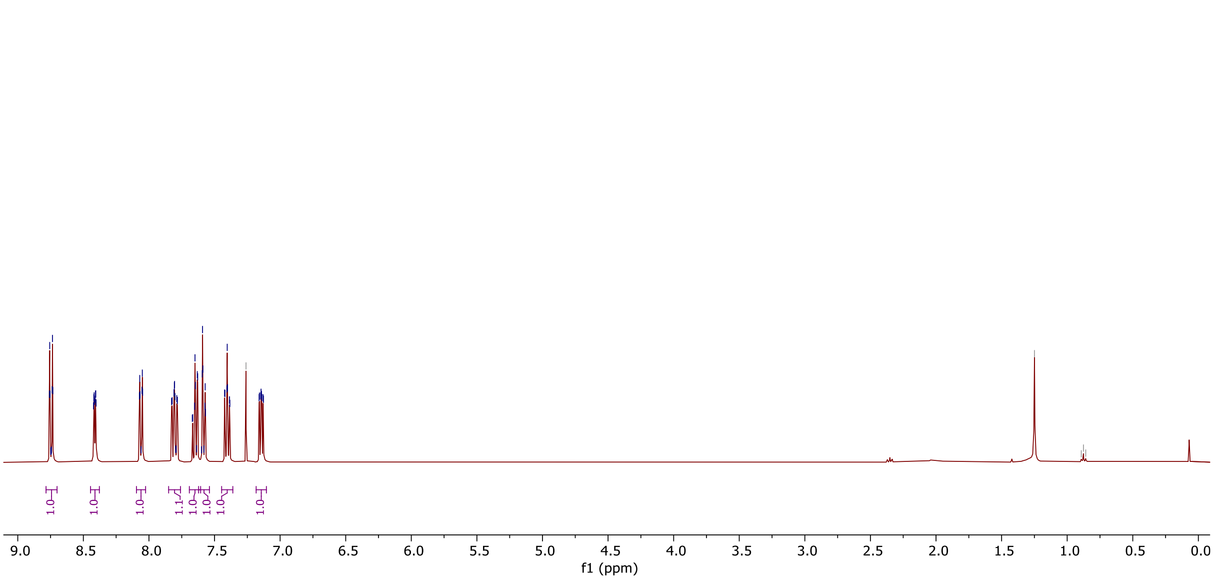

^{13}C NMR of **8** (101 MHz, $CDCl_3$)

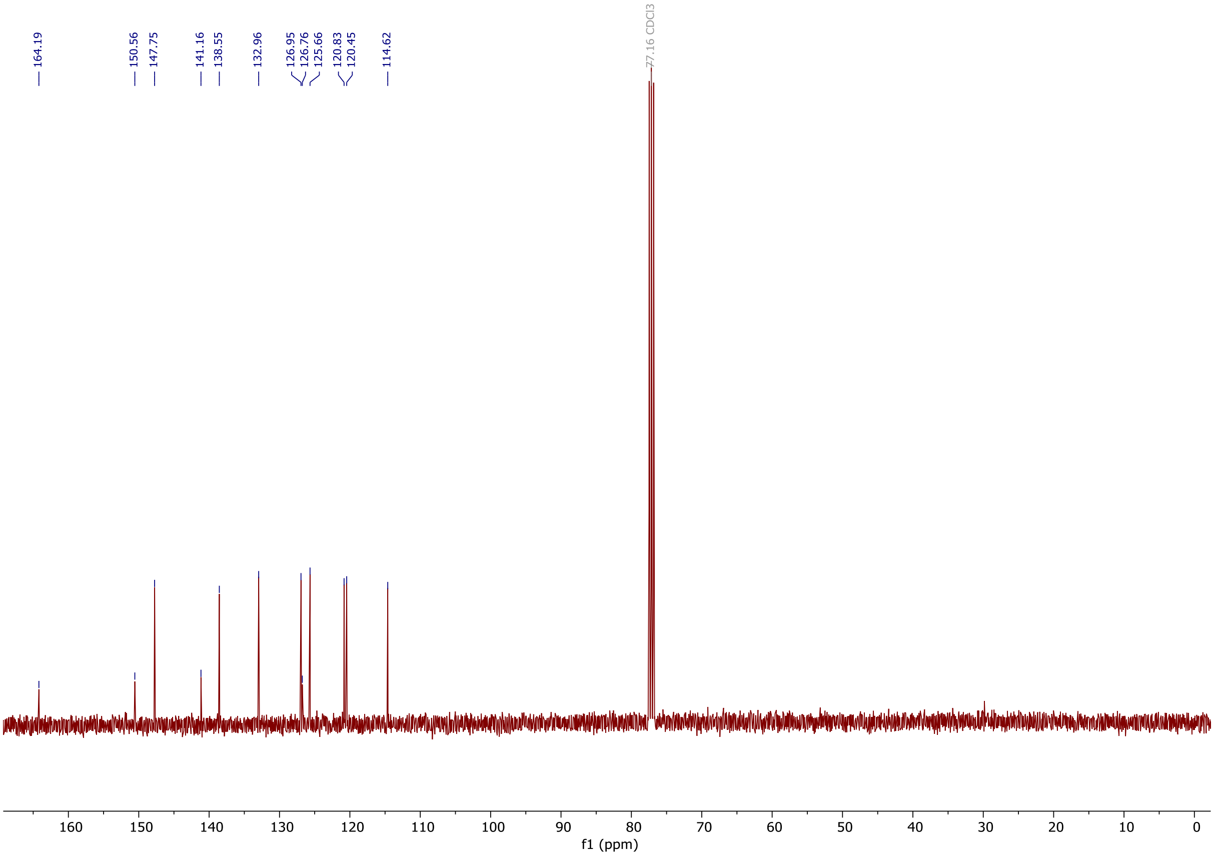

^{1}H NMR of **9** (400 MHz, $CDCl_3$)

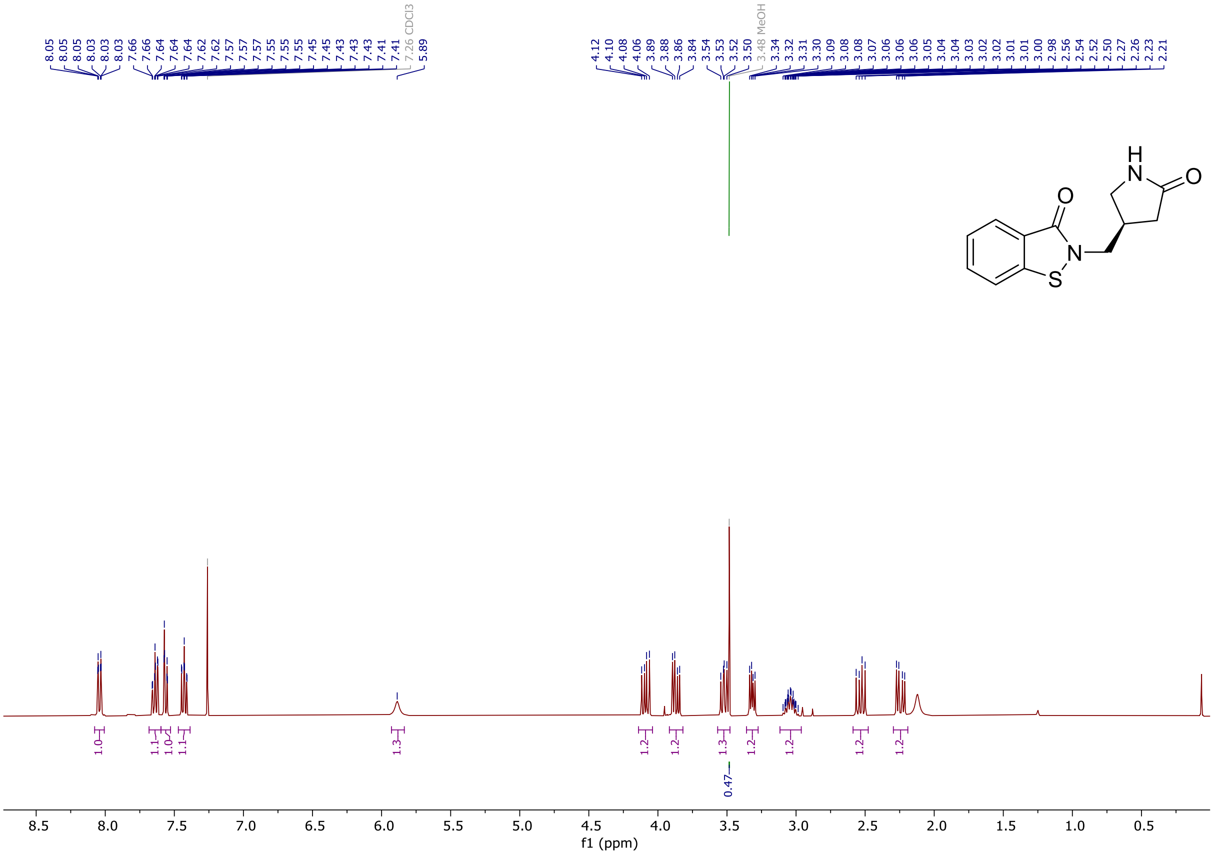

^{13}C NMR of **9** (101 MHz, $CDCl_3$)

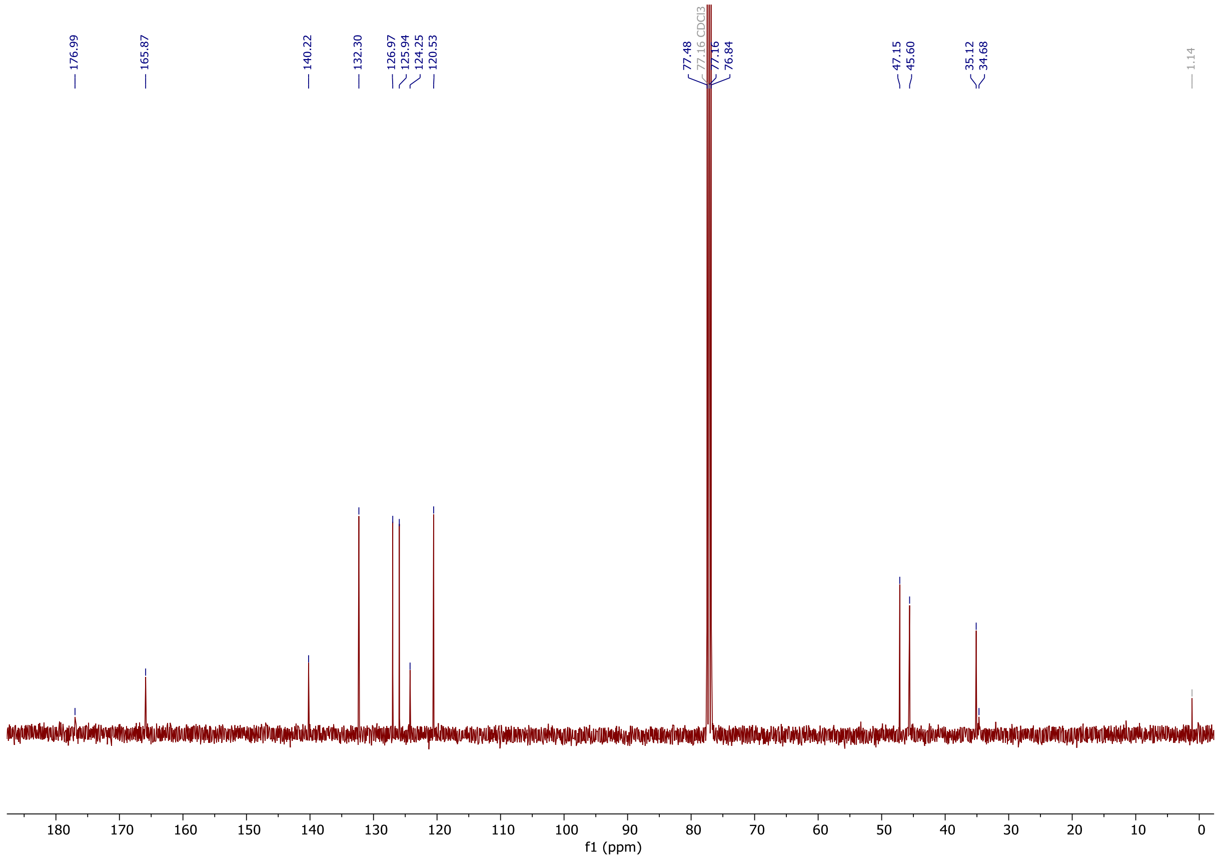

^{1}H NMR of **10** (400 MHz, $CDCl_3$)

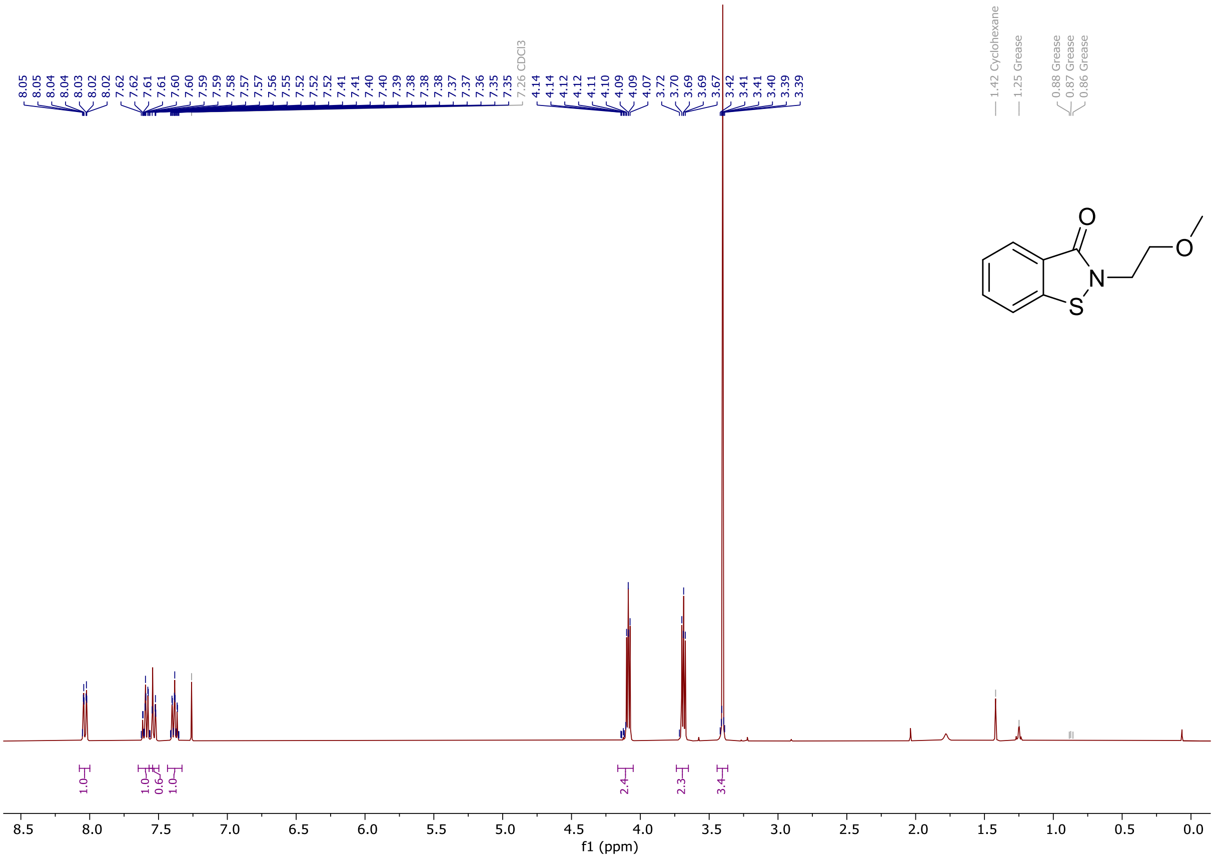

^{13}C NMR of **10** (101 MHz, $CDCl_3$)

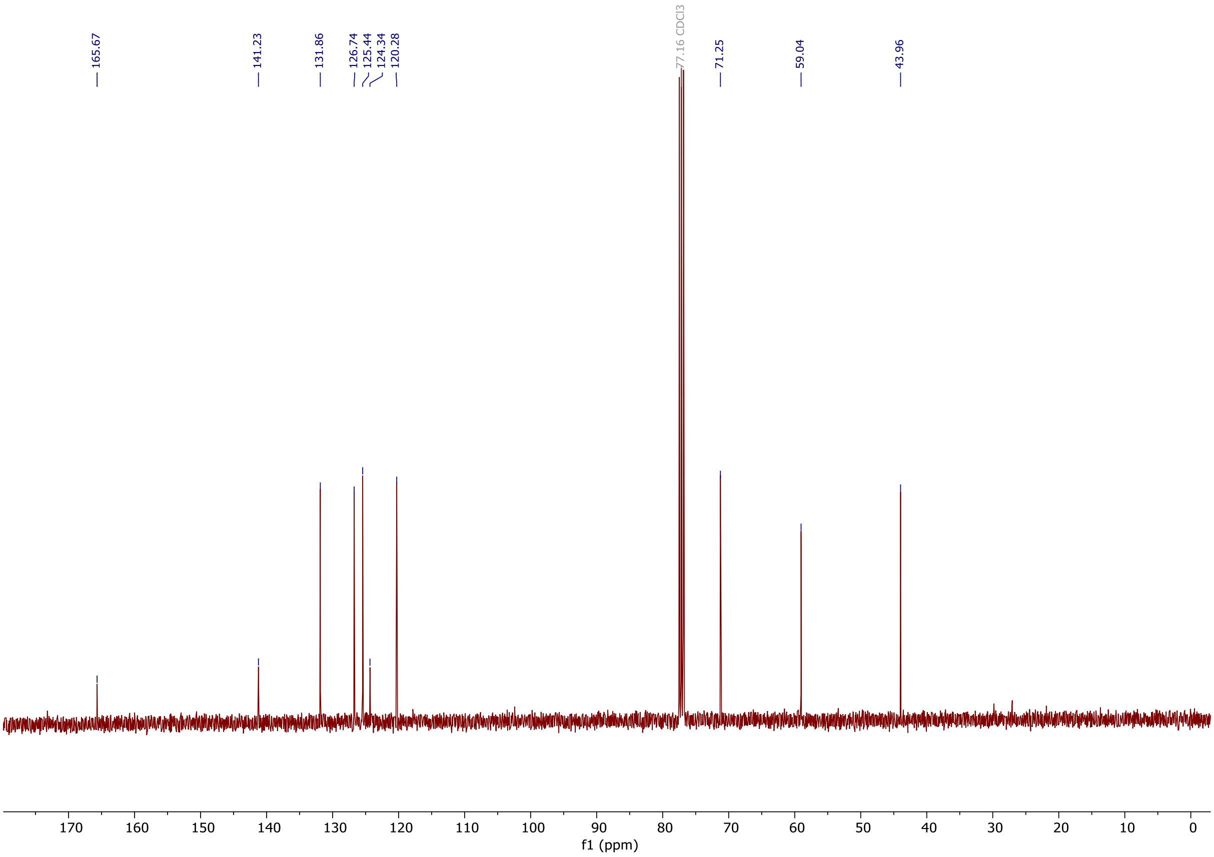

^{1}H NMR of **11** (400 MHz, $CDCl_3$)

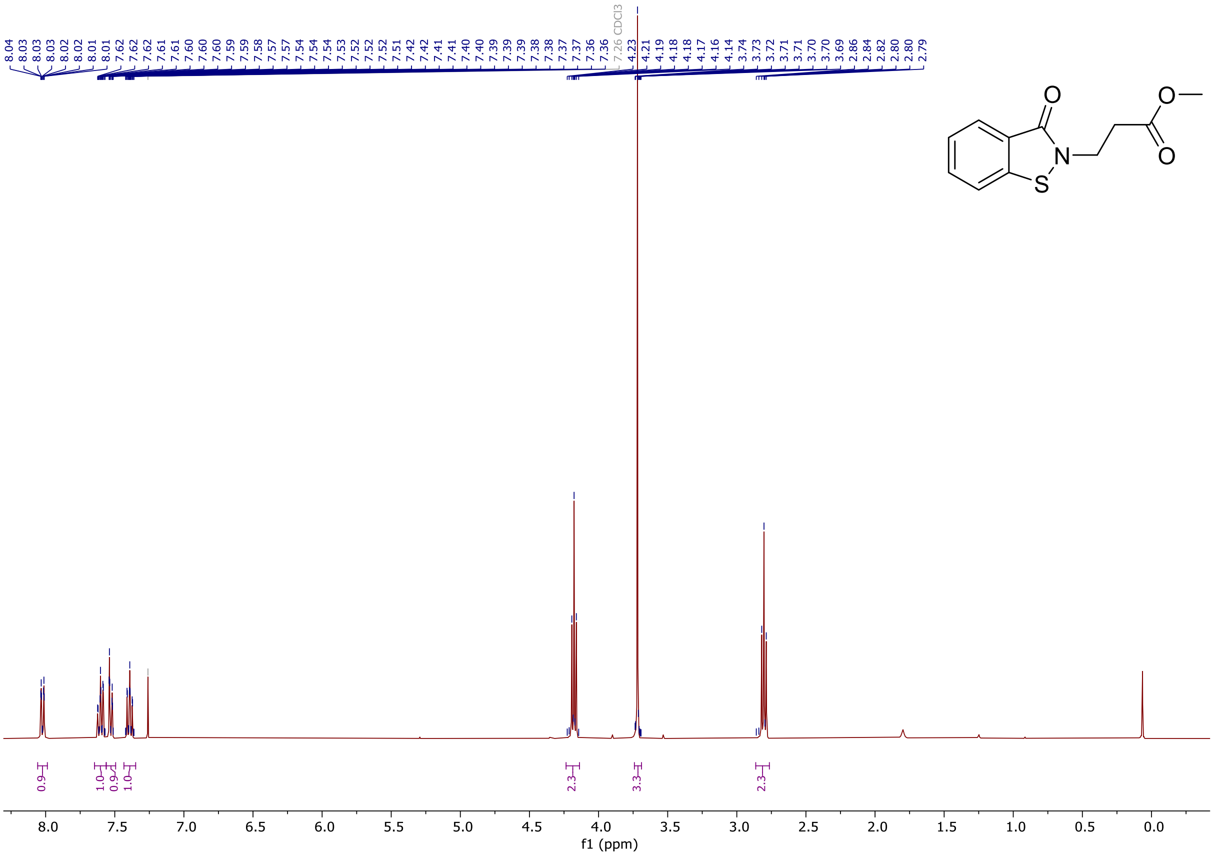

^{13}C NMR of **11** (101 MHz, $CDCl_3$)

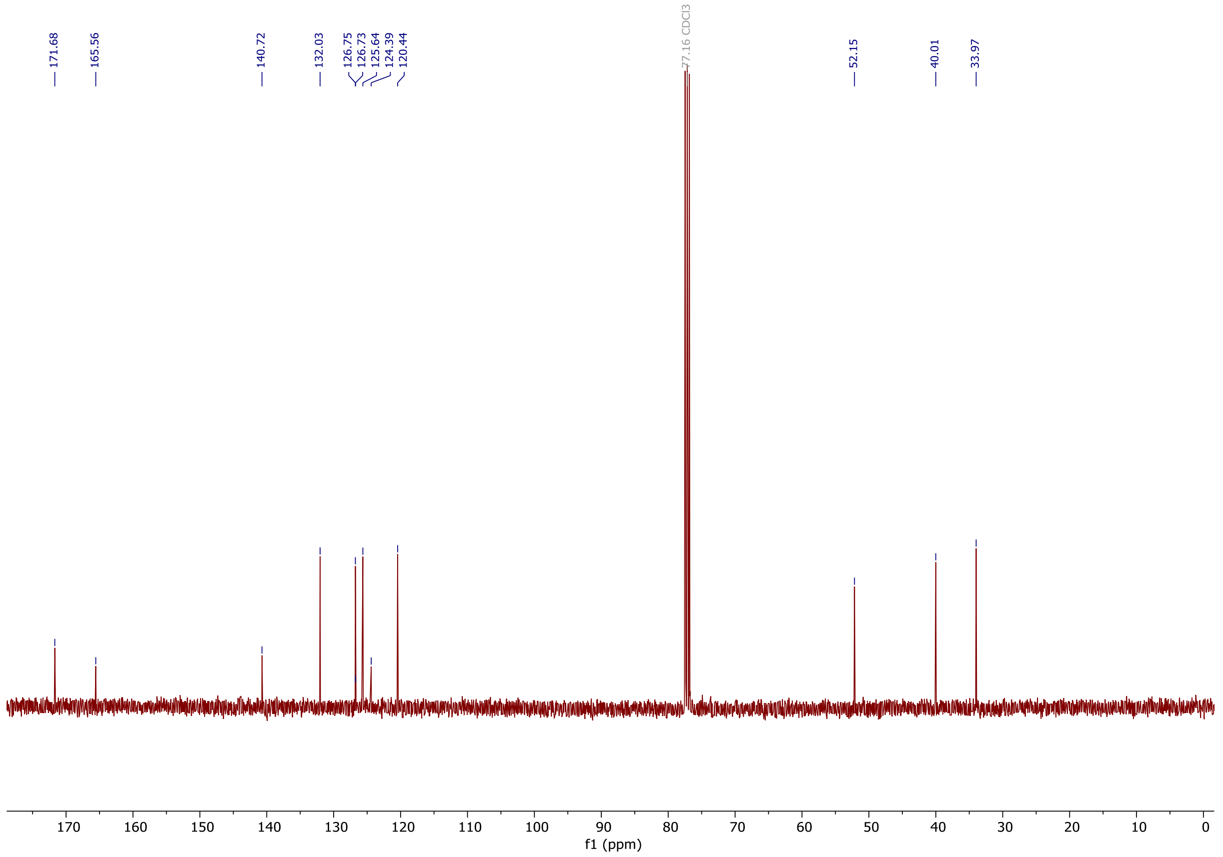
171.68
165.56
140.72
132.03
126.75
126.73
125.64
124.39
120.44
77.16 CDCl3
52.15
40.01
33.97
170 160 150 140 130 120 110 100 90 80 70 60 50 40 30 20 10 0
f1 (ppm)
